# Astatine‐211—Towards In Vivo Stable Astatine‐211 Labeled Radiopharmaceuticals and Their (Pre)Clinical Applications

**DOI:** 10.1002/med.70008

**Published:** 2025-09-01

**Authors:** Marius Müller, Nadia Bom Pedersen, Vladimir Shalgunov, Andreas Ingemann Jensen, Umberto Maria Battisti, Matthias Manfred Herth

**Affiliations:** ^1^ Department of Drug Design and Pharmacology Faculty of Health and Medical Sciences, University of Copenhagen Copenhagen Denmark; ^2^ Department of Clinical Physiology Nuclear Medicine & PET, Rigshospitalet Copenhagen Denmark; ^3^ Department of Chemistry University of Copenhagen Frederiksberg C Denmark; ^4^ DTU Health Technology, Technical University of Denmark (DTU) Lyngby Denmark

**Keywords:** (de)astatination, astatine‐211, astatine‐211 radiochemistry, astatine‐211 radiopharmaceuticals, radioligand therapy

## Abstract

Targeted radioligand therapy has emerged as a promising treatment option for eradicating advanced cancer forms. α‐Emitters are considered particularly promising as they can obliterate (micro)‐metastases. The α‐emitter astatine‐211 (^211^At) has experienced increased interest due to its favorable decay properties. As a result, various ^211^At‐astatination strategies have been developed to address challenges associated with working with this “halogenic metalloid.” This review summarizes efforts to produce and scale ^211^At, describes its physicochemical properties, discusses the advantages and disadvantages of using a radionuclide with a half‐life of 7.2 h and outlines procedures for astatinating radiopharmaceuticals. Moreover, a key focus of this review is to rationalize strategies aimed at minimizing in vivo deastatination. A brief overview of on‐going (pre)clinical development with ^211^At‐labeled radiopharmaceuticals is provided. Astatinated radiopharmaceuticals will play a pivotal role in cancer management in the near future when challenges related to scalability and in vivo stability have been addressed and clinical studies have shown the benefit of ^211^At compared to longer‐lived therapeutic radionuclides.

## 1 Radioligand Therapy (RLT)—A Hope to Treat Cancers More Efficiently

**Figure 1:**
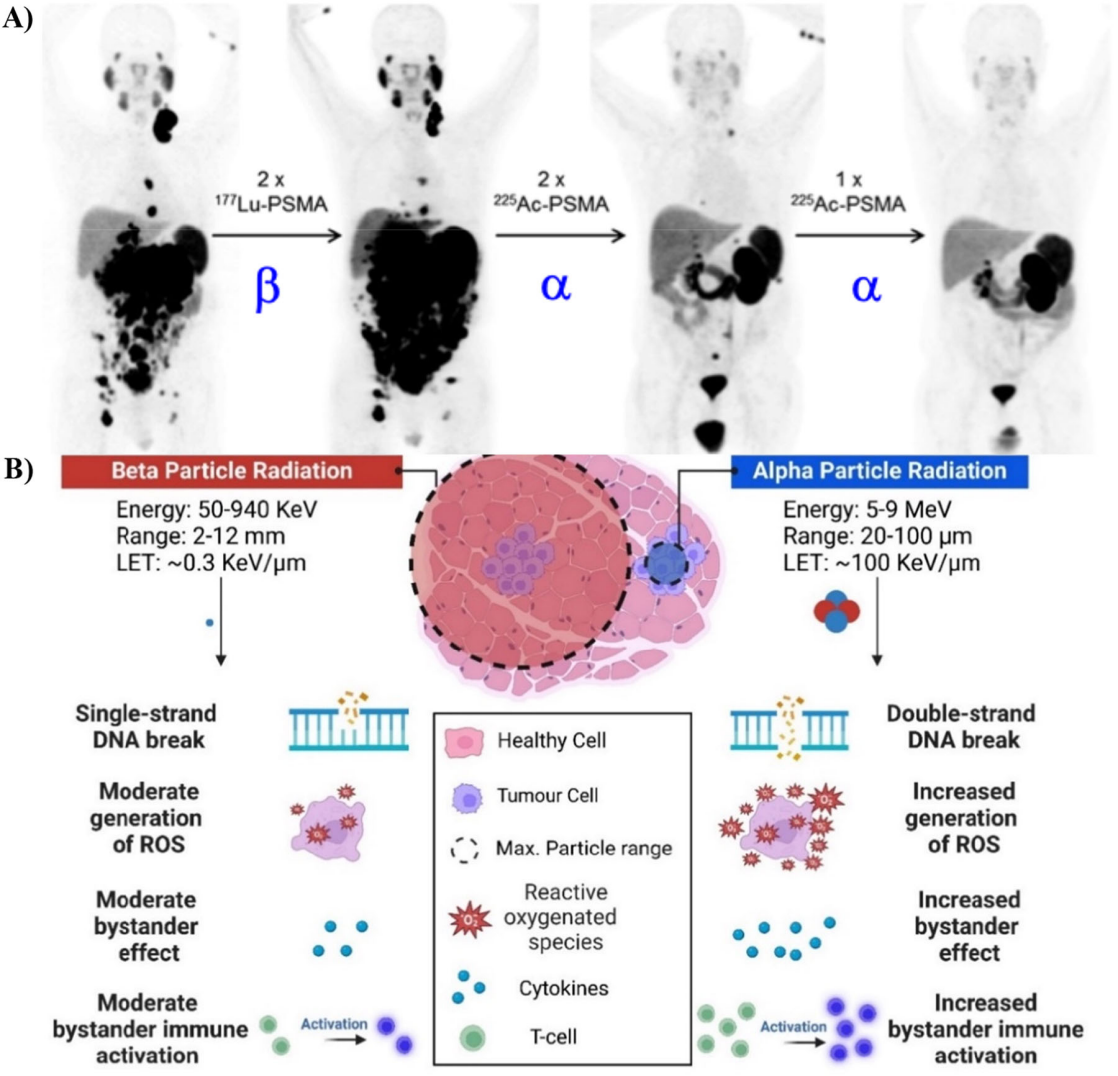
(A) RLT using α‐particles is more efficient than therapies based on β‐particles [7]. The figure is reprinted in accordance with JNM Permission Policies for non‐commercial use. This study was originally published in JNM. Kratochwil et al. [7]. ^225^Ac‐PSMA‐617 for PSMA‐Targeted α‐Radiation Therapy of Metastatic Castration‐Resistant Prostate Cancer. J Nucl Med. 2016; 57: 1941‐1944. © SNMMI. (B) Schematic comparison of the effects of α‐ and β‐particles on biological tissue [8, 9, 10, 11, 12].

Over the last decade, RLT has attracted a lot of interest in oncology, as it has been shown to work where conventional treatment failed [1, 2, 3, 4]. Recently, RLT has also been proven effective as first‐line treatment. For example, the Phase III NETTER‐2 trial—using the radiopharmaceutical ^177^Lu‐Lutathera®—showed a 72% reduced risk of death compared to first‐line treatment with octreotide long‐acting release (Novartis, Jan. 19th, 2024) [3, 5]. Moreover, the Phase III PSMAfore trial showed that pre‐taxane metastatic castration‐resistant prostate cancer patients benefited from ^177^Lu‐Pluvicto^TM^ [1, 6]. The overall response rate was 50.7% and as such approximately 40% higher compared to androgen receptor pathway inhibitor‐based treatments (Novartis, Oct. 23rd, 2023) [6]. These breakthrough therapies are based on radiotherapeutics harnessing β^−^‐emitters, for example Lutetium‐177 (^177^Lu). However, α‐emitters are more efficient at killing cancer cells than β^−^‐emitters (Figure 1). The enhanced treatment effect is based on higher linear energy transfer (LET), and the LET of α‐emitters is approximately 100 KeV/µm, while that of β^−^‐emitters is 0.3 KeV/µm. The LET is defined as the amount of energy deposited from ionizing particles—such as α‐ and β^−^‐particles—into biological tissue per given distance. Consequently, radionuclides with a higher LET can kill cancer cells more effectively than nuclides with a lower LET—when they are selectively targeted to these cancer cells. Selective targeting also results in less irradiation to healthy tissues. Moreover, α‐emitters only travel short distances in tissue, typically between 30 and 70 µm. Consequently, they can selectively kill (micro)metastases in contrast to β^−^‐emitters that have a traveling range of millimeters (Figure 1B).

Radiation has been shown to activate the host's immune system (Figure 1B) [8, 9], and it might be that this activation is even more pivotal in the fight against cancer than the direct ionization effect [13, 14]. There are speculations that α‐emitters may activate the immune system to a higher degree than β^‐^‐emitters. Future studies will show if this is indeed the case.

Thus far, various α‐emitters have been used for RLTs such as terbium‐149 (^149^Tb), astatine‐211 (^211^At), lead‐212 (^212^Pb), bismuth‐212 (^212^Bi), bismuth‐213 (^213^Bi), radium‐223 (^223^Ra), actinium‐225 (^225^Ac), or thorium‐227 (^227^Th) [15, 16]. This review is focused on ^211^At, as we believe that it is the best α‐emitter for most RLTs. Its decay properties, limited waste management challenges, and regulatory hurdles as well as its potentially high production capacity make it unique. Deeper insights into its characteristics and comparison with other α‐emitters are given throughout this review. We will summarize present labeling strategies and discuss their advantages and shortcomings. This review also discusses strategies to minimize in vivo deastatination. Finally, we summarize current efforts to bring ^211^At‐labeled radiopharmaceuticals into the clinic as well as highlight ongoing clinical trials.

## 2 Astatine‐211

**Figure 2:**
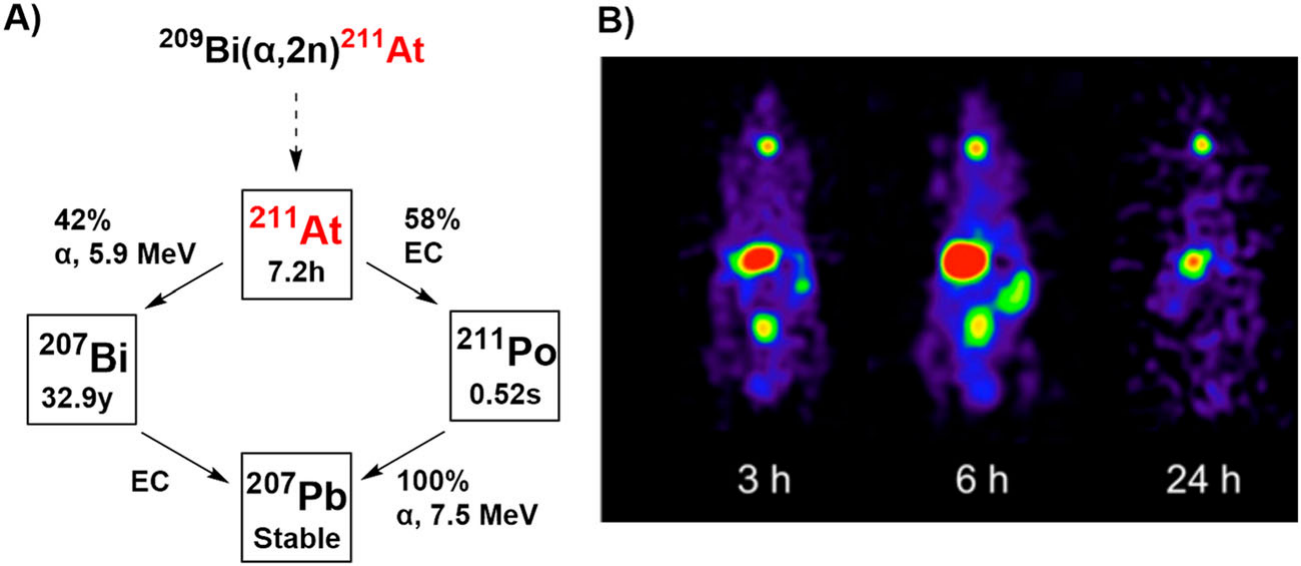
(A) Decay scheme of astatine‐211 (^211^At) [18, 27]. ^211^At decays via two possible decay modes to either bismuth‐207 (^207^Bi) via α‐decay (E_α_: 5.9 MeV) or polonium‐211 (^211^Po) via electron capture (EC). Both ^207^Bi and ^211^Po further decay via EC or spontaneous α‐decay (E_α_: 7.5 MeV) to lead‐207 (^207^Pb). (B) Single photon emission computed tomography (SPECT) image of an ^211^At‐solution in naïve male Wistar rats [28]. The figure is reprinted in accordance with JNM Permission Policies for non‐commercial use. This study was originally published in *JNM*. Watabe et al. Enhancement of ^211^At Uptake via the Sodium Iodide Symporter by the Addition of Ascorbic Acid in Targeted α‐Therapy of Thyroid Cancer. J Nucl Med. 2019; 60: 1301‐1307. © SNMMI.

After tennessine, astatine is the second‐largest halogen known today. No stable isotope of astatine exists. Of its 32 known isotopes, ^211^At exhibits suitable decay properties for RLTs [17]. It decays with a half‐life of 7.2 h to stable lead‐207 (^207^Pb). Two branches of decay exist (Figure 2A), which are accompanied by the emission of α‐particles with an energy of 5.9 and 7.5 MeV, respectively, as well as the emission of a 77–92 keV polonium K X‐ray [18, 19, 20]. This X‐ray allows in vivo imaging of ^211^At‐labeled radiopharmaceuticals [21] or facilitates detection of potential contaminations (Figure 2B). The lack of stable isotopes presents a significant challenge for studying the physical properties and chemical behavior of astatine. Limited knowledge—especially when astatine is incorporated into molecules—makes it difficult to predict the behavior of newly developed structures—particularly regarding metabolic stability. Iodine, the closest stable halogenic analog, is regularly used as a surrogate for astatine. Iodine is believed to exhibit properties similar to astatine [18, 19, 22], even though it lacks the “metallic properties” of astatine [23]. Therefore, iodine does not perfectly mimic astatine's physicochemical properties. Presumably, these metallic characteristics cause ^211^At‐labeled radiopharmaceuticals to suffer from greater instabilities compared to their radioiodine counterparts [18, 24, 25]. However, iodine‐based labeling strategies have frequently been used to establish ^211^At‐chemistry. Additionally, non‐radioactive iodine surrogates are commonly used to validate the identity of astatinated compounds via radio‐HPLC and radio‐TLC. Recently, LC‐MS has been applied in GM‐compliant conditions for the quality control of [^211^At]NaAt at even at a femtomolar concentration, demonstrating that the exact mass of astatinated ions can be accurately determined [26] and suggests a method to validate the identity of astatine radiopharmaceuticals.

### 2.1 The Electronegativity of Astatine‐211

Astatine exhibits both metallic and halogenic properties and is often classified as a metalloid [29]. Some studies have shown that astatine shares more characteristics with polonium—a metal—than with other halogens [29, 30]. Like hydrogen, astatine has an electronegativity of *χ* = 2.20 eV on the revised Pauling scale. The value is in line with the topological analysis of *N*‐succinimidyl‐3‐[^211^At]astatobenzoate ([^211^At]SAB), where astatine has been determined to be positively charged (+0.22 e), making it is less electronegative than carbon (*χ* = 2.55 eV) [31]. Based on density functional calculations (Mulliken scale), the electronegativity of astatine is *χ* = 5.74 eV [32], recently verified experimentally (experimental value *χ* = 5.87 eV) [33]. Also on the Mulliken scale, astatine has a lower electronegativity compared to carbon (*χ* = 6.73 eV) [32]. In agreement with both scales, astatine will be positively polarized in the carbon‐astatine bond and thus have an increased susceptibility to nucleophilic attacks. Interestingly, the electronegativity of astatine is significantly lower than that of hydrogen (*χ* = 7.26 eV) in the Mulliken scale, and thus, hydrogen astatide (HAt) is polarized towards the hydrogen atom, as opposed to all other hydrogen halides [32, 33].

### 2.2 Astatine‐211 Oxidation States

**Figure 3:**
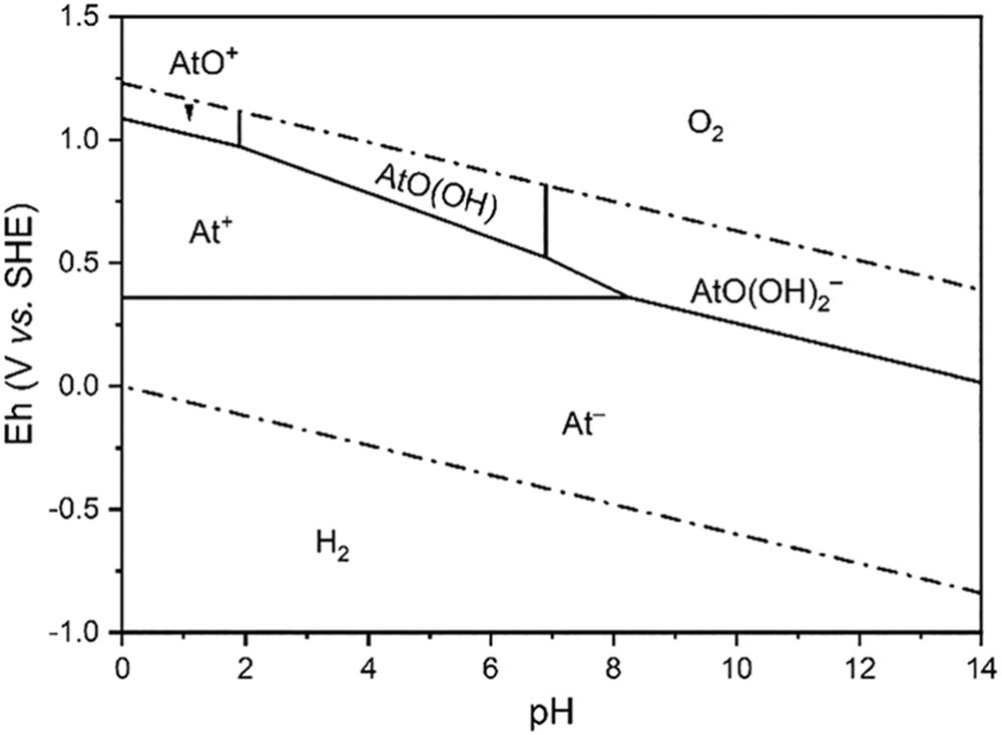
Pourbaix diagram of ^211^At published by Liu et al. [34]. The Pourbaix diagram illustrates the presence of different ^211^At‐oxidation states and their dependency on pH and redox potential (Eh) applied to the system. The figure is reprinted with permission from Liu et al. *Inorg. Chem*. 2022, 61, 13462 – 13470. Copyright (2024) American Chemical Society.

The oxidation states –I, 0, +I, +III, +V are reported for ^211^At. Experimental and computational modeling approaches predict their presence at different pH and redox potentials (Figure 3) [19, 34]. At^−^ (−I) is present under reductive conditions at any pH [22, 34, 35, 36, 37]. Under acidic and stronger oxidizing conditions, both At^+^ (+I) and AtO^+^ (+III) exist [34, 37]. At higher pH values and under oxidizing conditions, the species AtO(OH) (+III) and AtO(OH)_2_^−^ (+III) are described [34, 38, 39]. Astatine with an oxidation state of +V exists at acidic to basic pH under highly oxidizing conditions using potassium persulfate (S_2_O_8_^−^), potassium periodate (KIO_4_), or hypochlorous acid (HClO) [22, 34, 40, 41, 42]. Due to similarities in physical properties of astatine and iodine, and the existence of iodate (IO_3_^−^), it is hypothesized that At(V) may be present as AtO_3_^−^. It is also hypothesized that a sixth oxidation state of astatine exists, namely +VII. It is expected to be formed in the presence of KIO_4_ or xenon difluoride (XeF_2_) in neutral or basic conditions such as AtO_4_^−^ [22, 34, 40, 41]. However, further studies are needed to verify this hypothesis.

## 3 Production and Isolation Techniques for Astatine‐211

**Figure 4:**
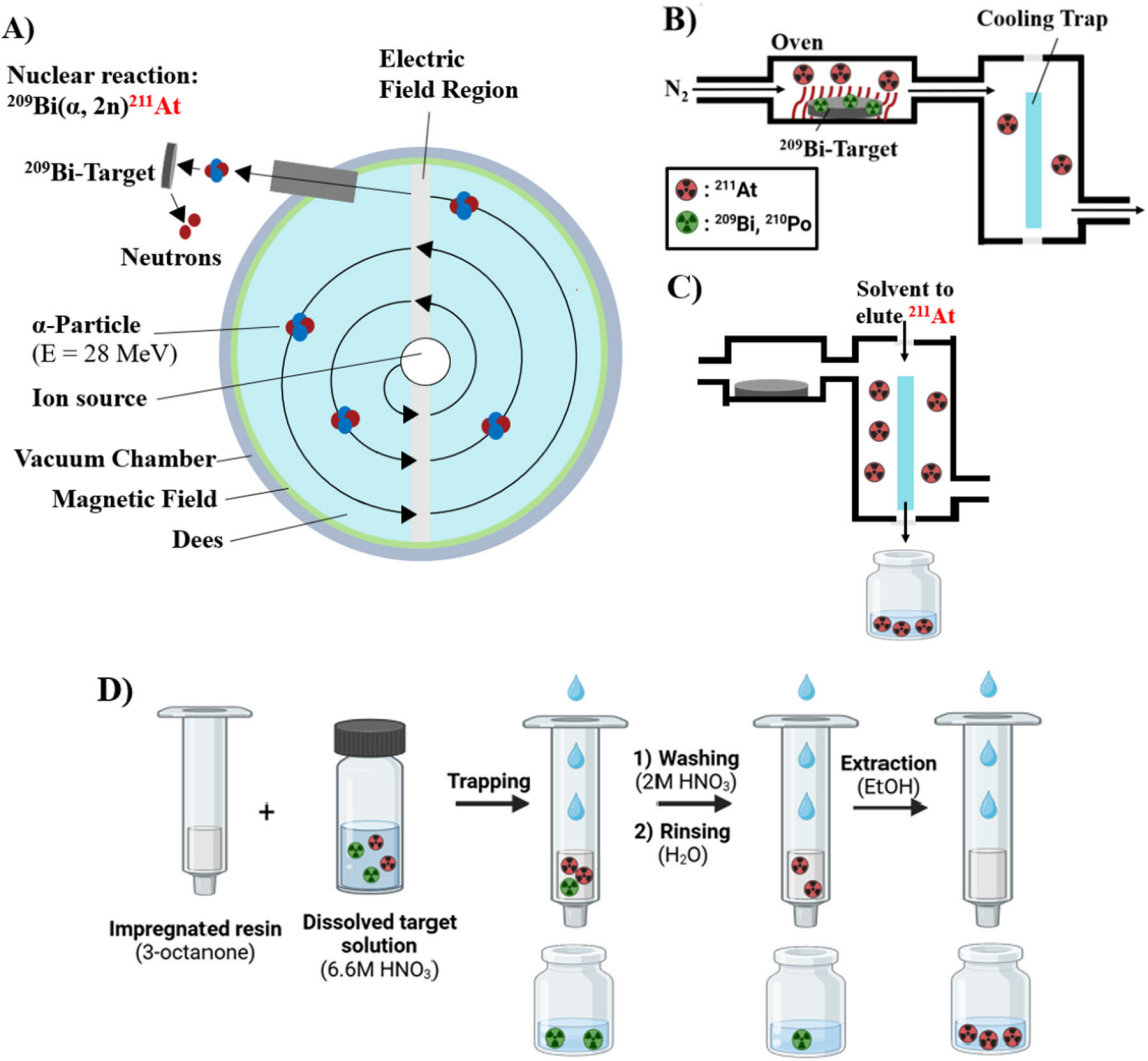
Depiction of the cyclotron production and recovery of ^211^At. (A) Cyclotron production of ^211^At via the ^209^Bi(α,2n)^211^At reaction. α‐particles are accelerated by oscillation of the electrical field region, after which the bismuth target is bombarded to produce ^211^At. (B) For dry distillation, the irradiated target material is heated in a furnace to vaporize ^211^At, which is then trapped onto a cooling trap. (C) ^211^At can be eluted from the cooling trap using a solvent of choice. (D) In liquid‐solid extraction, the dissolved target solution is passed over an impregnated resin. Impurities including ^209^Bi and ^210^Po are removed by washing and rinsing, as they do not interact with the resin. Subsequently, ^211^At is eluted with EtOH [58, 59].

Astatine is the rarest naturally occurring element on Earth and must be artificially produced to isolate sufficient amounts for research or therapy [43]. ^211^At is the only relevant isotope that is used in nuclear medicine, and other isotopes are currently not considered to any larger extent. ^211^At is commonly produced on cyclotrons by irradiating natural bismuth‐209 (^209^Bi) with α‐particles (Figure 4A) [44]. To reduce nuclear side reactions (yielding for example ^210^At), the energy of the α‐particles is set to approx. 28 MeV [27, 44]. After its production, ^211^At is typically isolated from its bismuth target by dry distillation, liquid‐liquid‐ or liquid‐solid extraction. Today, dry distillation (also called “gas thermos‐chromatography”) is the most applied extraction strategy (Figure 4B,C). This strategy makes use of the lower boiling point of astatine compared to that of bismuth [27] and was developed in the 1950s [45]. It has routinely been applied in various laboratories until the 1980s—for example in Illinois (USA), Orsay (France), Pretoria/Johannesburg (RSA), Dresden (GDR), or Dubna (USSR) [46, 47, 48, 49, 50]. Due to the renaissance of radioligand therapies, ^211^At has become of interest these days. Therefore, dry distillation procedures are established at many centers worldwide. In this approach, the irradiated bismuth target is placed into a preheated (600°C–750°C) furnace, and a stream of a noble gas, air or oxygen is passed over the target. Thereby, astatine or its oxidized species are evaporated from the target and transported along with the gas stream directly into a solution of choice, or more commonly, first trapped on a cold surface before rinsed off by chloroform, MeOH or aqueous solutions typically (Figure 4C) [51, 52]. Lindegren et al. [51] refined this process in the early 2000s. Automation and optimization of the dry distillation procedure resulted in recovery yields of 89 ± 2% in less than 20 min [51, 53, 54, 55, 56, 57].

Another method to isolate ^211^At from its ^209^Bi target is based on liquid‐liquid extraction. In this approach, the irradiated bismuth target is dissolved using acid, and subsequently, ^211^At is extracted with diisopropyl ether [60, 61]. Impurities such as polonium and bismuth remain in the acidic aqueous phase. If needed, ^211^At can be extracted back from the diisopropyl ether phase into an aqueous phase at basic pH [60, 61]. The liquid‐liquid extraction method has been reported to yield approx. 80% recovery and typically takes around 2 h [60].

The latest method developed for extracting ^211^At from ^209^Bi targets is liquid‐solid extraction (Figure 4D) [58, 59, 62, 63]. In this method, the bismuth target is dissolved in 6 M HNO_3_ and afterwards passed through a 3‐octanone‐impregnated resin (Amberchrom® CG300M). Under the oxidative environment stemming from HNO_3,_^211^At exists as ^211^AtO^+^. This species is trapped by the impregnated resin, whereas ^209^Bi, ^210^Po, and ^66^Ga/^67^Ga species do not interact with the resin and are simply eluted. The precise interaction of ^211^AtO^+^ and the impregnated resin is not fully understood [59]. However, trapped ^211^AtO^+^ can be almost quantitatively eluted from the resin using EtOH. Recovery yields are approx. 90% within 50 min [59]. Automatization could reduce the procedure time to less than 30 min.

### 3.1 Current and Future Cyclotron Production Capacities: Is There Enough ^211^At?

“Unlike the case with other targeted α‐particle therapy radionuclides, ^211^At supply is not constrained by a dependency on nuclear stockpiles that are heavily regulated and, in some cases, of limited availability, or based on inconvenient target materials” [64]. ^211^At can be produced at high radioactivity levels using inexpensive and readily available target material. This is possible using existing technology (see previous section). However, access is currently limited by the number of active ^211^At production sites.

**Figure 5:**
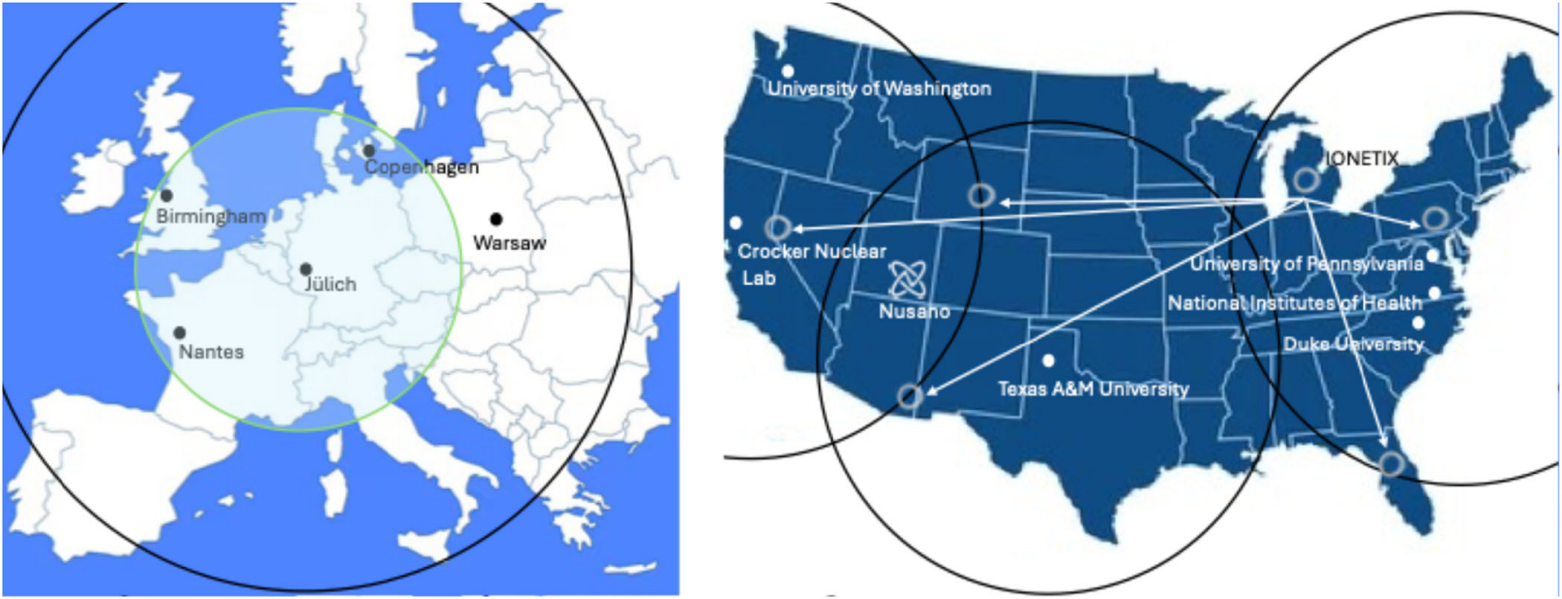
^211^At‐production sites in Europe and the US. IONETIX has announced plans to distribute ^211^At commercially. Nusano has announced intensions to produce ^211^At in TBq quantities using a linear accelerator. The green circle indicates a 1000‐km distribution range reachable by ground transport, while black circles represent a 2000‐km range potentially reachable by air transport. Distribution ranges are only displayed for selected production sites.

To the best of our knowledge, ^211^At has been produced within the last 10 years in following facilities [64, 65, 66]: (1) Duke University Medical Center, Durham, USA (max. 9 GBq), (2) University of Washington Medical Center, Seattle, USA (max. 4.3 GBq), (3) University of Pennsylvania (max. 0.4 GBq), (4) National Institutes of Health, Bethesda, USA (max 1.7 GBq), (5) Texas A&M University, College Station, USA (max. 1.5 GBq), (6) IONETIX, Lansing, USA (max. activity: not reported), (7) Crocker Nuclear Lab, University of California, Davis (test phase, 1.85 MBq), (8) Copenhagen University Hospital, Copenhagen, Denmark (max. 4 GBq), (9) Arronax, Nantes, France (max. 1 GBq), (10) Forschungszentrum Jülich, Jülich, Germany (test phase, max. 0.2 GBq), (11) KIRAMS, Seoul, South Korea (test phase, 32 MBq), (12) RCNP‐Osaka University, Osaka, Japan (max. 3 GBq), (13) QST‐Takasaki, Takaski, Japan (max. 0.3 GBq), (14) QST‐NIRS, Chiba, Japan (max. 1.3 GBq), (15) ICPR Riken, Wako Saitama, Japan (max. 1.3 GBq), (16) Fukushima Medical, Fukushima City, Japan (max. 2 GBq) and (17) Sichuan University, Chengdu, China (max. 0.2 GBq). Figure 5 displays the current ^211^At production facilities in the US and Europe. Future facilities that will produce ^211^At and have been announced to be established or have already been installed are: (I) Alpha Nuclide Medical Technology, Ningbo, China (expected installation in 2045/25), (II) Heavy Ion Laboratory University of Warsaw (POLATOM), Warsaw, Poland (cyclotron installed in December 2023), (III) University of Birmingham, Birmingham, United Kingdom (announced) and (IV) Nusano, West Valley City, Utah, USA (announced).

## 4 Current Landscape of Upscaling ^211^At—Will These Sites Satisfy Future Demand?

This is, admittedly, a challenging question to answer. If one assumes that similar doses of ^211^At will be required as for ^212^Pb, a typical patient dose would be approximately 200 MBq [67, 68, 69]. Assuming, 500 MBq of final product can be obtained per GBq of ^211^At and that cyclotrons will be capable of producing 5 GBq of ^211^At per run in the future, a single site could produce around 2 × 12 patient doses a day. Consequently, 5 production days per week over 50 weeks would result in 6000 patient doses per year. Productions at this scale are achievable with current technology and cyclotron sites [70]. The announcement of RayzeBio to halt enrollment in their ^225^Ac‐based radiotherapy ACTION‐1 trial after radionuclide supplies run scarce (June, 3rd 2024), highlights the urgent need to secure sufficient amounts of activity [71] and to invest in alternative α‐emitting radionuclides. ^211^At production might be a solution to this challenge. As mentioned earlier, Novartis pushed RLT into earlier treatment phases. Therefore, it is expected that several hundred thousand patients—or more—will be treated with radioligands in the future. However, the current and planned ^211^At‐producing infrastructure will most likely not be able to meet this demand. In a very optimistic scenario, the aforementioned facilities might collectively produce 20 GBq of ^211^At a day, resulting in a maximum of 250,000 patient doses per year. However, it is highly unlikely and currently not feasible that all facilities will be produced two times per day, 5 days per week, exclusively for ^211^At‐labeled radiopharmaceuticals. Moreover, it is unrealistic to assume that all facilities will be able to produce 20 GBq per production. A more realistic estimate is that 1/20 of the assumed maximal production capacity can be reached, i.e., 12,500 patient doses per year. Linear accelerators may solve this challenge, anticipated they can produce TBq quantities of ^211^At per day as suggested by Nusano. However, it is clear that investment is needed to scale up ^211^At production. Companies such as IONETIX, Nusano, and Alpha Nuclide Medical Technology have entered this market, but future investment is needed to provide ^211^At commercially, especially in Europe. The announcement of the European company Ion Beam Applications (IBA) to develop a specialized cyclotron for ^211^At production raises the hope that also a ^211^At cyclotron network will be available in Europe. These efforts are supported by the Network for Optimized Astatine labeled Radiopharmaceuticals (NOAR) under COST Action CA19114 supported by the EU. However, despite these positive developments, access to ^211^At remains limited, primarily due to the lack of established distribution networks as well as scarcity of laboratories with the necessary permits and infrastructure to handle ^211^At.

### 4.1 Distribution Capabilities of a Radiopharmaceutical Labeled With ^211^At (Half‐Life: 7.2 h) in Comparison to Those Labeled With ^225^Ac (Half‐Life: 9.9 d)

Despite its 7.2 h half‐life, ^211^At can be distributed effectively within a 1000‐km radius. Figure 5 illustrates that just a handful of cyclotrons in the US and EU would be sufficient to ensure widespread access. A daily production of 5 GBq combined with a 7 h shipment time would allow that delivery of approx. 1.2 GBq of the radiopharmaceutical drug (assuming a 50% radiochemical yield [RCY]). This amount is enough to treat 5–6 patients after 7 h distribution. Such distribution networks already exist for radionuclides with shorter half‐lives, for example, fluorine‐18 (half‐life of 110 min), as seen with PETNET (Siemens) in the US. Air transport, where feasible, would increase the distribution radius to 2000 km. In comparison, distribution of ^225^Ac with a half‐life of 9.92 d also presents challenges, particularly when distributing final products. Current chelator technology cannot re‐chelate daughter nuclides released during the decay chain of ^225^Ac. Even if re‐chelation was possible, the final formulation would contain a mixture of at least six different radiopharmaceuticals with varying pharmacokinetics. These product mixtures would change dynamically over time, making precise product characterization impossible. Regulatory authorities may not approve such product mixtures, especially when better‐defined alternatives with similar or superior efficacy are available. This might be the case for ^211^At‐labeled radiopharmaceuticals. In addition, if daughter nuclides from ^225^Ac are not re‐chelated, highly toxic α‐emitters that are no longer bound to the targeting vector are administered to patients. Alternatively, the final product would be to be re‐purified at the site of application, making distribution of ready‐to‐inject formulations impractical. Even if all mention challenges are resolved in the future, in vivo de‐chelation from ^225^Ac‐labeled radiopharmaceuticals will still result in release of daughter nuclides. Centralized production of ^225^Ac and its distribution to local radiopharmacies introduces additional challenges. Most notably, local sites would require GMP‐compliant laboratories not only to produce the drug but also to separate the daughter nuclides formed during transit.

## 5 Astatine‐211: Advantages, Challenges, and Potential Solutions

### 5.1 Advantages

^211^At emerged as one of the most promising nuclides for RLT. Among its many beneficial characteristics is the broad spectrum of targeting agents compatible with ^211^At labeling chemistry. In the following, we highlight the advantages of ^211^At for drug development and (pre)clinical applications, and we also discuss challenges associated with handling this nuclide.

#### 5.1.1 The Half‐Life of 7.2 h

**Superior waste management**—It is still not clear whether patients will eventually be treated on an outpatient basis or will be required to stay in shielded rooms. However, patients treated with ^225^Ac (half‐life, 9.92 d) cannot be kept at therapeutic centers long enough to collect all their excrement until full decay. Releasing these excrements into the environment may not be a major issue if only a small number of patients are treated. The situation changes significantly if millions of doses are administered annually and a fraction is released into nature [72]. Currently, patients discharged immediately after treatment with ^255^Ac are exempt from national release limits (in some countries) of radioactive substances, such as those excreted renally. However, this policy may change in the near future. To illustrate the scope of this potential shift, we calculated the timeframe in which a patient's urinary excretion would meet national regulatory limits, using modeled data on the renal excretion of a fibroblast activation protein (FAP) inhibitor dimer [73]. Our assumptions include administering 15 MBq of ^225^Ac‐FAPi dimer, with patients urinating approx. 220 mL every 4 h. Each urination is diluted with 11 L of water upon flushing (standard volume in EU lavatories); ***Remarks**: (1) In the US, the flush volume is only 3–5 L, which would negatively impact the calculations; (2) even dilution via flushing may violate national regulations*.

**Figure 6:**
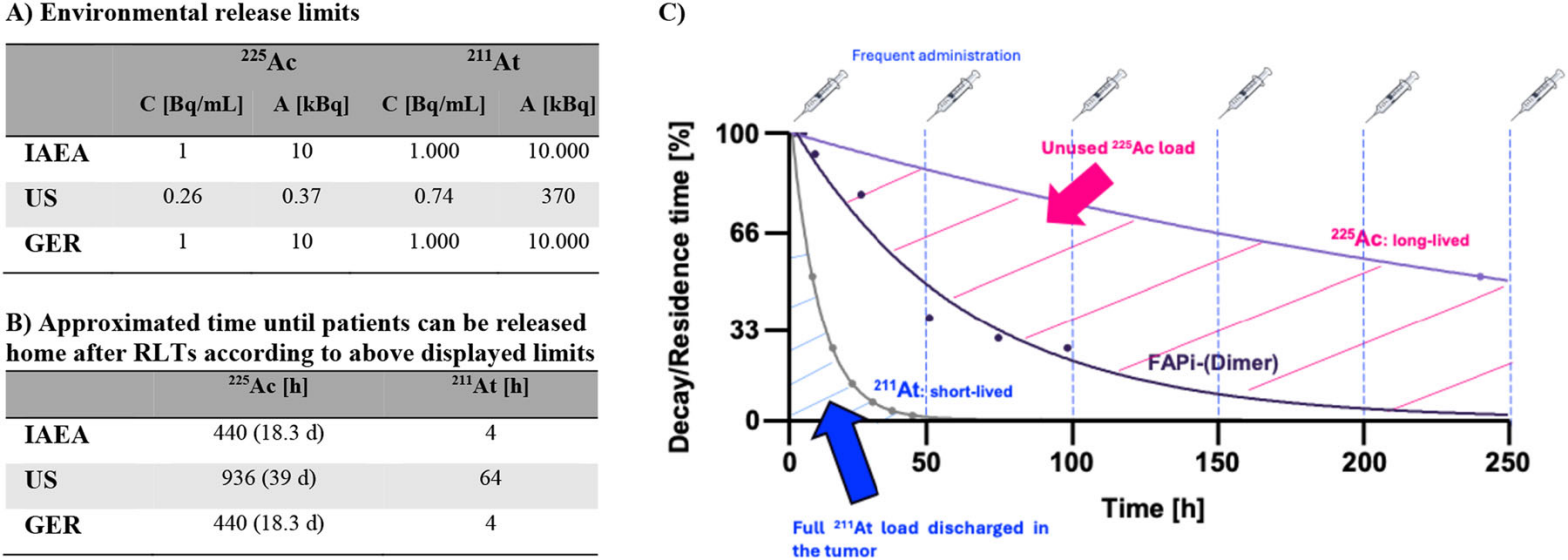
(A) Environmental release limits of ^211^At and ^225^Ac excreted by patients who have undergone RLT (IAEA: International Atomic Energy Agency; US: United States of America; GER: Germany, C: concentration, A: activity). (B) Estimated time until excreted activities from patients undergone RLT meet environmental release limits. Approximations were calculated under the following scenario: Injected activity: 200 MBq for ^211^At and 15 MBq for ^225^Ac); Excretion is only accounted to urination; Patients urinate 220 mL every 4 h (total volume per day: 1.32 L); Isotope concentration was calculated for urine (220 mL) diluted in 11 L toilet water; Scenarios were simulated as a bi‐exponential curve fitted to previously reported experimental data for the FAPi squaric acid dimer [73]. (C) Shorter‐lived radionuclides deliver higher radiation doses to tumors.

Under these assumptions, patients would need to remain in controlled areas for approx. 39 days in the US and approx. 18 days in German to comply with national release limits and guidelines (Figure 6A). Even at a modest daily cost of $500–1000 in the US, this would significantly increase treatment costs. In contrast, when using the same drug labeled with ^211^At (200 MBq administered), and keeping all other parameters constant, patients would only need to remain in controlled areas for approx. 2.5 days in the US and 4 h in Germany (Figure 6B). Release limits for ^207^Bi—the daughter nuclide of ^211^At—would be reached after 4 h in both countries.

The possibility that national authorities will enforce stricter release limits, poses a substantial commercial risk for long‐lived radionuclides such as ^225^Ac, ^131^I, ^177^Lu, or ^161^Tb [72, 74]. In contrast, radiopharmaceuticals with shorter half‐lives—such as ^211^At and ^212^Pb—that are readily available and demonstrate comparable efficacy are likely to be favored by regulatory authorities and may replace their long‐lived counterparts [72, 74].

**Optimizing radiation delivery**

**Part I: Matching radioligand tumor retention with decay half‐live**—Many peptide‐ and small molecule‐based targeting agents show tumor retention of 1–3 days, which does not align well with the half‐live of long‐lived radionuclides. Exemplarily, first‐generation FAP‐targeting agents showed limited tumor retention, with accumulation decreasing by 50% within 6 h post‐injection. Even ^211^At‐labeled FAP inhibitors showed limited efficacy in treating U87MG xenografts [75]. FAP‐targeting radiopharmaceuticals labeled with long‐lived radionuclides have shown minimal or no clinical efficacy, as their physical decay half‐lives do not match the tumor retention of the targeting vector. Consequently, insufficient radiation dose is delivered to the tumor, while increasing the dose would lead to severe side‐effects [73, 76]. Second‐generation FAP‐targeting agents demonstrate significantly improved tumor retention compared to first‐generation drugs (up to 8 d) [75, 77]. These vectors have shown strong tumor‐suppressive effects [73, 77]. The half‐life of ^211^At fits perfectly with these second‐generation agents, enabling full α‐particle dose delivery to the tumor. In contrast, the longer half‐life of ^225^Ac still does not match the tumor retention profile of second‐generation FAP‐targeting agents, limiting its effect dose delivery. Figure 6C displays the decay kinetics of ^211^At in comparison to ^225^Ac, showing that ^211^At can deliver its full dose within the tumor retention window, which is not the case for ^225^Ac.

**Part II: Dose fractionation and potential immune response enhancement**—Dose fractionation has been suggested to improve therapeutic efficacy [78, 79, 80, 81, 82]. Shorter‐lived radionuclides are better suited for this approach, as they can be administered more frequently and at higher doses (Figure 6C). The benefit of dose fractionation may not stem solely from direct radiation damage, but also from enhanced immune activation. Preliminary studies suggest that the immune system needs several days to respond to radiation therapy [78, 79, 80, 81, 82]. Thus, by the time innate or adaptive immune cells would attack the tumor cells, ^211^At would have decayed. This could be advantageous, as α‐radiation may otherwise suppress the immune response by killing immune cells. As such, longer‐lived radionuclides may reduce the immune response. However, these hypotheses require further validation. Most preclinical models use immunodeficient mice, limiting the ability to study immune effects of RLT.

#### 5.1.2 No Serial Decays—The Benefit of “One Decay, One α‐Particle” Compared to Serial Decay

^211^At decays 100% via α‐emission. In contrast to ^225^Ac, exactly one α‐particle is emitted per decay. This is highly beneficial, as it prevents unpredictable dose localization stemming from detachment of radioactive daughter nuclides from the targeting vector [72]. Recent SPECT/CT studies have tried to estimate how much activity is released from the tumor site using [^225^Ac]DOTATATE. In one such study, the first decay daughter ^221^Fr was used as a surrogate for ^225^Ac, while ^213^Bi was imaged directly to estimate how much ^213^Bi had dissociated from the tumor site [83]. While the authors concluded that most of the activity remained at the tumor site (with a slightly increased ^213^Bi uptake in the kidneys and liver), the preliminary SPECT data (reported as time‐integrated activity coefficients) indicated that approximately 20%–25% of ^213^Bi left the tumor, the kidney uptake of ^213^Bi increased 79%, and red bone marrow uptake increased by 65% within 1 week. Time‐activity curves for ^221^Fr and ^213^Bi also indicated a significant increase in liver uptake of ^213^Bi (approx. twofold) [83]. No definitive statements could be made regarding the daughter nuclides ^209^Tl, ^213^Po and ^209^Pb. However, considering the fast decay chain from ^221^Fr (half‐life = 4.8 min) through ^217^At (half‐life = 32 ms) to ^213^Bi and the 20%–25% tumor decrease from ^221^Fr to ^213^Bi, it cannot be excluded that substantial amounts of ^209^Tl, ^213^Po, and ^209^Po are also released from the tumor site. Future research is needed to clarify the consequences of the serial decay of ^225^Ac and its decay products in greater detail. This example clearly shows the advantage of an α‐emitter like ^211^At, which possesses a simpler decay chain.

#### 5.1.3 Covalent Bond‐Forming Atom (Non‐Metal Characteristics)

Astatine shares chemical similarities with iodine, allowing it to be readily incorporated covalently into chemical structures [84]. This unique feature of ^211^At presents a notable advantage over radioligands based on radiometals. Leveraging this property, existing chemical libraries from pharmaceutical companies—where drug structures and their structure‐activity relations (SAR) have already been extensively studied—can be repurposed (**compound repurposing**). Many of these compounds were discontinued because of toxicity concerns. However, radiopharmaceuticals are administered in tracer doses (< 10 µg), which mitigates toxicity risks from chemical interactions [85, 86]. Consequently, drugs with favourable pharmacokinetic profiles could be revived and existing SAR knowledge can be used to develop ^211^At‐labeled radiopharmaceuticals. This strategy is not feasible with chelator‐based structures, as standard SAR studies do not include chelators. Additionally, unlike chelator‐based radiotherapeutics, ^211^At‐labeled compounds can be designed to cross the blood‐brain barrier or bind to intracellular targets. This is possible because ^211^At can be covalently integrated into molecules, allowing the development of (partially) lipophilic agents capable of crossing membranes via passive diffusion.

### 5.2 Challenges

The use of ^211^At does not come without challenges. These relate to its chemistry, the stability of the astatine‐carbon bond, volatility of astatine, and distribution limitations due to its short half‐life. These challenges and their potential solutions are discussed throughout this manuscript.

#### 5.2.1 Astatine‐Carbon Bond Stability in Comparison to Complexation Stability of ^225^Ac

The astatine‐carbon bond exhibits lower bond dissociation energies (BDE) than those for the other halogens, which in some cases leads to deastatination [20, 27]. Free astatine results in off‐target radiotoxicity [18]. While ^211^At^+^ has been reported to accumulate more strongly in thyroid glands than ^211^At^−^, stomach uptake of both species appears comparable [28]. Deastatination can lead to an increase in thyroid uptake from approx. 18 %ID/g at 3 h after injection to 25 %ID/g after 24 h [87]. These rates limit higher dose regimes. However, thyroid accumulation can be partially blocked (e.g., using perchlorate) and novel labeling technologies diminish or even prevent deastatination [88, 89, 90, 91]. In contrast, similar strategies to prevent de‐chelation of ^225^Ac do not exist. De‐chelation and redistribution of daughter radionuclides result in increased uptake in the kidneys, liver, and red bone marrow by approximately 70% (see above). A more detailed discussion on how deastatination can be mitigated or how thyroid uptake of free ^211^At can be blocked is provided later in this review.

#### 5.2.2 Half‐Life Too Short to Match the Pharmacokinetics of Monoclonal Antibodies (mAbs) or Other Nanomedicines

Full‐sized mAbs and nanomedicines typically exhibit accumulation and excretion profiled spanning several days to weeks. The 7.2 h half‐life of ^211^At does not align with these timeframes, making it unsuitable for systemic delivery of ^211^At‐labeled mAbs or nanomedicines. To overcome this mismatch, newer methodologies such as pretargeted or “click‐to‐release” strategies must be applied to match decay half‐life with the pharmacokinetics of these vectors [92, 93]. However, long‐lived radionuclides such as ^225^Ac are also not ideal for systemic application of mAbs or nanomedicines, as their slow excretion can lead to significant radiotoxicity in healthy tissues [92].

#### 5.2.3 Challenges in ^211^At‐Labeling Chemistry

^211^At must be carefully separated from its bismuth target, a process most typically performed via dry distillation. To minimize the risk of contamination or accidental release of volatile ^211^At, strict radiation safety protocols are essential. The distillation procedure is typically performed in glovebox apparatuses designed for specialized radiochemical work. An additional complication is the formation of the daughter isotope ^207^Bi, which has a half‐life of 32.9 years. Although only trace amounts are generated (1 GBq of ^211^At results in 10.9 kBq of ^207^Bi), it can accumulate over time, complicating radioactive waste management and the eventual decommissioning of radiolaboratory equipment. Extensive automatization is required to develop ^211^At‐labeled radiopharmaceuticals. While the chelation of radiometals such as ^225^Ac is less demanding, the degree of automatization for ^211^At is comparable to that for ^11^C‐ or ^18^F‐labeled radiopharmaceuticals, which is well‐established. However, few commercial synthesis modules are available, and most modules are custom‐built. One example is the Swedish company Atley Solutions, which offers a commercial module. More options are expected to emerge as ^211^At‐radiopharmaceuticals have proven successful in the clinic. Unlike chelator‐based radiochemistry, many radiopharmaceuticals—such as peptides, antibodies, proteins or even small molecules—are labeled using synthon‐based approaches. These require mg quantities of precursor materials labeled via the synthon. Consequently, high‐performance liquid chromatography (HPLC) separations are necessary to achieve high molar activities suitable for microdosing. In some cases, separation between precursor and product may not be possible, especially for larger biomolecules where labeling induces a minimal structural change in the overall structural properties. To address this, novel chemistries such as tetrazine ligation are being developed to make ^211^At‐labeling more comparable to radiometal‐based chelation. This allows larger vectors to be labeled indirectly via synthons, which are easier to purify. This will be further discussed later in this review. Additionally, the chemical composition of ^211^At species collected after dry distillation changes over time, likely due to oxidation [22, 94, 95, 96, 97]. This presents a challenge in maintaining a consistent ^211^At species composition to ensure that only the desired species is present. While specific redox agents can help control oxidation states, they may also degrade the precursor or final product. Future research should focus on developing selective methods to control the oxidation state of ^211^At species without compromising the integrity of the targeting vector.

#### 5.2.4 Stability During Distribution

All α‐emitting radiopharmaceuticals face challenges from self‐induced radiolysis, and ^211^At‐based radiopharmaceuticals are no exception. To mitigate radiolytic degradation, formulations must be developed that protect the compound during storage and transport. When shipping free ^211^At in solution, additional challenges arise due to gradual changes in the chemical composition of ^211^At‐species as outlined in the previous paragraphs. To stabilize the oxidation state of ^211^At, suitable redox stabilizers must be added to the formulation. Recently, a novel method utilizing 3‐octanone impregnated Amberchrom® CG300M resin has shown promise. This resin effectively traps ^211^At and allows for its release the following day, preserving the original ^211^At species composition [63].

## 6 Chemistry of Astatine‐211

Several methods have been developed for astatinating radiopharmaceuticals, including nucleophilic or electrophilic astatination, as well as complexation strategies [98]. Figure 3 summarizes the main strategies applied to astatinate radiopharmaceuticals. In this review, RCYs determined by HPLC or TLC will be referred to as radiochemical conversions (RCCs), representing incorporation efficiency. The term RCY will be used exclusively to refer to isolated product activity. RCYs can be influenced by multiple factors, including the efficiency of resolubilizing the radionuclide in the reaction mixture, the RCC into the desired organic or inorganic structure, and the isolation efficiency, which accounts for potential activity losses due to adhesion to equipment such as tubing and cartridges. Therefore, it is important to clearly distinguish between these two terms [99].

### 6.1 Nucleophilic Astatination

Nucleophilic ^211^At‐astatination employs ^211^At in its −1 oxidation state. Astatide ([^211^At]At^−^) is typically generated by reducing positively charged astatine species formed during the isolation process (see earlier discussion on the production and isolation of ^211^At). Standard reducing agents used for this purpose include sodium sulfite (Na_2_SO_3_) and dithiothreitol (DTT).

#### 6.1.1 Astatination of Diaryl Iodonium Salts and Ylides

**Figure 7:**
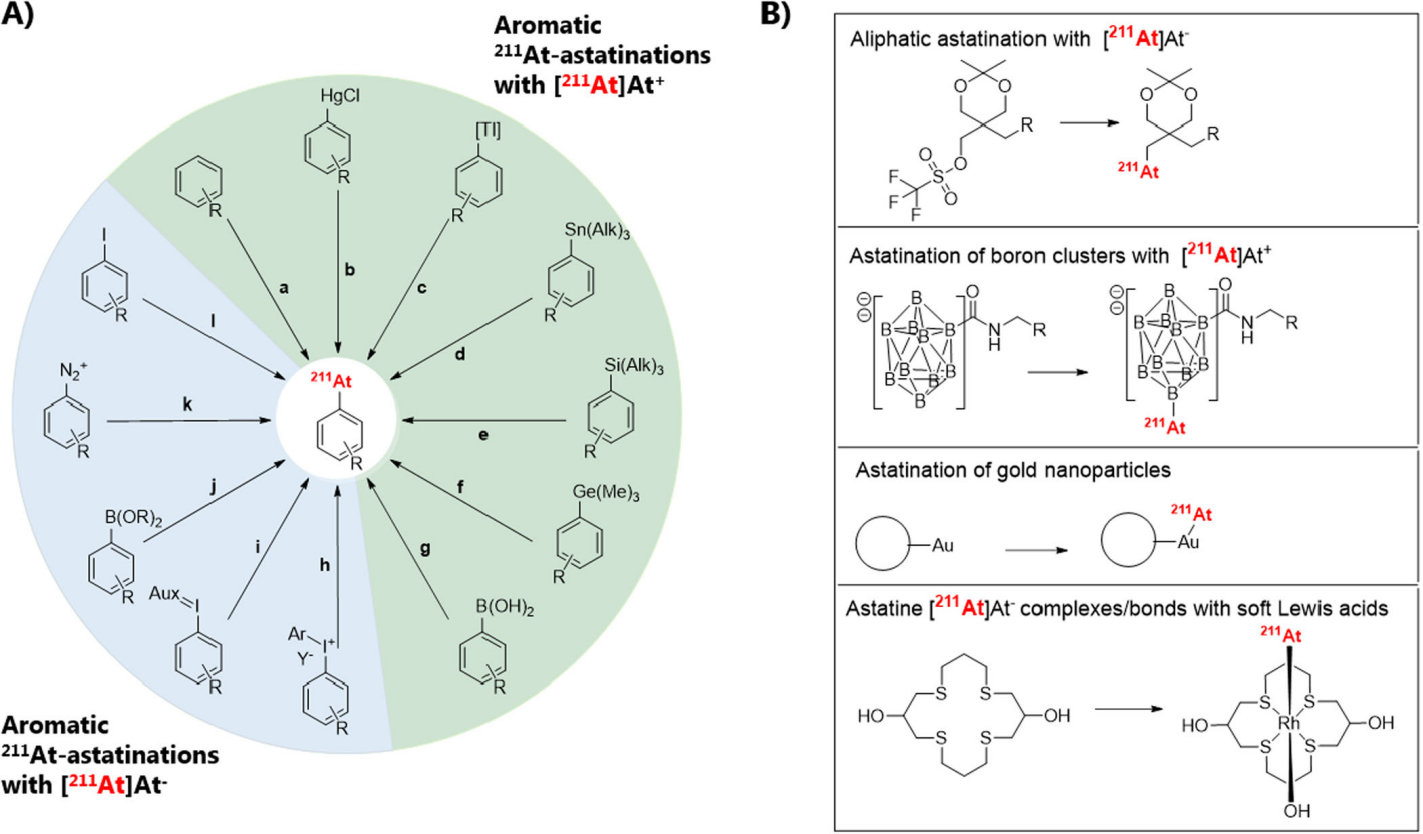
(A) Aromatic ^211^At‐astatination reactions presented in this review. Reactions are categorized based on the use of either [^211^At]At^+^ (green) or [^211^At]At^−^ (blue). **a.** Direct electrophilic ^211^At‐astatination; **b.** ^211^At‐Astatodemercuration; **c.** ^211^At‐Astatodethallation; **d.** ^211^At‐Astatodestannylation (Alk = ‐Me, ‐n‐Bu); **e.** ^211^At‐Astatodesilylation (Alk = ‐Me, ‐Et); **f.** ^211^At‐Astatodegermylation; **g.** electrophilic ^211^At‐astatodeborylation; **h.** ^211^At‐Astatination of aryl iodonium salts (Y^−^ = TfO^−^ or TsO^−^). **i.** ^211^At‐Astatination of aryl iodonium ylides (Aux = Auxiliary). **j.** Cu(II)‐mediated ^211^At‐astatodeborylation (B(OR)_2_ = ‐pinacol boronate (Bpin), ‐boronate (B(OH)_2_)); **k.** ^211^At‐astatodediazoniation; **l.** Cu(I)‐mediated ^211^At‐astatodehalogenation. (B) Non‐aromatic ^211^At‐astatinations and ^211^At‐complexations.

**Figure 8:**
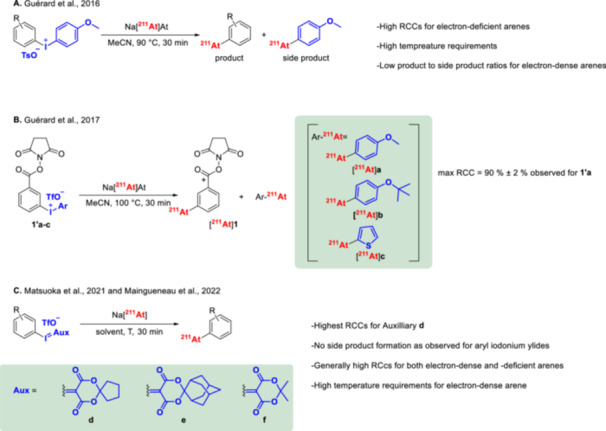
^211^At‐astatination of diaryl iodonium salts and aryl iodonium ylides. (A) Radiosynthesis of ^211^At‐astatoarenes using respective diaryl tosylate triflate salts (leaving group: p‐methoxyphenyliodonium) [103]. (B) Radiosynthesis of [^211^At]SAB ([^211^At]1) using diaryl iodonium triflate salts with different aryliodonium leaving groups [100]. (C) Radiosynthesis of ^211^At‐astatoarenes from respective aryl iodonium ylides with different leaving groups [101, 102].

The formation of astatoarenes can be achieved using aryliodonium ylides or salts as precursors and astatide (Figure 7A, **h,i**) [100, 101, 102, 103]. This methodology was first introduced by Guérard et al. [103] in 2016, utilizing bifunctional diaryl iodonium tosylates containing a p‐anisole iodonium leaving group (Figure 8A). Astatination was performed in MeCN at 90°C for 30 min, using 950 nmol of precursor. The resulting astatoarenes were obtained with high RCCs of up to 99 ± 1%, as determined by radio‐HPLC (Figure 8A). However, the formation of electron‐rich ^211^At‐astatoarenes led to significant side‐product formation—up to a 2:1 product‐to‐side‐product ratio for the formation of 4‐^211^At‐astatotoluene [103]. To minimize side‐product formation, Guérard et al. [100] explored alternative aryliodonium leaving groups, including p‐isopropoxybenzene and thiophene, in 2017 (Figure 8B). [^211^At]SAB was selected as a model compound. Radiolabeling with [^211^At]At^−^ was conducted in MeCN at 100°C for 30 min, using 237.5 nmol of the precursor. The highest RCC (~90%, radio‐HPLC) and minimal side‐product formation (6% compared to 6%–12% for the p‐anisole iodonium‐based leaving group) were achieved with the p‐isopropoxyphenyl leaving group. This result was unexpected, as the p‐isopropoxyphenyl and thiophenyl leaving groups, which have higher electronic density than p‐anisole, were anticipated to reduce side‐product formation more efficiently. In 2021–2022, a new set of iodonium ylides, including cyclopentyl and adamantyl groups, were evaluated as potential precursors for nucleophilic aromatic substitution (Figure 8C) [101, 102]. Maingueneau et al. [102] reported high RCCs (> 60%, radio‐HPLC) using the cyclopentyl leaving group in glyme as the solvent at 90°C for 30 min, while lower RCCs were observed with adamantyl moieties. Similarly, Matsuoka et al. [101] observed RCCs ranging from 60% to 90% (radio‐TLC), depending on the electronic properties of the arene, using cyclopentyl, adamantyl, or Meldrum's acid leaving groups in DMF at 100°C for 30 min.

#### 6.1.2 Astatodeborylation

**Figure 9:**
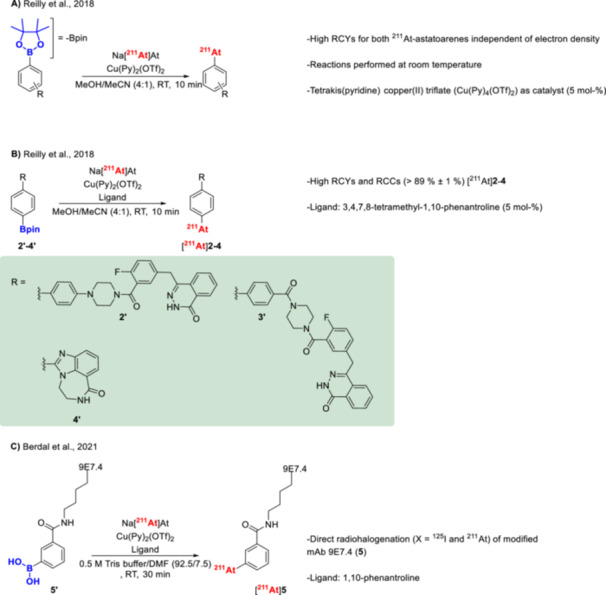
Copper‐mediated ^211^At‐astatination of aryl pinacol boronates. (A) Copper‐mediated ^211^At‐astatodeborylation as reported by Reilly et al. [108]. (B) Copper‐mediated ^211^At‐astatatination of various poly(ADP‐ribose) polymerase inhibitors (PARPi) (**2**–**4**) [108]. (C) Direct copper‐mediated ^211^At‐astatination of mAb 9E7.4 (**5**) [109].

Another method for synthesizing ^211^At‐astatoarenes involves the copper‐mediated astatination of aryl boronic acids and esters with nucleophilic [^211^At]At^−^ (Figures 7A, **j** and 9). Similar copper‐mediated approaches have been published for other radiohalogens and radiocyanide ([^11^C]CN^−^) [104, 105, 106, 107]. This approach was first introduced by Reilly et al. [108] in 2018. RCCs, determined by radio‐HPLC, of up to 99% were obtained for 4‐^211^At‐astatoanisole using aryl pinacol‐, boronic acid‐, and neopentyl glycol boronate precursors. The reactions proceeded with 5 mol‐% of tetrakis(pyridine) copper(II) triflate (Cu(pyridine)_4_(OTf)_2_) and 15 µmol of precursor in MeOH/MeCN (4:1) solvent mixture at room temperature for 10 min. Both electron‐rich and electron‐poor aromatics were successfully radiolabeled with [^211^At]At^−^ in excellent RCCs ranging from 85% to 100% using aryl pinacol boronate precursors (Figure 9A,B). In some cases, the addition of the ligand 3,4,7,8‐tetramethyl‐1,10‐phenantroline was reported to facilitate the reaction [108]. This method has also been applied to directly label the anti‐CD138 mAb 9E7.4 [109]. For labeling, a lysine chain of the antibody was first modified with N‐succinimidyl‐3‐borono‐benzoate, followed by astatination, a RCY of 56%–68% (Figure 9C) [109]. The modified 9E7.4‐aBA (**5’**) was used at a concentration of 32 µM in a solvent system consisting of 0.5 M TRIS buffer/DMF (92.5:7.5), along with 10 mM Cu(OTf)2(Py)4 and 10 mM 1,10‐phenanthroline to successfully label the mAb. Recently, the copper‐mediated ^211^At‐astatodeborylation approach has been applied to radiolabel a PSMA‐targeting vector, achieving an RCY of up to 87% [110].

#### 6.1.3 Astatodediazoniation

**Figure 10:**
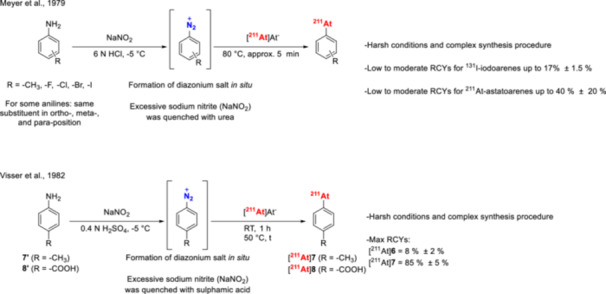
^211^At‐astatination of in situ formed diazonium salts from respective anilines [111, 112].

The reaction conditions for this methodology are relatively harsh and incompatible with compounds sensitive to oxidative or acidic conditions. As a result, its application has been somewhat limited. However, Meyer et al. [111] showed in a proof‐of‐principle study that astatodediazoniation is feasible (Figure 7A, **k**). RCCs, determined via gas‐chromatography after extraction of the reaction mixture, ranged from 10% to 40% for several substituted arenes containing halide‐ or methyl groups (Figure 10) [111]. Building on this study, Visser et al. [112] extended the approach and showed that p‐^211^At‐astatobenzoic acid (^211^At‐**8**) could be radiolabeled with RCCs of up to 85 ± 5%, as determined by radio‐TLC (Figure 10). Finally, Wunderlich et al. [113] reported the synthesis of 1,4‐didiazobenzene for simultaneous ^211^At‐labeling and protein modification. The method allowed ^211^At‐astatination of proteins at room temperature, achieving RCYs of up to 55% [113].

#### 6.1.4 Astatodehalogenation

^211^At has been incorporated into aliphatic or aromatic backbones through astatodehalogenation reactions, as shown in Figure 7, **l**. Visser et al. [114] reported a ^211^At‐halogen exchange method for the radiosynthesis of ^211^At‐labeled tyrosine. In this method, a solution of ^211^At and either 3 mg of iodotyrosine (9.8 µmol) or 3,5‐dioiodotyrosine (6.9 µmol) in water was evaporated to dryness. The residue was subsequently heated to 120°C for 30 min, followed by the addition of 0.5 mL concentrated H_2_SO_4_. The approach resulted in low RCCs of 1%–5%, as determined by paper electrophoresis [114]. In contrast, Liu et al. reported the radiosynthesis of 6‐^211^At‐astatomethyl‐19‐norcholest‐5(10)‐en‐3*β*‐ol (NCL‐6‐^211^At) via an aliphatic astato‐halogen exchange [115]. Their procedure involved reacting 1 mg of the iodine derivative (2.0 µmol) with ^211^At in the presence of 30 mg crown ether, following by heating the mixture at 70°C for 10 min. This method achieved significantly higher RCYs of up to 80%.

***Summary: Nucleophilic* ^*211*^ *At‐astatination***

Various reagents have been introduced as precursors for ^211^At‐astatinations using [^211^At]At^‐^. Among these, aryl iodonium ylides and aryl pinacol boronates have shown particular promise. Aryl iodonium ylides require elevated temperatures (90°C) and extended reaction times (30 min) to achieve high RCCs. In contrast, copper‐mediated ²¹¹At‐astatination of organoboron precursors offers several advantages, including milder reaction conditions and consistently high RCCs of 85%–100%, regardless of the electronic properties of the aromatic substrate [108]. This approach stands out for its versatility, high RCCs, and low toxicity of organoboron reagents, making it one of the most promising strategies for synthesizing ²¹¹At‐labeled arenes. However, as reported by Reilly et al. [108], the method requires relatively high precursor quantities (15 µmol), which may be less practical for developing clinically relevant astatinated radiotherapeutics, where conserving resources and minimizing synthetic effort are critical. Alternatively, Berdal et al. [109] reported that copper‐mediated ^211^At‐astatination of aryl boronates can proceed efficiently at lower precursor amounts (32 µM). Table 1 provides a comprehensive summary of the precursors and methodologies employed for ²¹¹At‐astatination using [²¹¹At]At^−^.

**Table 1:** Summarized labeling characteristics for aromatic ^211^At‐astatinations using [^211^At]At^−^.

Precursor type	Labeling characteristics, key points
Aryl iodonium salts	Requires extensive heating (90°C) to achieve high RCC for electron‐rich ^211^At‐astatoarenes. Significant side‐product formation
Aryl iodonium ylides	Requires extensive heating (90°C) to achieve high RCC for electron‐rich ^211^At‐astatoarenes. No side‐product formation
Aryl boronates	Generally high RCCs, independent of the electron density of resultinhg ^211^At‐astatoarenes Reilly et al.: high precursors amount (15 µmol) required Berdal et al.: improved method using low precursor amounts (32 µM)
Aryl diazonium salts	Low to moderate RCCs at generally harsh conditions Difficult to handle (Caution: Aryl diazonium salts are considered explosive)
Astatodehalogenation	Allows use of widely accessible and commercially available aryl halides Nucleophilic aromatic substitutions generally low yielding: higher yield observed for aliphatic substitutions Often results in reduced apparent molar activities due to challenges in separating precursor from the labeled product

### 6.2 Electrophilic [^211^At]At^+^: Electrophilic Aromatic Substitution

In electrophilic aromatic substitutions (SEAr), astatine in the +1 oxidation state ([^211^At]At⁺) is employed. Oxidation of isolated ^211^At is commonly achieved by adding N‐chlorosuccinimide (NCS), chloramine‐T or peroxides. Stronger oxidizing agents, such as peroxydisulfate ion (S₂O₈²⁻), have also been reported to facilitate the formation of the [^211^At]At⁺ species. The introduction of electrophilic [^211^At]At⁺ into an aromatic backbone is most often accomplished through astatodemetalation, involving metal‐containing groups such as those of silicon, tin, thallium, mercury, or germanium (Table 2) [27, 87, 95, 97, 114, 116, 117, 118, 119, 120, 121, 122, 123, 124, 125, 126, 127, 128, 129, 130, 131, 132, 133, 134, 135, 136, 137].

**Table 2:** Reported strategies for electrophilic aromatic ^211^At‐astatination. (A) Direct electrophilic ^211^At‐astatination. Metalation‐based strategies using: (B) mercury, (C) thallium, (D) tin, (E) silicon, (F) germanium, and (G) boron. Oxidants = NCS, Chloramine T, peroxides, for example.

Entry	Labeling	Characteristics, key points
A	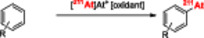	Harsh reaction conditions (high temperatures required) No information on substitution pattern, may depend on electron‐density
B	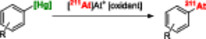	Highly toxic organomercury precursors In situ formation of organomercury precursor The substitution pattern may vary (e.g., ortho‐ and para‐position for electron‐dense arenes)
C	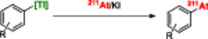	Highly toxic organothallium precursors In situ formation of organothallium precursor Substitution pattern may vary (e.g., ortho‐ and para‐position for electron‐dense arenes)
D	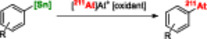	Mild reaction conditions, Generally high RCYs High reactivity toward [^211^At]At^+^ Acid‐labile and toxic precursors
E	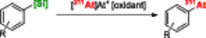	Harsh reaction conditions (TFA solvent and elevated temperatures), Generally high RCYs Low reactivity towards [^211^At]At^+^ Low acid‐sensitivity and toxicity precursors
F	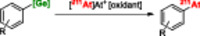	Generally harsher reaction conditions (TFA solvent), Generally moderate to high RCCs Moderate reactivity towards [^211^At]At^+^ Acid sensitivity and toxicity between those of tin‐ and silicon‐derivatives
G	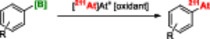	Generally mild conditions Low toxicity of precursors

#### 6.2.1 Direct Electrophilic Aromatic Substitution

Direct electrophilic ^211^At‐astatination (Table 2A), via an astatine‐hydrogen exchange, was initially reported by Vasaros et al. [116]. Astatination of benzene were performed under highly oxidative conditions, using dichromic acid (H_2_Cr_2_O_7_) and perchloric acid (HClO_4_) [116]. RCYs of up to 45% were achieved by heating the reaction mixture to 100°C for 90 min. A significant increase in RCC (approx. 90%, as determined by paper chromatography) was observed when aqueous HClO₄ or sulfuric acid (H₂SO₄) was used as oxidizing agent at 180°C–190°C for 20 min [117]. These conditions were also applied to radiolabel [^211^At]astatotyrosine resulting in a RCY of approx. 90%. However, decomposition of [^211^At]astatotyrosine occurred at temperatures above 190°C, while no product formation was observed below 120°C [118]. No information was reported regarding the substitution pattern of the resulting product. In general, this labeling strategy is limited to molecules that are stable under strong oxidative conditions and high temperatures, which has restricted its broader application.

#### 6.2.2 ^211^At‐Astatination Using Aromatic Mercury Compounds

The first reported demetallation strategy for introducing ^211^At into an aromatic framework involved mercury‐containing compounds, such as chloromercury groups (Table 2B) [121]. Regardless of the electron density of the arenes, RCCs were generally high ranging between approx. 65%–95%, as determined by electrophoresis or activity distribution via extraction. Labeling was successful at low to moderate temperatures (room temperature to 60°C), typically within 30 min after formation of the precursor. This method has been used to radiolabel pyrimidines, nucleosides, DNA, RNA, steroids, imidazoles, and tyrosines [114, 119, 120]. Compared to direct electrophilic ^211^At‐astatination, this approach can be performed under milder conditions. However, its application is limited by the high toxicity of the organomercury species, necessitating careful purification and quality control. In some cases, mercuriation has been reported to yield different stereoisomers. For example, mercuriation of aniline led to the introduction of the chloromercury group at both the ortho‐ and para‐positions [121].

#### 6.2.3 ^211^At‐Astatination of Organic Thallium Compounds

Thallation followed by ^211^At‐astatination of benzoic acid and anisole has been reported by Visser et al. [122] in 1982 (Table 2C). Thallations were performed using thallium(III) trifluoroacetate in trifluoroacetic acid (TFA). After concentrating the reaction under reduced pressure, either water was added to the thallated anisole or 0.4 N H₂SO₄ to the thallated benzoic acid, along with ^211^At and 1.5 equivalents of potassium iodide. RCYs of 70%–90% were obtained for 2‐[^211^At]‐astatobenzoic acid and 4‐[^211^At]‐astatophenol [122]. Thallation of benzoic acid predominantly occurred at the ortho‐position, while thallation of the phenol primarily occurred in the para position when short reaction times were used [122]. In contrast to mercuration, thallation requires stronger oxidative conditions, limiting its application to precursors that are resistant to oxidation. Additionally, organic thallium compounds are highly toxic and must be handled with extreme caution.

#### 6.2.4 ^211^At‐Astatodestannylation

The use of organotin compounds as precursors for ^211^At‐astatinations was first introduced by Milius et al. [123] in 1986 (Table 2D). Since its initial report, ^211^At‐astatodestannylation has become one of the most widely applied methods for synthesizing astatoarenes. Unlike mercuriations and thallations, trialkylstannyl groups can be selectively introduced into arenes via palladium‐catalyzed stannylation of aryl bromides or iodides with hexamethylditin, or by reacting aryl Grignard, zinc, or lithium reagents with trialkyltin chloride [138, 139, 140]. Astatodestannylation has been successfully performed in various solvent systems, such as acetic acid and methanol, and in combination with several oxidants (e.g., chloramine‐T, *N*‐chlorosuccinimide, peroxides [27, 95, 123, 127, 128]). Electrophilic astatination of trialkylstannyl precursors is one of the most promising and high‐yielding methods for introducing ^211^At into an aromatic backbone. However, organotin precursors are highly toxic, which presents challenges for their use in good manufacturing practice (GMP) environments. Furthermore, these precursors exhibit low stability under acidic conditions, complicating the synthesis of certain ^211^At‐astatoarenes that require acidic deprotection during precursor synthesis [141, 142].

**Figure 11:**
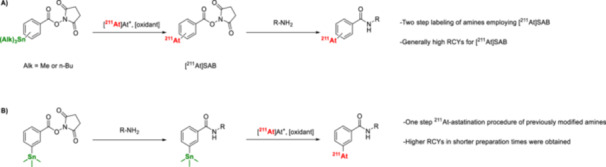
Protein‐ or mAb‐labeling using prosthetic groups based on *N*‐succinimidyl‐3‐(trialkylstannyl)benzoate. (A) Conventional two‐step approach for astatinating proteins and mAbs. Radiosynthesis of [^211^At]SAB via electrophilic astatodestannylation, followed bymodification of amines (e.g., present in proteins and mAbs) [127, 128, 129, 130, 131, 132, 133]. (B) One‐step approach for astatinating proteins and mAbs. Modification of amines with *N*‐succinimidyl‐3‐(trialkylstannyl)benzoate, followed by the electrophilic astatination [124, 137, 143].

Astatodestannylation has been widely applied to producing various radiopharmaceuticals. One notable application is the astatination of the prosthetic group *N*‐succinimidyl‐3‐(stannyl)benzoate, which facilitates the modification of biomolecules such as antibodies, proteins, and other targeting vectors (Figure 11A) [127, 128, 129, 130, 131, 132, 133]. This method enables the subsequent coupling of *N*‐succinimidyl‐3‐[^211^At]astato‐benzoate and its derivatives to biomolecules, resulting in ^211^At‐labeling with overall RCYs ranging from approximately 26%–66% [127, 128, 129, 130, 131, 132, 133].

While aforementioned prosthetic groups have proven successful, their use in a two‐step procedure is not optimal due to the increased complexity, which hinders automation and scalability. As a result, direct incorporation of ^211^At into biomolecules has been explored. For example, lysine residues within mAbs have been modified with *N*‐succinimidyl‐3‐(trialkylstannyl)benzoate to incorporate a precursor moiety that can be directly labeled with ^211^At (Figure 11B) [124, 137, 143]. Lindegren et al. [143] reported that this approach resulted in high RCYs of 85%–89% of a ^211^At‐labeled trastuzumab conjugate. Despite the higher RCYs achieved through this single step radiolabeling technique compared to the two‐step methods involving *N*‐succinimidyl‐3‐[^211^At]astato‐benzoate, significant nonspecific incorporation of ^211^At (30% of the initial activity) into naïve trastuzumab was observed [143]. To expand the scope and avoid reliance on lysine residues, alternative prosthetic groups, such as *N*‐[2‐(maleimido)ethyl]‐3‐[^211^At]‐astato‐benzamide, have been developed and applied to an anti‐HER2 antibody. Modification of cysteine residues with the corresponding organotin precursor, followed by ^211^At‐astatination, resulted in RCYs of 60%–80% for the labeled antibodies [134, 137].

**Figure 12:**
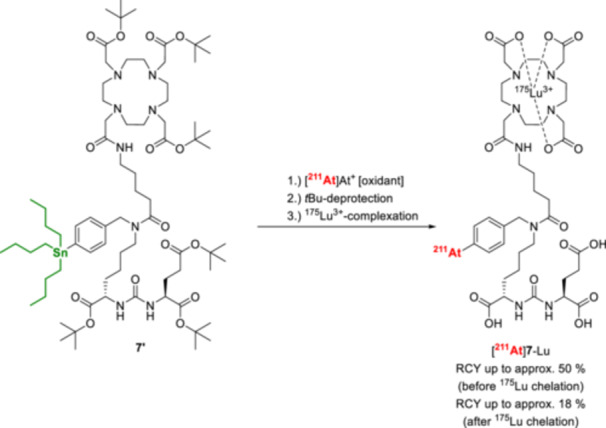
Radiosynthesis of the PSMA‐targeting vector [^211^At]**7**‐Lu [125].

Direct astatination of small molecules has become a major application of the demetallation strategy using trialkylstannyl precursors. For example, a series of PSMA‐targeting structures, such as [^211^At]**7**‐Lu, have been labeled with ^211^At in RCYs of up to 21 – 63% (before ^175^Lu‐chelation for [^211^At]**7**‐Lu) starting from organotin precursors (Figure 12) [125, 126].

#### 6.2.5 ^211^At‐Astatodesilylation

**Figure 13:**
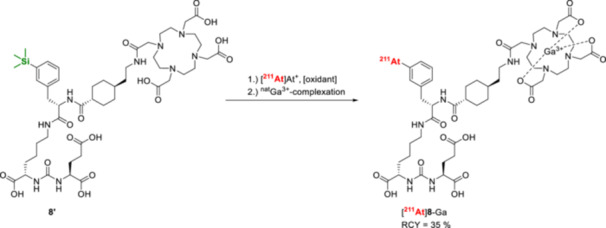
Radiosynthesis of the PSMA‐targeting vector [^211^At]**8**‐Ga [145].

In contrast to organotin precursors, their silicon‐based analogs exhibit greater stability against protodemetalation under acidic conditions [135] and are also associated with lower toxicity [144]. However, astatination of aryl trialkyllsilanes requires harsher conditions due to their lower reactivity toward electrophiles (Table 2E) [141, 142, 145]. For example, astatodesilylations have been performed in TFA at 70°C for 10 min, achieving satisfactory RCCs of over 70% [141, 142, 145]. Due to the increased inertness of the trialkylsilyl group, precursor synthesis can tolerate acidic deprotection conditions [142, 145], which is particularly advantageous for peptide synthesis. Notably, ^211^At‐astatination of a deprotected triethylsilyl precursor was found to be superior for the synthesis of [^211^At]‐4‐astato‐l‐phenylalanine ([^211^At]‐**APA**), compared to the protected tributylstannyl precursor followed by subsequent deprotection [142]. Astatodesilylation represents an attractive alternative to label acid‐insensitive radiopharmaceuticals. For example, a PSMA targeting vector [^211^At]**8**‐Ga was successfully astatodesilylated with an overall RCY of 35% after gallium‐chelation (Figure 13) [145]. This labeling strategy involved synthesizing Fmoc‐3‐trimethylsilyl‐l‐phenylalanine and incorporating the precursor into a peptide‐based PSMA‐targeting structure via solid‐phase peptide synthesis (SPPS). Since the trimethylsilyl group is stable under acidic deprotection conditions—such as exposure to 4 M HCl in dioxane—its compatibility with SPPS offers significant potential for labeling peptides that are acid‐resistant, without relying on prosthetic groups like [^211^At]SAB.

#### 6.2.6 ^211^At‐Astatodegermylation

**Figure 14:**
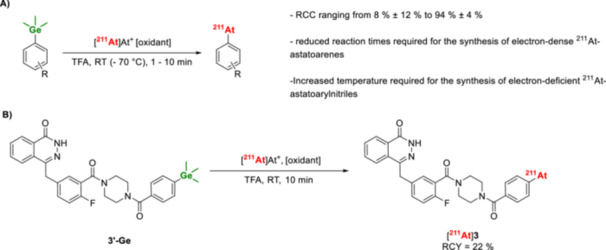
^211^At‐astatination of aryltrimethyl germanes. (A) General conditions for ^211^At‐astatodegermylation. (B) Radiosynthesis of [^211^At]**3** from the aryl trimethylgermane precursor **3’‐Ge**.

Germanium‐based precursors have recently been shown to be effective for ^211^At‐astatodegermylation of arenes (Table 2F). This approach builds on previous studies that utilized organogermanium compounds as precursors for radioiodination [146, 147]. Inspired by these findings, Müller et al. [136] investigated the suitability of these precursors for astatodegermylation. Reactions were conducted in TFA at room temperature or at 70°C for 10 min, using 0.15 µmol of precursors. Electron‐poor astatoarenes were labeled with moderate to high RCCs of 55%–94%. In contrast, electron‐rich arenes were labeled in low RCCs under the same conditions, likely due to ^211^At‐protodeastatinations. To address this limitation, the authors hypothesized that reducing the reaction time might improve RCCs. Indeed, shortening the reaction time from 10 to 1 min significantly increased RCCs for electron‐rich astatoarenes, improving the RCC from 14% to 93% [136]. Müller et al. [136] further demonstrated the utility of this method by applying it to the ^211^At‐astatination of a PARPi, achieving an isolated RCY of 22% (Figure 14) [108, 136]. Compared to astatodestannylation and astatodesilylation, astatodegermylation exhibits reactivity that lies intermediate between stannyl‐ and silyl‐based precursors [147].

#### 6.2.7 Astatodeborylation

Shirakimi et al. [97] were the first to demonstrate that electrophilic astatodeborylation (Table 2G) is a viable approach for incorporating ^211^At into aromatic frameworks, successfully labeling [^211^At]‐**APA**. Unlike most previously reported methods, these reactions were conducted in water. Interestingly, electrophoresis and radio‐TLC analyses revealed that ^211^At exists in water in multiple oxidation states, including At(‐I), At(0), At(I), and At(III) [97]. Due to the presence of oxidized ^211^At species, the authors evaluated two different approaches for electrophilic aromatic ^211^At‐astatinations. Reactions using the aryl boronate precursor (0.1 mg and 0.48 µmol), NBS as oxidant, and sodium bicarbonate as an additive at room temperature for 30 min yielded [^211^At]‐**APA** in a RCC of 90.8 ± 2.7% [97]. Interestingly, replacing the NBS with KI further improved the RCC of [^211^At]‐**APA** to 98.1 ± 1.9%, suggesting the predominance of oxidized ^211^At species in water. The authors proposed that KI facilitated the formation of electrophilic ^211^At species, such as [^211^At]AtI or [^211^At]AtI_2_, thereby enhancing reaction efficiency [97]. Using the same KI‐based procedure, the group extended this method to astatination of various PSMA‐targeting vectors via electrophilic astatodeborylation, achieving RCYs of at least 60% [87]. However, unlike the radiosynthesis of [^211^At]‐**APA**, these ^211^At‐astatinations required heating to 80°C and extended reaction times of up to 45 min to achieve optimal yields [87]. Electrophilic astatoborylations (Table 2G) have emerged as an attractive option in the radiochemist's toolbox due to their low toxicity, copper‐free conditions, and generally high RCYs. Notably, 4‐borono‐l‐phenylalanine was successfully used as a precursor for synthesizing [^211^At]‐**APA**, demonstrating the compatibility of the boronate group with standard acidic and basic deprotection conditions. This compatibility facilitates the synthesis of more complex precursors, such as peptides, thereby expanding the versatility of this methodology.

Improved RCCs for electron‐rich astatoarenes have been achieved by reducing reaction times, highlighting the potential for further optimization [136]. Overall, organogermanium precursors offer a compelling balance of low toxicity, chemical stability, and reactivity—traits that position these precursors between organosilicon and organotin compounds. These properties make them well‐suited for early‐stage incorporation into complex molecules [136]. Similarly, organoboron precursors show significant promise due to their low toxicity and relatively high reactivity towards [^211^At]At^+^, allowing reactions to be performed in water. This unique compatibility makes organoboron compounds particularly attractive for streamlined ^211^At‐radiolabeling applications [87, 97].

**Summary: Electrophilic ^211^At‐astatinations**

Among the various electrophilic ^211^At‐astatination strategies, the use of organosilicon, organogermanium, and organoboron precursors stands out as particularly promising. These approaches offer reduced toxicity compared to traditionally used organotin reagents and have been successfully applied to the astatination of complex molecules, including PSMA inhibitors, PARP inhibitors, highly reactive H‐tetrazines, and amino acids [135, 136, 141, 142, 144, 150]. Organosilicon precursors, while exhibiting low toxicity and high stability, require relatively harsh conditions for astatination, such as TFA at 70°C [135, 141, 142]. In contrast, organogermanium precursors enable radiolabeling at room temperature, albeit still in the presence of TFA, facilitating the synthesis of ^211^At‐labeled scaffolds that are otherwise difficult to access. Unlike organosilicon and organoboron reagents, organogermanium‐based astatination has been applied to a broader range of substrates. However, this versatility also reveals a limitation: electron‐rich substrates are prone to proto‐deastatination under acidic conditions, leading to lower yields. Similar challenges may affect organosilicon derivatives, although such issues have not yet been reported. Table 3 provides a comprehensive summary of the precursors and methodologies employed for ²¹¹At‐astatination using [²¹¹At]At^+^.

**Table 3:** Summary of labeling characteristics for aromatic ^211^At‐astatinations using [^211^At]At^+^.

Precursor type	Labeling characteristics, key points
Direct astatination of arenes	Requires harsh conditions and elevated temperatures Potentially low selectivity
Aryl mercury compounds	Caution: highly toxic precursors Selectivity issues in installing the chloromercury group Generally high RCCs
Aryl thallium trifluoroacetates	Caution: highly toxic precursors Similar selectivity issues as aryl mercury compounds Generally high RCCs
Aryl trialkylstannanes	Toxic precursors labile to destannylation Mild reaction conditions Generally high RCCs
Aryl trialkylsilanes	Non‐toxic and highly stable Suitable for early‐stage incorporation of the trialkyl silyl group Requires harsh reaction conditions
Aryl trimethylgermanes	Trade‐off between reactivity, stability and toxicity of organotin and organosilicon compounds Requires harsher conditions than aryl trialkylstannanes
Aryl boronates	Non‐toxic precursors Generally milder conditions Extended reaction times and heating needed for complex ^211^At‐labeled PSMA‐inhibitors

### 6.3 ^211^At‐Labeled Synthon Strategies

Nucleophilic and electrophilic labeling strategies are important tools to develop new ^211^At‐based radiopharmaceuticals. However, direct labeling is often not feasible for certain molecules, as functional groups within the precursor can deactivate the reactive ^211^At species or may not tolerate the harsh labeling conditions. In such cases, labeling can be achieved using synthons—highly reactive intermediates that are astatinated and purified, often via HPLC, before being conjugated to the molecule of interest. This approach is commonly employed for labeling peptides, antibody fragments, mAbs, or proteins. A recent report by Vanermen et al. provides an extended overview of the available prosthetic groups [98]. A prominent example of such a synthon is [^211^At]SAB, which is used to label lysine residues. Over the years, numerous other synthon‐based labeling strategies have been developed and are thoroughly reviewed elsewhere [27, 151, 152]. A current challenge with these methods is the relatively low reactivity of the synthons after radiolabeling, which necessitates high precursor concentrations to achieve acceptable (but not quantitative) RCYs. This often requires additional purification steps to separate unreacted radioactive synthon from the radiolabeled product. In many cases, the inability to separate the radiolabeled product from the non‐labeled precursor further complicates clinical translation. Recently, a new class of ultra‐reactive, click‐chemistry based synthons has been developed, offering quantitative RCYs within 10–20 min. These reactions are notable for proceeding without need for precursor separation and can even be performed in aqueous conditions [153, 154]. Future research will confirm whether this chemistry can be universally applied across a broad range of targeting vectors.

## 7 Stability of the Carbon‐Astatine‐211 Bond

Several synthetic methods are available for forming carbon‐astatine bonds. However, a major limitation in the use of ^211^At‐labeled radiopharmaceuticals is in vivo dehalogenation. When ^211^At is released from the radiolabeled compounds, it accumulates in healthy tissues, particularly to the thyroid and stomach, leading to off‐target toxicity. This challenge is reflected in the BDEs of phenyl‐ and alkyl‐halogen bonds (Table 4) [18]. Astatine bonding is predominantly confined to sp^2^‐hybridized carbons rather than sp^3^‐hybridized once, as the BDE of astatine‐carbon bonds in sp^3^‐hybridized are too low for biomedical applications [27, 115]. Additionally, it is important to note that astatine is the largest halogen, with an atomic radius comparable to that of a phenyl ring (Table 4) [20]. This large atomic size may influence the incorporation of ^211^At into radiopharmaceuticals and potentially affect their targeting properties.

**Table 4:** Atomic radii of halogens, size comparison with a phenyl ring, and bond dissociations energies of phenyl‐ and alkyl‐halogen bonds [20, 27].

Halogen	Radius [pm]	Halogenated phenyl, actual size	Phenyl‐Halogen [kcal/mol]	Alkyl‐Halogen [kcal/mol]
F	67	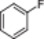	125	106
Cl	99	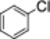	95	81
Br	114	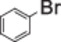	80	68
I	133	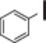	64	53
At	145	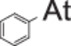	47	39

**Figure 15:**
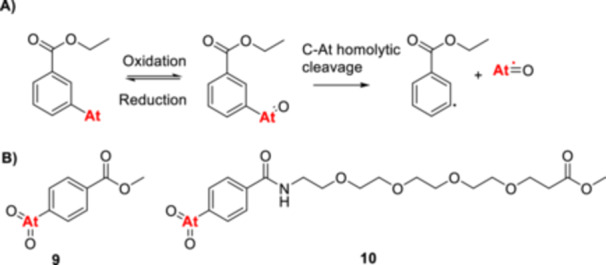
(A) Proposed mechanism for deastatination. Oxidation of ^211^At to a +III species renders the carbon‐astatine bond susceptible to homolytic cleavage [19, 148]. (B) Pre‐oxidized compounds hypothesized to exhibit greater resistance to further in vivo oxidation [149].

The uptake of free ^211^At in the thyroid and stomach is comparable to that of free iodine [27, 155]. However, unlike iodine, free ^211^At also accumulates in the spleen and lungs. This may be attributed to the in vivo oxidation of At^−^ to At^+^, which facilitates its distribution to these additional organs [27]. To illustrate the challenge posed by the lower in vivo stability of the carbon‐astatine bond compared to the carbon‐iodine bond, the in vivo stability of iodinated versus astatinated benzoate derivatives has been evaluated. While iodinated derivatives exhibit good in vivo stability, their astatinated counterparts undergo significant deastatination [90, 148]. To mitigate the accumulation of unbound astatine, blocking agents such as potassium‐iodide or liothyronine sodium can been administered before the astatinated drug to pre‐saturate the thyroid. This iodine‐blocking strategy is already well established in clinical practice before the administration of meta‐[^131^I]I‐iodobenzylguanidine ([^131^I]**MIBG**) and [^123^I]**MIBG,** theranostics radiopharmaceutical for neuroblastoma treatment [156]. In a preclinical study, Watabe et at. demonstrated that pre‐administration of sodium iodine in normal male ICR mice reduced thyroid uptake of free ^211^At (presumably resulting from tracer deastatination) by 81% [157]. While the use of blocking agents can help reduce off‐target accumulation, it does not address the core challenge—preventing in vivo deastatination.

The chemical and biological mechanisms of deastatination remain unknown. Proposed explanations include the lower dissociation energy of the astatine‐carbon bond compared to the iodine‐carbon bond (Table 4) as well as potential action of unidentified enzymes [148]. Given that astatine is the rarest naturally occurring element, the existence of astatine‐specific enzymes is unlikely. However, enzymes involved in iodine metabolism might also show affinity for corresponding ^211^At‐derivatives. For example, the sodium iodide symporter is known to recognize both iodine and astatine [148, 158, 159]. Additionally, cytochromes P‐450 (CYPs) have been shown to catalyze the oxidation of iodobenzene into iodosobenzene. Since heavier halogens are more susceptible to oxidation, these CYPs may also contribute to the oxidation of ^211^At‐labeled radiopharmaceuticals [148, 160].

The proposed BDE of astatobenzene and iodobenzene are 44.9 ± 5.1 and 61.1 ± 4.7 kcal/mol, respectively, highlighting a notable difference in stability [148]. However, these values do not explain why [^211^At]astatobenzoate‐labeled proteins are relatively stable in blood but not in cell‐based assays [148]. For example, the model compound, 3‐[^211^At]‐ethylastatobenzoate exhibited significantly release of free ^211^At under Fenton‐like oxidative conditions, in which trivalent ferric iron catalyzes reactions with hydrogen peroxide. These conditions mimic the oxidative environment found in lysosomes [148]. In contrast, no substantial deiodination was observed in 3‐ethyliodobenzoate under the same conditions. This suggests that oxidation plays a crucial role in the deastatination process, potentially via the mechanism shown in Figure 15A. In this proposed pathway, phenyl‐bound ^211^At is first oxidized to the +III oxidation state, followed by homolytic cleavage of the carbon‐halogen bond. The lower BDE of the carbon‐astatine bond (28.2 kcal/mol) compared to the carbon‐iodine bond (37.8 kcal/mol) facilitates this cleavage [19, 148]. In fact, the dissociation rate of the astatine compound is 6 × 10⁶ times higher at 37°C [148]. This supports the observed trend: while carbon‐iodine bonds are more stable, the carbon‐astatine bond cleaves more readily, which could explain the release of ^211^At in lysosomes where reactive oxygen species are present [148].

As previously discussed, in vivo oxidation of ^211^At‐astatoarene compounds may lead to ^211^At‐deastatination. To explore this further, Li et al. [149] compared the in vivo stability of ^211^At‐astatoxyarenes with their corresponding ^125^I‐iodoxy derivatives, in which the radiohalogens were pre‐oxidized to the +V oxidation state [149]. Oxidized forms of *p*‐[^125^I]iodoxybenzoic acid methyl ester and *p*‐[^211^At]astatoxybenzoic acid (**9**) methyl ester were synthesized (Figure 15B) [149]. While ^125^I‐iodoxy derivatives remained stable to in vivo deiodination, the ^211^At‐astatoxy derivative showed significant decomposition. Notably, biodistribution profile of the astatinated derivative closely resembled that of [^211^At]NaAtO_3_. This finding suggests that the compounded to form AtO_3_^−^, indicating that both oxidation and deastatination occurred in vivo [149].

### 7.1 Strategies to Reduce Deastatination In Vivo

Over the years, several strategies have been developed to minimize or prevent deastatination. One strategy leverages the properties of charged species to reduce exocytosis of radiolabeled catabolites [130]. Specifically, Vaidyanathan et al. demonstrated that guanidinomethyl functionalization enhances the stability of astatinated compounds [161, 162]. Another strategy involves incorporating the neopentyl glycol scaffold which highlights the critical role of hydroxyl groups in stabilizing compounds against CYP‐mediated metabolism [90]. Additionally, stable boron cage derivatives have been developed, in which the carbon‐astatine bond is replaced by a stronger boron‐astatine bond [163]. In the following, we will discuss these strategies in detail, along with the underlying principles aimed at further reducing deastatination.

#### 7.1.1 Guanidinomethyl Functionalization

**Figure 16:**
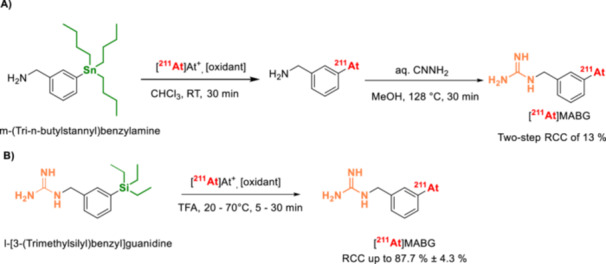
Synthesis of [^211^At]MABG from (A) organotin or (B) silicium precursors.

Building on the success of the FDA‐approved radiopharmaceutical [^131^I]**MIBG** for treating neuroblastoma, Vaidyanathan et al. [141] developed an astatinated derivative, meta‐[^211^At]At‐astatobenzylguanidine ([^211^At]**MABG**). The initial synthesis involved a two‐step process, first astatination of 3‐(tri‐n‐butylstannyl)benzylamine, followed by formation of the guanidinium moiety (Figure 16) [141, 151]. To facilitate purification from the organotin precursor, a kit‐based approach was utilized, anchoring the tin precursor to a solid support. This method achieved a RCY of 63 ± 9% [164]. The procedure was later streamlined using a 1‐[3‐(trimethylsilyl)]‐benzylguanidine precursor in a one‐step synthesis. Under optimized conditions—labeling at 70°C in TFA with NCS—this method yielded a RCC of 88 ± 4% (Figure 16) [141].

The initial rationale for incorporating a guanidinomethyl prosthetic group is to minimize oxidative decomposition within lysosomes following cellular internalization. Charged catabolites, such as protonated guanidine (pKa ≈ 13), are less likely to undergo exocytosis in the acidic lysosomal environment, as they cannot efficiently cross the lysosomal membrane [165, 166]. Thus, guanidinomethyl functionalization in ^211^At‐labeled radiopharmaceuticals may trap ^211^At inside cells after vector degradation, reducing off‐target accumulation [129]. This mechanism, however, requires prior internalization of the radiolabeled compound. Additionally, the guanidinomethyl group may enhance stability by providing steric hindrance against deastatination [66].

**Figure 17:**
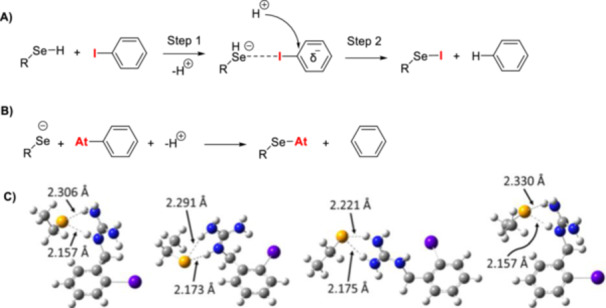
(A) Mechanism for deiodination of iodoaryl substrates by deiodinase enzymes. (B) The same selenocysteine‐mediated mechanism can also apply for astatobenzene. (C) Density function theory and molecular modeling show how guanidinomethyl functionalization hinders deastatination by through charge‐charge interactions between the selenocysteine in the enzyme active site and the guanidinium. **Color code**: White = hydrogen, grey = carbon, blue = nitrogen, orange = selenium, purple = astatine. The figure is reprinted with permission from Yssartier et al. *RSC Med Chem*. 2024, *15*, 223. Copyright (2024) The Royal Society of Chemistry.

Yssartier et al. [89] recently proposed an alternative mechanism for the guanidinium group's stabilizing effect, inspired by the deiodination of iodoaryl substrates by deiodinase enzymes. Specifically, Types 1 and 3 iodothyronine deiodinases catalyze the reductive elimination of phenyl‐bound iodine from thyroid hormones (Figure 17A) [167]. In this mechanism, selenocysteine residues in the enzyme's catalytic site interacts with the iodine atom through a halogen bond interaction. This interaction weakens and elongates the carbon‐iodine bond by inducing an Umpolung effect, making the carbon atom nucleophilic. As a result, electrophilic aromatic substitution occurs with a surrounding proton, leading to the formation of a covalent selenium‐iodine bond and the deiodinated arene [89]. Extending this model, the authors demonstrated that selenocysteine similarly mediates the deastatination of [^211^At]astatobenzene (Figure 17B) [89].

Density functional theory (DFT) calculations revealed that the carbon‐astatine bond dissociation enthalpy for both [²¹¹At]astatobenzene and 1‐(o‐[²¹¹At]astatobenzyl)guanidine was 47 kcal/mol, indicating that the guanidinium group does not significantly affect the strength of the carbon‐astatine bond. However, the introduction of the guanidinomethyl group resulted in an interaction energy difference of approximately 81 kcal/mol between the selenocysteine complexed with 1‐(o‐[²¹¹At]astatobenzyl)guanidine and [²¹¹At]astatobenzene [89]. The increased stability of 1‐(o‐[^211^At]astatobenzyl)guanidine was attributed to strong electrostatic interactions between the positively charged guanidinium group and the negatively charged selenocysteine. As illustrated in Figure 17C, hydrogen bonding between hydrogen atoms of the guanidinium moiety and the negatively charged selenium is dominated by the charge‐charge interaction. These interactions effectively prevent further interactions between astatine and selenocysteine [89]. This mechanism explains how the guanidinium group stabilizes the radiopharmaceutical by reducing deastatination.

**Figure 18:**
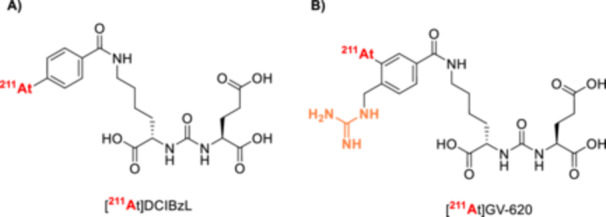
Astatinated Glu‐urea based PSMA ligands. (A) Unmodified astatoarene and (B) guanidinomethyl‐functionalized astatoarene [162].

Guanidinomethyl functionalization has been shown to enhance the stability of several ²¹¹At‐labeled compounds, including inhibitors of PSMA [162], anti‐HER2 nanobodies [137], and anti‐HER2 5F7 single‐domain antibody fragments [161]. One study by Vaidyanathan et al. [162] focused on improving the in vivo stability of [²¹¹At]**DCABzL**, a PSMA‐targeting ligand (Figure 18A) [126, 162]. The lead candidate, guanidinomethyl‐functionalized [^211^At]**GV‐620** (Figure 18B), demonstrated reduced accumulation in non‐target tissues. while thyroid uptake of [²¹¹At]**DCABzL** and [²¹¹At]**GV‐620** was comparable (0.62 ± 0.23 and 0.77 ± 0.25 %ID/g, respectively, 2 h after injection), a notable difference was observed in the stomach: [²¹¹At]**GV‐620** showed markedly lower accumulation (2.55 ± 0.69 %ID/g) compared to [²¹¹At]**DCABzL** (10.09 ± 1.66 %ID/g) [126, 168]. When compared to [^131^I]**GV‐620**, [^211^At]**GV‐620** showed higher uptake in the thyroid, stomach, lungs, heart, and intestines, suggesting partial deastatination [162]. These findings indicate that guanidinomethyl functionalization contributes to reduced non‐target tissue accumulation and improved stability of the [²¹¹At]‐labeled compound.

**Figure 19:**
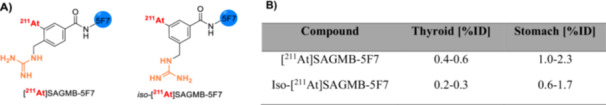
(A) Guanidinomethyl‐functionalized motifs conjugated to anti‐HER2 5F7 single‐domain antibody fragments, with the guanidinium group positioned either ortho or meta to ^211^At. (B) Thyroid and stomach accumulation following injection in SCID mice bearing subcutaneous BT474M1 breast carcinoma xenografts. Timepoints include 1, 2, 4, and 21 h post‐injection. %ID = percentage injected dose [161].

Anti‐HER2 5F7 single‐domain antibody fragments have been modified to produce [^211^At]**SAGMB‐5F7** and *iso*‐[^211^At]**SAGMB‐5F7** (Figure 19A). Biodistribution studies showed significantly higher tumor uptake for *iso*‐[^211^At]**SAGMB‐5F7** (23.4 ± 2.2 %ID/g) compared to [^211^At]**SAGMB‐5F7** (15.7 ± 1–7 %ID/g) [161]. Iso‐[²¹¹At]**SAGMB‐5F7** also showed comparatively lower thyroid and stomach uptake resulting in improved tumor‐to‐normal organ ratios. (Figure 19B). These findings supports its potential as a candidate for therapeutic applications [161]. The enhanced performance of iso‐[²¹¹At]**SAGMB‐5F7** can be attributed to the greater spatial separation between the guanidinomethyl substituent and the ²¹¹At‐labeled moiety in the meta‐substituted iso‐isomer compared to the ortho‐regioisomer. Notably, iso‐[²¹¹At]**SAGMB‐5F7** demonstrated higher HER2 binding affinity in BT474M1 human breast carcinoma cells, further supporting its therapeutic potential [161]. Another example is NB7, a single domain antibody fragment, with high affinity for an epitope on PSMA. Using His_6_‐tagged NB7, [^211^At]**SAGMB‐NB7H6** was synthesized [169]. Its thyroid and stomach accumulation were comparable to iso‐[²¹¹At]**SAGMB‐5F7**. Interestingly, in this study, the iodine analog [^125^I]**SGMIB‐NB7H6** outperformed iso‐[^125^I]**SGMIB‐NB7H6,** suggesting that the optimal orientation of the guanidinomethyl is compound‐specific [169].

#### 7.1.2 The Neopentyl Glycol Scaffold

**Figure 20:**
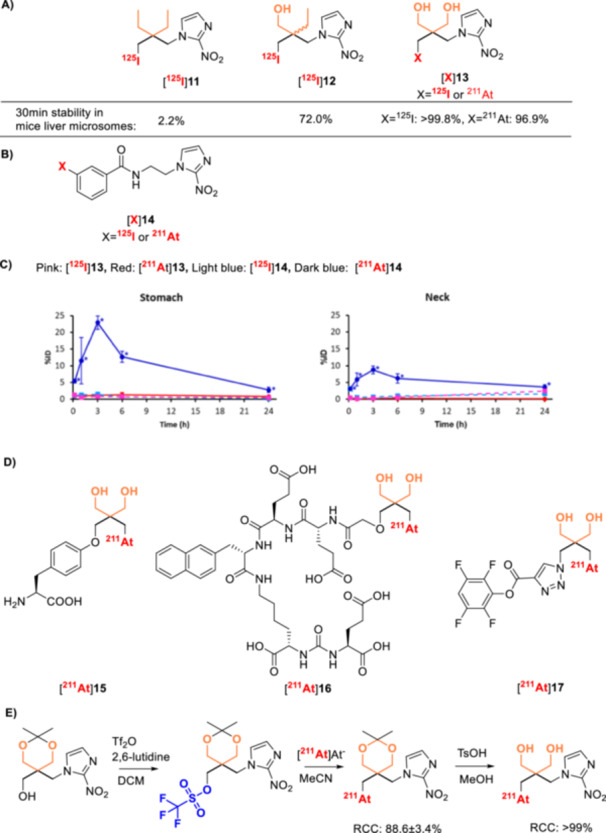
(A) Compounds synthesized to evaluate the stabilizing properties of the hydroxyl groups in the neopentyl glycol scaffold, and (B) benzoate reference compound. (C) Accumulation of ^125^I and ^211^At in the stomach and neck, respectively. Reprinted with permission from Suzuki et al. *J. Med. Chem*., 2021, 64, 15846‐15857. Copyright 2014 American Chemical Society. (D) Additional applications of the neopentyl glycol scaffold including an l‐tyrosine derivative (**15**), a PSMA‐targeting derivative (**16**), and an activated ester for biomolecule conjugation (**17**). (E) Synthesis, astatination, and deprotection of the neopentyl glycol precursor. The triflate precursor is prepared from the corresponding alcohol by trifluoromethanesulfonic anhydride (Tf_2_O) and 2,5‐lutidine in DCM. Astatination is performed with astatide in acetonitrile. Deprotection is achieved using p‐toluenesulfonic acid (TsOH) in methanol.

The neopentyl glycol scaffold has emerged as a promising strategy for stabilizing aliphatic ^211^At derivatives. Inspired by the high in vivo stability of 2,2‐dihydroxymethyl‐3‐[^18^F]‐fluoropropyl‐2‐nitroimidozole [170], Suzuki et al. investigated whether ^125^I‐ and ^211^At‐derivatives would also exhibit increased in vivo stability [171]. A significant correlation was observed between the presence and number of hydroxyl groups and the stability of the corresponding ^125^I‐neopentyl glycol derivatives against nucleophilic substitution and CYP‐mediated metabolism (Figure 20A). Radio‐HPLC and ‐TLC analyses revealed that the derivatives [^125^I]**11,** [^125^I]**12,** and [^125^I]**13** exhibited 2.2%, 72.0%, and > 99.8% stability in mouse liver microsomes after 30 min, respectively [90]. The authors concluded that the hydroxyl‐groups of the neopentyl glycol scaffold may prevent CYP‐mediated dehalogenation through steric hindrance or increased hydrophilicity, thereby impairing CYP recognition [90, 172]. In vivo, the major metabolites of both the ^125^I‐ and ^211^At‐labeled derivatives were glucuronide conjugates, suggesting that the hydroxyl groups in the scaffold could enhance radiopharmaceutical clearance from the bloodstream [173]. Compared to benzoate derivatives (Figure 20B), the neopentyl glycol analogs demonstrated similar chemical and biological properties but significantly reduced accumulation of free ^211^At in the stomach and neck, indicating lower deastatination (Figure 20C) [90].

To broaden the scope of the neopentyl glycol scaffold, Kaizuka et al. [172] developed [^211^At]**15**, a neopentyl glycol l‐tyrosine derivative, targeting the l‐type amino acid transporter (Figure 20D). The compound showed high stability in PBS and fetal bovine serum over a 3‐h period, as well as high tumor uptake in C6 glioma tumor‐bearing immunodeficient nude mice, though with only moderate retention. Notably, [^211^At]**15** appeared to be primarily excreted through the amino acid transporter rather than being incorporated into protein synthesis, with notable concentrations in the kidney and pancreas, followed by rapid excretion. Accumulation in the liver and intestines was observed, likely due to the lipophilic nature of the compound, potentially affecting its pharmacokinetic profile [172, 174].

The ^211^At‐labeled neopentyl glycol scaffold has also been incorporated into PSMA‐targeting vectors to enhance in vivo stability. Its compatibility with peptide coupling conditions and the ability to cleave acid‐sensitive protection groups facilitates its integration into peptides. In a study by Suzuki et al. [91], the ^211^At‐labeled PSMA derivative ([^211^At]**16**) demonstrated low accumulation in the stomach (1.74 ± 0.39 %ID/g) and thyroid (0.55 ± 0.33 %ID/g) in tumor‐bearing mice 3 h postinjection. Notably, it exhibited high tumor accumulation (16.9 ± 8.45 %ID/g), indicating effective tumor targeting and in vivo stability. These findings were verified by Yaginuma et al. [175], who observed [^211^At]**16** tumor uptake of 42.0 ± 13.1 %ID/g after 3 h with minimal uptake in thyroid, stomach, and salivary glands (0.28 ± 0.20 %ID/g, 0.71 ± 0.12 %ID/g and 0.88 ± 0.10 %ID/g, respectively) in BALB/c nu/nu mice subcutaneously transplanted with PSMA‐positive PC‐3 PIP cells. The antitumor effect of [^211^At]**16** was dose‐dependent, with tumor volume increases of 161.0%, –76.4%, and –59.5% at 0.32, 1.00, and 1.93 MBq doses, respectively, compared to a 796.0% increase in the saline‐treated control group by Day 15. Mild but reversible renal damage was observed at 1.00 MBq doses, while irreversible renal damage occurred after administering 1.93 MBq [175]. More recently, the copper‐catalyzed azide‐alkyne cycloaddition was applied to conjugation the neopentyl glycol scaffold with an α‐melanocyte stimulating hormone peptide analog [176]. Incorporation of a hydrophilic d‐Glu‐d‐Arg linker, resulted in favorable biodistribution in B16F10 tumor‐bearing mice, with minor thyroid uptake. Furthermore, high therapeutic efficacy was demonstrated by inhibited tumor growth following administration of both 0.4 and 1 MBq doses [176].

^211^At is typically introduced into the acetal‐protected neopentyl glycol scaffold through nucleophilic substitution of sulfonyl ester derivatives at room temperature over a 5‐min period. Subsequent hydrolysis of the acetal protection group quantitatively yields in the deprotected ^211^At‐labeled neopentyl (Figure 20E) [90]. Although the labeling conditions have not been fully optimized, [^211^At]**15** was synthesized with a RCY of 44% [172]. More recently, [^211^At]**17**, an active ester derivative, was conjugated to cetuximab, achieving a RCY of 27 ± 1% at 200 kBq scale (Figure 20D) [171]. Unreacted activated ester was hydrolyzed to the corresponding carboxylic acid. These findings highlight the potential of the neopentyl glycol scaffold for use in radiopharmaceuticals, particularly for targeted therapies involving both small molecules and antibodies.

#### 7.1.3 Boron Clusters

Efforts to improve the stability of astatinated compounds have increasingly focused on exploring stronger bonds, particularly involving boron. This shift is driven by the significantly higher BDE of boron‐astatine bonds (~79 kcal/mol) compared to carbon‐astatine bonds (~43 kcal/mol) [31]. This trend aligns with the general pattern of boron‐halogen bonds being stronger than their carbon counterparts, as seen with boron‐iodine (91 kcal/mol) versus carbon‐iodine bonds (53 kcal/mol) [27]. The distinct polarization of these bonds further underscores the difference between carbon‐astatine and boron‐astatine interactions. In boron‐astatine bonds, astatine carries a negative polarization due to boron's lower electronegativity (*χ* = 4.29 eV) relative to astatine (*χ* = 5.87 eV, Mulliken scale) [31, 33]. Conversely, in carbon‐astatine bonds, astatine is positive polarization because carbon has a higher electronegativity (*χ* = 6.27 eV). This reversal in polarization profoundly influences the chemical reactivity and degradation pathways of these bonds. Boron‐bound astatine is more susceptible to electrophilic attacks due to its negative polarization, whereas carbon‐bound astatine is vulnerable to nucleophilic attack, These polarization‐driven vulnerabilities also affect to the bonds’ resistance to reductive or oxidative cleavage and influence how enzymes recognize and catalyze deastatination [31, 33, 163].

**Figure 21:**
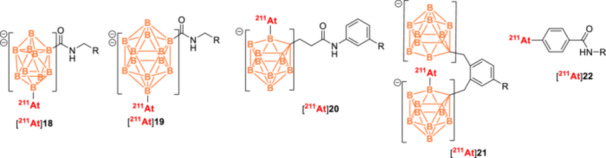
Boron clusters studies for their vivo stabilization of astatine‐211. R can be linkers to targeting vectors.

Both ^211^At‐astato‐*nido*‐carborate and ^211^At‐astato‐*closo*‐decarborate moieties have been studies for their in vivo stability (Figure 21). Labeling succeeds in PBS by reacting the boron cluster with [^211^At]NaAt in the presence of aqueous chloramine‐T as an oxidizing agent. The reaction proceeds within 30 s to 2 min, after which aqueous sodium pyrosulfite (Na_2_S_2_O_5_) is added to quench the reaction. The reaction mixture is then purified using a NAP‐10 column [163, 177]. Under non‐optimized conditions, labeling yields for antibody Fab’ fragment conjugates range from 28% to 75%, depending on the specific protocol and reaction parameters [163].

Studies comparing ^125^I‐ and ^211^At‐labeled *closo*‐decarborate(2‐) moiety [^211^At]**18**, conjugated to the anti‐PSMA antibody Fab fragment (107‐1A4), revealed notable differences in biodistribution in BALB/c nu/nu mice. Higher ^125^I uptake was observed in the neck and stomach compared to the ^211^At‐labeled analog, indicating highest susceptibility to deiodination [24, 163]. Further comparison of [^211^At]**18** with astatoaryl compound [^211^At]**22** in male athymic mice (*nu/nu*) showed that the boron cluster exhibited reduced uptake in the thyroid, stomach, and lungs but increased and prolonged retention in the blood and liver [19, 163]. These extended retention times are likely attributable to the intrinsic properties of the boron cluster structure.

In studies involving ^211^At‐astato‐*nido*‐carbonyl derivatives [^211^At]**20** and [^211^At]**21**, aggregation of the Fab’ fragment upon conjugation led to altered in vivo behavior, notably increased blood and liver retention [163]. In a comparison study, the *closo*‐decaborate(2‐) moiety [^211^At]**18** outperformed the *closo*‐dodecaborate(2‐) moiety [^211^At]**19**, displaying faster tissue clearance and lower kidney uptake [178]. These characteristics make [^211^At]**18** a more promising candidate for radiopharmaceutical development. To reduce kidney retention, an acid‐labile hydrazone linker was introduced between the boron cluster and Fab fragment in **18** conjugates [177]. In ^125^I‐labeled conjugates, clearance from kidneys, liver, and spleen were observed. In contrast, ^211^At‐labeled conjugates showed no kidney clearance, but significantly higher tumor uptake (42.28 ± 16.38 %ID/g) compared to the ^125^I‐derivative (13.14 ± 2.03 %ID/g) at 4 h post‐injection in nude mice bearing LNCaP human tumor xenografts [177].

The bis‐*nido*‐carboranyl derivative [^211^At]**21** demonstrated greater stability than the mono‐*nido*‐carboranyl derivative [^211^At]**20**. This enhanced stability is hypothesized to arise from a halogen bond interaction between the astatine atom and the second negatively charged *nido*‐carborane group, which acts as a Lewis base. This interaction effectively bridges the two *nido*‐carborane groups via ^211^At [89]. Overall, ^211^At‐labeled boron cages exhibit good resistance to in vivo ^211^At‐deastatination. Nevertheless, their undesirable biodistribution profiles highlights the need for further pharmacokinetic optimization to improve their therapeutic potential [90].

#### 7.1.4 Complexation

Given astatine's metalloid nature, research has explored its metallic character through complexation with various chelating agents. In 1988, Milesz et al. [179] demonstrated the complexation of electrophilic [^211^At]At^+^ with ethylenediaminetetraacetic acid (EDTA) [179]. The following year, the same group reported chelation of [^211^At]At^+^ with diethylenetriaminepentaacetic acid (DTPA) and its subsequent conjugation to a polyclonal IgG antibody [180]. However, biodistribution studies of the [^211^At]‐DTPA‐antibody conjugate revealed organ accumulation patterns similar to those observed for free [^211^At]At^−^, indicating low complex stability [181]. Subsequently, Yordanov et al. [182] reported the formation of a [^211^At]‐callix[4]arene complex and assessed its stability in vivo. Despite its innovative chelate design, the complex displayed a biodistribution in nude mice similar to that of free [^211^At]At^−^, underscoring persistent challenges in achieving robust chelation of astatine for radiopharmaceutical applications [182, 183]. Complexation of [^211^At]At^+^ with nitrilotriacetic acid (NTA) was also investigated. The resulting complex was stable under oxidative conditions within a pH range of 4‐8, but degraded in more basic solutions [184]. Additional studies explored chelation of astatine in higher oxidation states using macrocyclic chelators such as DOTA and NOTA [185]. While complex formation was suggested, the resulting [^211^At]‐DOTA and [^211^At]‐NOTA complexes proved unstable, highlighting the need for improved strategies. Recent work has reported the chelation of AtO^+^ by ketones in the presence of nitrate (NO_3_^−^) [186], identifying this interaction as the underlying mechanism for the liquid‐liquid extraction of ^211^At from 6 M HNO_3_ into ketone‐based solvents.

In its reduced form ([^211^At]At^−^), astatine behaves as a soft Lewis base. This has prompted research into forming stable complexes/bonds with soft Lewis acids. A similar strategy has been applied for successful chelation of [^18^F]AlF using NOTA [187]. Pruszyński et al. [188] were the first to report complex formation betwen [^211^At]At^−^ with a soft Lewis acid. Their study compared the complexation of [^131^I]I^−^ and [^211^At]At^−^ with mercury(II) hydroxide (Hg(OH)_2_), and using electromigration, stronger binding for ^211^At was revealed, supporting its potential for such approaches [188].

**Figure 22:**
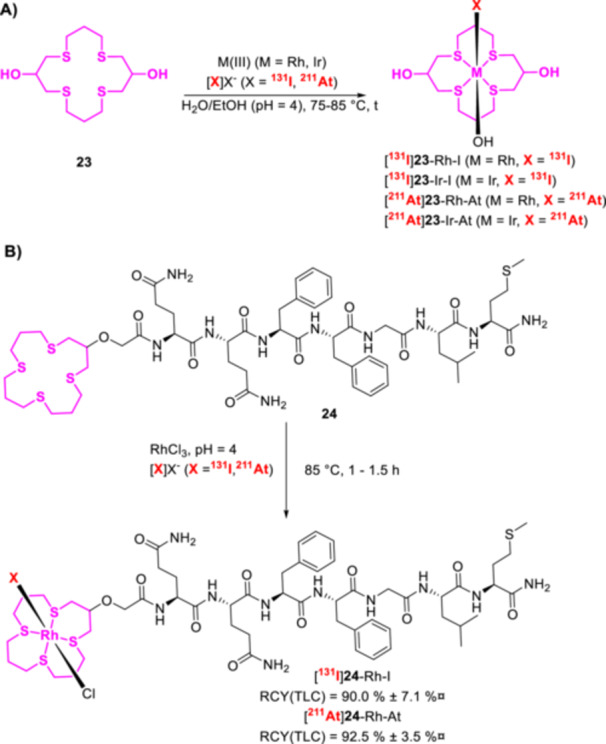
(A) Radiosynthesis of M(III)‐^131^I/^211^At‐complexes (M = Rh(III) and Ir(III)), using the chelator 16aneS_4_‐diol. (B) Direct radiosynthesis of ^131^I‐ and ^211^At‐labeled substance P (5‐11) ([^131^I]**24**‐Rh‐I, [^211^At]**24**‐Rh‐At).

Later, the same group studied complexation of [^211^At]At^−^ with Rh(III) and Ir(III), both chelated by the macrocyclic thioether 1,5,9,13‐tetrathiacyclohexadecane‐3,11‐diol (16aneS_4_‐diol) (Figure 22A) [189]. The resulting complexes ([^131^I]**23**‐Rh‐I, [^131^I]**23**‐Ir‐I, [^211^At]**23**‐Rh‐At, [^211^I]**23**‐Ir‐At) were obtained in high RCYs of approx. 80%–90%. Reactions were performed using 62.5 nmol of Rh(III) or Ir(III) source, 250 nmol of 16aneS4‐diol, at pH 4 and 75°C–85°C for 1–1.5 h [189]. Further studies demonstrated the stability of [^211^At]**23**‐Rh‐At in PBS and human serum at both 25°C and 37°C [190]. Biodistribution studies in BALB/c mice revealed higher uptake in the spleen, lungs, and stomach at 30 min post‐injection, with significant clearance over the following 4 h. Compared to free [^211^At]At^−^, the %ID/g in these tissues was notably lower for [^211^At]**23**‐Rh‐At [183, 190]. This complex was subsequently used to label substance P with ^211^At for glioblastoma treatment (Figure 22B) [191]. Two labeling procedures were developed: (1) conjugating substance P to the preformed complex, and (2) directly astatinate substance P pre‐conjugated to 16aneS4‐diol. The latter method yielded higher RCYs in shorter reaction times (Figure 22B). The resulting ^211^At‐labeled substance P derivative ([^211^At]**24**‐Rh‐At) was stable in PBS and cerebrospinal fluid, as well as demonstrated superior efficacy in treating human glioma T98G cells, compared to free [^211^At]At^−^ at activity concentrations as low as 75 kBq/mL [191]. In vivo data for this compound is missing.

While the metallic character of ^211^At offers opportunities for complexation with various chelators, limited stability—particularly in vivo—has hindered the broader application in radiopharmaceutical development. However, the complexation of [^211^At]At^−^ with soft Lewis acids shows promise for creating more stable complexes suitable for in vivo use.

#### 7.1.5 Astatinated Gold Nanoparticles

Gold nanoparticles (AuNPs) are highly valued in biomedicine due to their outstanding chemical stability and biocompatibility. Progress in the field enabled production of monodispersed NPs in various sizes and shapes [192]. One widely used synthetic strategies is aqueous reduction of tetrachloroauric acid (HAuCl_4_) by sodium citrate, which acts both as a reducing and capping agent. This dual role allows control over NP size, as higher citrate concentration yield smaller NPs and vice versa [192, 193].

The development of ^211^At‐labeled AuNPs holds significant promise for targeted radiotherapy. In this approach, astatine is adsorbed onto the surface of gold, similar to the adsorption of (radio)iodine. Both astatine and iodine preferentially adsorb onto the face‐centered cubic (fcc) hollow sites and the edge‐bridge sites of the gold surface. Density of states analysis show that the 5 d orbitals of gold hybridize with the 6p‐and s‐ orbitals of ^211^At [193]. One advantage of this method is the ability to load multiple radionuclide atoms onto a single nanoparticle, enabling the delivery of higher radioactive doses [193]. Additionally, AuNPs can be functionalized with proteins or ligands for targeted delivery [193, 194]. Another major benefit is the simplicity of the labeling procedure: ^211^At is stirred in an aqueous solution containing the AuNPs, typically at room temperature for 5–20 min [194, 195].

In 2017, Dziawer et al. [196] developed ^211^At‐labeled AuNPs functionalized with substance P peptide fragments for glioma‐targeting applications. Using 5 and 15 nm AuNPs, the constructs demonstrated high stability in human serum and cerebrospinal fluid, along with in vitro cytotoxicity against glioma cells, providing strong proof of concept for this approach [196]. To improve biocompatibility, Sporer et al. [195] described the adsorption of ^211^At onto PEGylated AuNPs (25–50 nm), which exhibited > 95% stability in serum after 4 h. In vivo biodistribution studies revealed typical NP behavior, including prolonged circulation and significant liver and spleen uptake. Importantly, low accumulation of ^211^At in the thyroid and stomach indicated robust particle stability [195]. Huang et al. further demonstrated the tumor‐ targeting potential of ^211^At‐labeled AuNPs, showing that smaller particles achieved greater tumor accumulation. Specifically, 5 nm ^211^At‐AuNPs displayed higher tumor uptake (2.25 ± 0.67 %ID/g) than 30 nm particles (1.29 ± 0.17 %ID/g) at 3 h post‐injection in PANC‐1 tumor‐bearing xenograft mice. A dose of 0.5 MBq per mouse with 5 nm particles was sufficient to suppress tumor growth. However, modifying AuNPs with the H16 peptide—designed to target the acidic tumor microenvironment—did not enhance tumor uptake and instead increased liver accumulation [197]. Similarly, ^211^At‐labeled “gold nanostars” exhibited promising features, including low thyroid and stomach uptake and significantly tumor suppressive following intratumorally injection in a U87MG human glioma xenograft murine model. These constructs were also stable in vitro, maintaining > 99% integrity in human serum at 37°C over 24 h [198]. While the successful adsorption of ^211^At onto AuNPs has been well‐documented, data on how varying ^211^At concentrations affects adsorption strength and radionuclide stability remain limited, an important consideration for clinical optimization [193].

Beyond AuNPs, silver and polymeric micelles have also been evaluated as ^211^At carriers. However, due to unfavorable in vivo and in vitro properties, neither has progressed to further preclinical development [199, 200]. Hou et al. [201] synthesized astatinated folic acid‐functionalized silver nanoparticles (~10 nm) via a one‐pot assembly with SH‐PEG‐FA, achieving > 95% RCY within 15 min. In 4T1 tumor‐bearing mice, tumor uptake was 2.8 ± 0.8 %ID/g at 12 h after V intertumoral administration, while liver uptake reached 20.8 ± 13.7 %ID/g—likely due to macrophage‐mediated clearance. Thyroid uptake remained low (1.5 ± 1.3 %ID/g) [201]. These results highlight the need for improved biosafety before clinical translation of silver nanocarriers. Denk et al. [202] introduced a modular and versatile copper‐click‐based approach to synthesize multifunctional ^211^At‐labeled reagents. This method enables rapid radiolabeling and cross‐linking, offering a platform to produce sterically shielded ^211^At compounds. The resulting constructs were stable with less than 1% degradation or deastatination after 5 h in human plasma. This strategy offers a promising alternative to AuNP‐based systems, enabling the radiolabeling and subsequent surface modification of organic nanomaterials [202].

#### 7.1.6 Other Strategies to Minimize Deastatination

While the side effects of deastatination are well recognized, recent studies have reported low deastatination in certain radiopharmaceuticals, even in the absence of specific stabilization strategies. For example, Echigo et al. [203] developed a ^211^At‐SAB derivative conjugated to a dual‐function targeting vector combining an albumin‐binding moiety with an RGD peptide. This design aimed to enhance pharmacokinetics by prolonging circulation and improving tumor accumulation and retention. The ^211^At‐labeled compound outperformed its ^67^Ga‐counterpart in these metrics. However, extended blood retention led to increased off‐target accumulation in organs such as the lungs and heart. As a result, the compound was deemed suboptimal for RLT, though the authors suggested modifying the albumin‐binding moiety to reduce affinity and thereby circulation time. Despite this, minimal uptake in the stomach and thyroid indicated low deastatination [203], likely due to extended blood retention, as the compound avoids lysosomal oxidative conditions and enzymatic metabolism. Similarly, Mease et al. [125] reported an improved ^211^At‐labeled PSMA derivative ([^211^At]**7**‐Lu, Figure 12), demonstrating low deastatination in PSMA‐positive PC3 PIP tumor‐bearing NSG mice. One‐hour post‐injection, uptake in the stomach and salivary glands was 0.39 ± 0.12 %ID/g and 0.47 ± 0.19 %ID/g, respectively. At 24 h, the compound was nearly undetectable in normal organs. [^211^At]**7**‐Lu significantly increased median survival across all tested doses (0.24–3.7 MBq), attributed to the stability of the Lys‐Glu‐urea‐based scaffold, and the pharmacokinetic benefits of DOTA‐chelated Lutetium‐175 [125]. Building on this, [^211^At]**8**‐Ga (Figure 13) [135] was designed to further minimize deastatination by positioning ^211^At deep within the receptor‐binding pocket of the ligand, thus shielding it from enzymatic or oxidative degradation upon ligand binding. This rational design resulted in virtually no detectable deastatination, highlighting its therapeutic promise. Recent publications also suggest that specific substituents around the astato group can enhance the in vivo stability of the carbon‐astatine‐bond. Hirata et al. [204] introduced two ortho hydroxymethyl or dimethylcarbamoyl substituents, both of which reduced thyroid and stomach uptake compared to unsubstituted control. While the hydroxymethyl substituted astatinated compound showed higher levels of free halogen than its ^125^I analog, the di‐ortho dimethylcarbamoyl‐substituted compound showed comparable levels. The authors hypothesize that the electron‐withdrawing effect of the dimethylcarbamoyl groups decreases the electron density around the astatine, increases its oxidation potential and thereby enhances resistance to oxidative decomposition. Additionally, ortho substituents provide steric hindrance, limiting enzymatic access [204].

Quantum mechanical calculations offer a promising tool for advancing ^211^At‐radiopharmaceutical development. These methods effectively estimate bond enthalpies, correlating with drug candidates’ in vivo stability. Notably, findings suggest deastatination is primarily influenced by the immediate atomic environment around the ^211^At attachment site, rather than the overall molecular structure [31].

## 8 Clinical Landscape—Where Are We?

The clinical relevance of ^211^At‐labeled radiopharmaceuticals continues to grow, driven by advances in radiolabeling techniques and deeper understanding of their in vivo behavior. While research is still ongoing to fully elucidate the broader chemistry of ^211^At, several promising candidates have progressed into clinical trials. This section provides a concise overview of selected trials; for a more comprehensive discussion, readers are referred to detailed reviews [88, 205]. One interesting trial involves the use of [^211^At]**At** ^**‐**^ in patients with thyroid cancer [206]. Like iodine, ^211^At is actively transported into the thyroid via the sodium‐iodide symporter, which is overexpressed in thyroid cancer cells. A Phase I clinical trial (NCT05275946) involving 11 patients is currently assessing the optimal dosing of [^211^At]**NaAt**, with results anticipated in 2025 [207, 208, 209, 210]. In another Phase I trial (NCT04461457), the safety of intraperitoneally administered ^211^At‐**MX35‐F(ab’)**_**2**_ (20–215 MBq/L) was evaluated for treating recurrent epithelial ovarian cancers in patients who had undergone near‐complete second‐line chemotherapy. Minimal adverse effects from the therapy were observed [69]. Planned to start recruitment early 2025 is a Phase I clinical trial (NCT04579523) to investigate the safety and dosing of astatinated murine IgG_1_ anti‐CD38 mAb (^211^At‐**OKT10‐B10)** in multiple myeloma. The treatment will be combined with chemotherapeutic drugs and low‐dose total body irradiation in 30 patients, aiming to eliminate residual tumor cells before donor stem cell transplantation. Another planned Phase I trial will assess the safety, pharmacokinetics and optimal dosing of [^211^At]**MABG** to treat malignant pheochromocytoma and paraganglioma. The compound mimics norepinephrine and is internalized by cells expressing norepinephrine transporters, such as those in neuroblastoma. Patients will receive escalating doses of 0.65, 1.3, and 2.6 MBq/kg to determine the maximum tolerated and recommended dose [211]. Results from this trial are expected in 2025 [211, 212]. Lastly, Phase I clinical trial (NCT06441994) is recruiting 15 patients with castration‐resistant prostate cancer to evaluate tolerability, safety, pharmacokinetics, absorbed dose and efficacy of [^211^At]‐**PSMA‐5** [213]. Preclinical evaluation in normal male ICR mice and cynomolgus monkeys showed no severe and only reversible toxicity [214], though mild leukopenia was observed in monkeys 24 h post‐injection. Despite no histological abnormalities, high accumulation was noted in the kidneys and thyroid for both monkeys and mice (estimated human absorbed doses: 4.05 mGy/MBq in kidneys, 1.82 mGy/MBq in thyroid) [214]. The thyroid uptake indicates deastatination. Recently, the first‐in‐human SPECT/CT image of [^211^At]PSMA‐5 in a patient with refractory prostate cancer was published [215]. Targeting the 79 keV X‐rays from daughter ^211^Po, SUVmax values of 4.9 and 17.6 were observed in the prostate and an external lymph node metastasis, respectively [215]. Although the number of clinical trials involving ^211^At radiopharmaceutical remains low, and few have been completed, it underlines the lacking understanding of the radionuclide's behavior. However, and as will be described below, the interest is continuously growing, the understanding is enhanced, and several additional clinical trials can be expected in the coming years.

**Is the deastatination of ^211^At‐labeled radiopharmaceuticals under control?**

Deastatination remains a major challenge in the development of ^211^At‐labeled radiopharmaceuticals. Progress in understanding and mitigating this issue has been limited by the scarce availability of astatine and the lack of a stable isotope. Although the exact chemical and biological mechanisms underlying deastatination are not fully understood, they are likely to involve multiple factors. Recent advancements have improved our understanding of astatine's in vivo behavior. These include experimental determination of astatine's electron affinity and electronegativity [33], the elucidation of the Pourbaix diagram [34, 39], and improvements in computational modeling algorithms [89, 216, 217]. Proposed mechanisms—such as lysosomal degradation, oxidative susceptibility, and enzymatic cleavage ‐ have guided the design of molecular scaffolds that substantially reduce or even prevent deastatination. Despite these promising developments, the number clinical trials involving astatine remains low, highlighting the need for further refinement before it can achieve widespread clinical use. Nonetheless, promising approaches, such as guanidinomethyl functionalization [168, 218], neopentyl glycol scaffolds [90, 91], and AuNPs [219, 220], offer encouraging prospects for the successful clinical translation of ^211^At‐radiopharmaceuticals.

## 9 Preclinical Landscape—What's Next on the Horizon?

**Figure 23:**
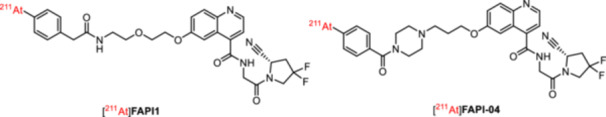
^211^At‐labeled FAP targeting agents.

In recent years, significant progress has been made in the development of ^211^At‐labeled radiopharmaceuticals, with a particular focus on improving the therapeutic efficacy of previously FDA‐approved radioligands. Notably, efforts to design ^211^At‐labeled PSMA‐targeting vectors have yielded promising results. The first reported candidate, [²¹¹At]**DCABzL** (Figure 18A), demonstrated high tumor accumulation and improved overall median survival in PSMA‐positive PC3 PIP‐bearing mouse xenografts. However, its clinical potential was limited by high kidney retention and dehalogenation [125]. To address these challenges, [^211^At]**7‐Lu** (Figure 12) was developed, offering enhanced characteristics, including high tumor uptake and improved tumor‐to‐background ratios [125]. Treatment with [^211^At]**7**‐**Lu** extended median survival in PSMA‐positive PC3 PIP‐bearing mice from 48 days (untreated) to 58.5 days at a dose of 3.7 MBq [125]. More recently, additional ^211^At‐labeled PSMA inhibitors have been developed, such as [^211^At]**8**‐Ga (Figure 13), which exhibited excellent in vivo biodistribution, characterized by high tumor uptake, rapid renal clearance, and low kidney retention. Minimal deastatination further supports its clinical potential [87, 135]. Interestingly, first ^211^At‐labeled FAP‐targeting agents showed promise to treat cancers even though cancer cells are not directly targeted in this approach [75, 77]. Compounds such as [^211^At]**FAPI1** and [^211^At]**FAPI‐04** (Figure 23) significantly inhibited tumor growth in animal models, although their exact mechanism of action remains unclear [75, 77]. Enhancing tumor retention of FAP‐targeting agents could further improve their therapeutic efficacy. Numerous other ^211^At‐labeled targeting agents are under investigation. Looking at the industrial pipeline, Telix Pharmaceuticals is planning a Phase I clinical trial for [^211^At]‐**APA** (TLX102) for glioma treatment. This compound accumulates in tumor cells via LAT1 transporter‐mediated internalization. Minerva Imaging and Atonco have partnered to produce clinical doses of ^211^At‐Girentuximab (TLX‐250) for a Phase I clinical trial targeting non‐muscle‐invasive bladder cancer. Girentuximab is an anti‐ carbonic anhydrase IX antibody, targeting an antigen expressed on the surface of cancer cell. Additionally, Precision Molecular, launched a clinical trial in 2024 for ^211^At‐labeled PSMA‐targeting radiopharmaceutical (**PMI21**), with results expected in 2025 [221]. These developments underscore the growing potential of ^211^At‐based therapies in clinical oncology. For a more comprehensive discussion, readers are encouraged to consult additional reviews [88, 205].

## 10 The Future of Astatine‐211: Research and Commercial Outlook

Astatine‐211 holds a unique position among α‐emitters for RLT. As the only α‐emitter capable of forming covalent bonds, it enables the development of radiopharmaceuticals that can cross the blood‐brain barrier or penetrate cells, distinguishing it from all radiometal‐based α‐emitters. This ability opens new therapeutic possibilities for treating a broad range of cancers. Additionally, ^211^At's decay properties—emitting a single α‐particle per decay, accompanying gamma emissions for imaging, and a relatively short half‐life—make it ideal for precise tumor targeting, controlled radiation delivery, and streamlined waste management, including minimal radioactive excreta from patients. These advantages have generated significant interest in ^211^At's therapeutic potential, even in more traditional RLT approaches targeting extracellular proteins. To fully capitalize on its potential, substantial investments are needed to scale up its production and infrastructure. Although upscaling technologies are available, additional production facilities are essential to ensure broader accessibility. The success of ongoing clinical trials, with results expected in 2025, will be pivotal in driving industrial interest and accelerating adoption. However, challenges such as deastatination must be addressed for ^211^At to reach its full clinical potential. Promising stabilization strategies—such as guanidinomethyl functionalization, neopentyl glycol scaffolds, and gold nanoparticles—have shown encouraging results, though further validation is needed. Ongoing improvements will likely be necessary to develop in vivo stable astatinated radiopharmaceuticals, with advancements likely driven by computational modeling and artificial intelligence design. Over the next 10–15 years, research will likely focus on combining ^211^At with immunotherapy and chemotherapy to enhance treatment outcomes, as well as exploring fractionated dosing strategies. Clinical trials targeting aggressive cancers—such as glioblastoma, metastatic castration‐resistant prostate cancer, and thyroid malignancies—will play a key role in securing regulatory approvals and facilitating commercialization. To support these advancements, commercially available and affordable ^211^At‐isolation and synthesis modules must become widespread, reducing barriers from bench to bedside.

By 2035, we expect ^211^At to become a cornerstone of targeted α‐therapy, revolutionizing cancer treatment and driving new developments in radiopharmaceuticals. While challenges remain in scaling production and reducing costs, ongoing innovation and global investment will be essential to unlocking ^211^At's full therapeutic potential. The future of ^211^At in oncology is promising and represents an exciting frontier in modern medicine.

## Conflicts of Interest

Vladimir Shalgunov, Andreas Ingemann Jensen, and Umberto Maria Battisti are founders and partly employed at TetraKit Technologies ApS—a company developing radiopharmaceuticals based on astatine. Matthias Manfred Herth is a consultant and founder of TetraKit Technologies ApS and PreTT ApS. Andreas Ingemann Jensen and Matthias Manfred Herth are founders of Theranostic Solutions.

## Data Availability

Data sharing is not applicable to this article as no new data were created or analyzed in this study.

## References

[1] O. Sartor , J. de Bono , K. N. Chi , et al., “Lutetium‐177–PSMA‐617 for Metastatic Castration‐Resistant Prostate Cancer,” New England Journal of Medicine 385 (2021): 1091–1103. 34161051 10.1056/NEJMoa2107322 PMC8446332

[2] J. Strosberg , G. El‐Haddad , E. Wolin , et al., “Phase 3 Trial of177Lu‐Dotatate for Midgut Neuroendocrine Tumors,” New England Journal of Medicine 376 (2017): 125–135. 28076709 10.1056/NEJMoa1607427 PMC5895095

[3] S. Singh , D. Halperin , S. Myrehaug , et al., “177Lu]Lu‐DOTA‐TATE Plus Long‐Acting Octreotide Versus High‑Dose Long‐Acting Octreotide for the Treatment of Newly Diagnosed, Advanced Grade 2–3, Well‐Differentiated, Gastroenteropancreatic Neuroendocrine Tumours (NETTER‐2): an Open‐Label, Randomised, Phase 3 Study,” Lancet 403 (2024): 2807–2817. 38851203 10.1016/S0140-6736(24)00701-3

[4] S. Hoshi , K. Yaginuma , S. Meguro , et al., “PSMA Targeted Molecular Imaging and Radioligand Therapy for Prostate Cancer: Optimal Patient and Treatment Issues,” Current Oncology 30 (2023): 7286–7302. 37623010 10.3390/curroncol30080529 PMC10453875

[5] Novartis, Novartis Lutathera® significantly Reduced Risk of Disease Progression or Death by 72% as First‐Line Treatment for Patients With Advanced Gastroenteropancreatic Neuroendocrine Tumors, accessed August 30, 2024.

[6] Novartis, Novartis Pluvicto™ Shows Clinically Meaningful and Highly Statistically Significant Rpfs Benefit in Patients With Psma‐positive Metastatic Castration‐resistant Prostate Cancer in the Pre‐taxane Setting, accessed August 30, 2024.

[7] C. Kratochwil , F. Bruchertseifer , F. L. Giesel , et al., “225Ac‐PSMA‐617 for PSMA‐Targeted α‐Radiation Therapy of Metastatic Castration‐Resistant Prostate Cancer,” Journal of Nuclear Medicine 57 (2016): 1941–1944. 27390158 10.2967/jnumed.116.178673

[8] E. Daguenet , S. Louati , A.‐S. Wozny , et al., “Radiation‐Induced Bystander and Abscopal Effects: Important Lessons From Preclinical Models,” British Journal of Cancer 123 (2020): 339–348. 32581341 10.1038/s41416-020-0942-3 PMC7403362

[9] H. Tang , L. Cai , X. He , et al., “Radiation‐Induced Bystander Effect and its Clinical Implications,” Frontiers in Oncology 13 (2023): 1124412. 37091174 10.3389/fonc.2023.1124412 PMC10113613

[10] S. Poty , L. C. Francesconi , M. R. McDevitt , M. J. Morris , and J. S. Lewis , “α‐Emitters for Radiotherapy: From Basic Radiochemistry to Clinical Studies—Part 1,” Journal of Nuclear Medicine 59 (2018): 878–884. 29545378 10.2967/jnumed.116.186338 PMC6004557

[11] F. Graf , J. Fahrer , S. Maus , et al., “DNA Double Strand Breaks as Predictor of Efficacy of the Alpha‐Particle Emitter Ac‐225 and the Electron Emitter Lu‐177 for Somatostatin Receptor Targeted Radiotherapy,” PLoS One 9 (2014): e88239. 24516620 10.1371/journal.pone.0088239 PMC3917860

[12] S. Poty , L. C. Francesconi , M. R. McDevitt , M. J. Morris , and J. S. Lewis , “α‐Emitters for Radiotherapy: From Basic Radiochemistry to Clinical Studies—Part 2,” Journal of Nuclear Medicine 59 (2018): 1020–1027. 29496984 10.2967/jnumed.117.204651 PMC6910645

[13] A. Belchior , I. Balásházy , O. M. Gil , P. Vaz , and P. Almeida , “Does the Number of Irradiated Cells Influence the Spatial Distribution of Bystander Effects,” Dose‐Response 12 (2014): 525. 25552955 10.2203/dose-response.14-001.Belchior PMC4267447

[14] M. Boyd , S. C. Ross , J. Dorrens , et al., “Radiation‐Induced Biologic Bystander Effect Elicited In Vitro by Targeted Radiopharmaceuticals Labeled With Alpha‐, Beta‐, and Auger Electron‐Emitting Radionuclides,” Journal of Nuclear Medicine: Official Publication, Society of Nuclear Medicine 47 (2006): 1007–1015. 16741311

[15] M. Trujillo‐Nolasco , E. Morales‐Avila , P. Cruz‐Nova , K. Katti , and B. Ocampo‐García , “Nanoradiopharmaceuticals Based on Alpha Emitters: Recent Developments for Medical Applications,” Pharmaceutics 13 (2021): 1123. 34452084 10.3390/pharmaceutics13081123 PMC8398190

[16] H. Yang , J. J. Wilson , C. Orvig , et al., “Harnessingα‐Emitting Radionuclides for Therapy: Radiolabeling Method Review,” Journal of Nuclear Medicine 63 (2022): 5–13. 34503958 10.2967/jnumed.121.262687 PMC8717181

[17] D. S. Wilbur , “Enigmatic Astatine,” Nature Chemistry 5 (2013): 246. 10.1038/nchem.1580 23422568

[18] G. Vaidyanathan and M. Zalutsky , “Astatine Radiopharmaceuticals: Prospects and Problems,” Current Radiopharmaceuticalse 1 (2008): 177–196. 10.2174/1874471010801030177 PMC2818997 20150978

[19] F. Guérard , C. Maingueneau , L. Liu , et al., “Advances in the Chemistry of Astatine and Implications for the Development of Radiopharmaceuticals,” Accounts of Chemical Research 54 (2021): 3264–3275. 10.1021/acs.accounts.1c00327 34350753

[20] G. J. Meyer , “Astatine,” Journal of Labelled Compounds and Radiopharmaceuticals 61 (2018): 154–164. 29080397 10.1002/jlcr.3573

[21] A. Yagishita , M. Katsuragawa , S. I Takeda , et al., “Development and Utility of an Imaging System for Internal Dosimetry of Astatine‐211 in Mice,” Bioengineering 11 (2024): 25. 10.3390/bioengineering11010025 PMC11154565 38247903

[22] I. Nishinaka , K. Hashimoto , and H. Suzuki , “Thin Layer Chromatography for Astatine and Iodine in Solutions Prepared by Dry Distillation,” Journal of Radioanalytical and Nuclear Chemistry 318 (2018): 897–905.

[23] Y. Shin , S. Maruyama , K. Kawasaki , et al., “Solvent Extraction Following Oxidation of Astatine for the Use of a 211Rn–211At Generator,” Journal of Radioanalytical and Nuclear Chemistry 333 (2023): 403–409.

[24] D. Wilbur , “211At]Astatine‐Labeled Compound Stability: Issues With Released [211At]Astatide and Development of Labeling Reagents to Increase Stability,” Current Radiopharmaceuticalse 1 (2008): 144–176.

[25] R. E. Mclendon , G. E. Archer , R. H. Larsen , G. Akabani , D. D. Bigner , and M. R. Zalutsky , “Radiotoxicity of Systemically Administered 211At‐labeled Human/Mouse Chimeric Monoclonal Antibody: A Long‐Term Survival Study With Histologic Analysis,” International Journal of Radiation Oncology*Biology*Physics 45 (1999): 491–499. 10.1016/s0360-3016(99)00206-0 10487576

[26] S. Naka , K. Ooe , Y. Shirakami , et al., “Production of [211At]NaAt Solution Under GMP Compliance for Investigator‐Initiated Clinical Trial,” EJNMMI Radiopharmacy and Chemistry 9 (2024): 29. 38619655 10.1186/s41181-024-00257-z PMC11018728

[27] F. Guérard , J. F. Gestin , and M. W. Brechbiel , “Production of [(211)At]‐Astatinated Radiopharmaceuticals and Applications in Targeted α‐particle Therapy,” Cancer Biotherapy & Radiopharmaceuticals 28 (2013): 1–20. 23075373 10.1089/cbr.2012.1292 PMC3545490

[28] T. Watabe , K. Kaneda‐Nakashima , Y. Liu , et al., “Enhancement of 211At Uptake via the Sodium Iodide Symporter by the Addition of Ascorbic Acid in Targeted α‐Therapy of Thyroid Cancer,” Journal of Nuclear Medicine 60 (2019): 1301–1307. 30796173 10.2967/jnumed.118.222638 PMC6735285

[29] R. E. Vernon , “Which Elements Are Metalloids,” Journal of Chemical Education 90 (2013): 1703–1707.

[30] G. Restrepo , E. J. Llanos , and H. Mesa , “Topological Space of the Chemical Elements and Its Properties,” Journal of Mathematical Chemistry 39 (2005): 401–416.

[31] T. Ayed , J. Pilmé , D. Tézé , et al., “211 At‐Labeled Agents for Alpha‐Immunotherapy: on the In Vivo Stability of Astatine‐Agent Bonds,” European Journal of Medicinal Chemistry 116 (2016): 156–164. 27061979 10.1016/j.ejmech.2016.03.082

[32] K. T. Giju , F. De Proft , and P. Geerlings , “Comprehensive Study of Density Functional Theory Based Properties For Group 14 Atoms and Functional Groups, ‐XY3 (X = C, Si, Ge, Sn, Pb, Element 114; Y = CH3, H, F, Cl, Br, I, At),” Journal of Physical Chemistry A 109 (2005): 2925–2936. 16833611 10.1021/jp050463x

[33] D. Leimbach , J. Karls , Y. Guo , et al., “The Electron Affinity of Astatine,” Nature Communications 11 (2020): 3824. 10.1038/s41467-020-17599-2 PMC7393155 32733029

[34] L. Liu , R. Maurice , N. Galland , P. Moisy , J. Champion , and G. Montavon , “Pourbaix Diagram of Astatine Revisited: Experimental Investigations,” Inorganic Chemistry 61 (2022): 13462–13470. 35977097 10.1021/acs.inorgchem.2c01918

[35] N. Guo , F. Pottier , J. Aupiais , C. Alliot , G. Montavon , and J. Champion , “Evidence for the Heaviest Expected Halide Species in Aqueous Solution, At–, by Electromobility Measurements,” Inorganic Chemistry 57 (2018): 4926–4933. 29652492 10.1021/acs.inorgchem.7b03003

[36] A. Sabatié‐Gogova , J. Champion , S. Huclier , et al., “Characterization of At− Species in Simple and Biological Media by High Performance Anion Exchange Chromatography Coupled to Gamma Detector,” Analytica Chimica Acta 721 (2012): 182–188. 22405318 10.1016/j.aca.2012.01.052

[37] J. Champion , C. Alliot , E. Renault , et al., “Astatine Standard Redox Potentials and Speciation in Acidic Medium,” Journal of Physical Chemistry A 114 (2010): 576–582. 20014840 10.1021/jp9077008

[38] J. Champion , A. Sabatié‐Gogova , F. Bassal , et al., “Investigation of Astatine(III) Hydrolyzed Species: Experiments and Relativistic Calculations,” Journal of Physical Chemistry A 117 (2013): 1983–1990. 23373677 10.1021/jp3099413

[39] D.‐C. Sergentu , D. Teze , A. Sabatié‐Gogova , et al., “Advances on the Determination of the Astatine Pourbaix Diagram: Predomination of AtO(OH)2−over At−in Basic Conditions,” Chemistry – A European Journal 22 (2016): 2964–2971. 26773333 10.1002/chem.201504403

[40] E. H. Appelman , “The Oxidation States of Astatine in Aqueous Solution1,” Journal of the American Chemical Society 83 (1961): 805–807.

[41] I. Nishinaka , K. Hashimoto , and H. Suzuki , “Speciation of Astatine Reacted With Oxidizing and Reducing Reagents by Thin Layer Chromatography: Formation of Volatile Astatine,” Journal of Radioanalytical and Nuclear Chemistry 322 (2019): 2003–2009.

[42] K. Rössler , W. Tornau , and G. Stöcklin , “Rapid Separation of Carrier‐Free Inorganic and Organic Compounds of Radioiodine and Astatine by High‐Pressure Liquid Chromatography,” Journal of Radioanalytical Chemistry 21 (1974): 199–209.

[43] D. R. Corson , K. R. MacKenzie , and E. Segrè , “Artificially Radioactive Element 85,” Physical Review 58 (1940): 672–678.

[44] A. Alfarano , K. Abbas , U. Holzwarth , et al., “Thick Target Yield Measurement of 211At Through the Nuclear Reaction209Bi(α, 2n,” Journal of Physics: Conference Series 41 (2006): 115–122.

[45] E. Appelman , *The Radiochemistry of Astatine*, Natl Acad. Sci Natl. Res Counc, Nucl Sci Ser, U.S.At Energy Comm (1960).

[46] J. Merinis and G. Bouissieres , “Étude de la migration de radioéléments dans un tube à gradient de température,” ract 12 (1969): 140–152.

[47] R. Dreyer , I. Dreyer , W. Doberenz , and S. Fischer , “Zur anorganischcn Cheinie dcs monovaleiilcii Astafs,” Isotopenpraxis 22, no. 3 (1986): 81.

[48] R. D. Neirinckx and J. A. Smit , “Separation of Astatine‐211 From Bismuth Metal,” Analytica Chimica Acta 63 (1973): 201–204. 4682975 10.1016/S0003-2670(01)82189-9

[49] H. M. Neumann , “Solvent Distribution Studies of the Chemistry of Astatine,” Journal of Inorganic and Nuclear Chemistry 4 (1957): 349–353.

[50] V. Doberenz , N. Dang Duc , R. Dreyer , M. Milanov , Y. V. Norseyev , and V. A. Khalkin , “Preparation of Astatine of High Specific Activity in Solutions of a Given Composition,” Radiochem Radioanal Letters 52 (1982): 119–127.

[51] S. Lindegren , T. Bäck , and H. J. Jensen , “Dry‐Distillation of Astatine‐211 From Irradiated Bismuth Targets: A Time‐Saving Procedure With High Recovery Yields,” Applied Radiation and Isotopes 55 (2001): 157–160. 11393754 10.1016/s0969-8043(01)00044-6

[52] E. Aneheim , P. Albertsson , T. Bäck , H. Jensen , S. Palm , and S. Lindegren , “Automated Astatination of Biomolecules – A Stepping Stone Towards Multicenter Clinical Trials,” Scientific Reports 5 (2015): 12025. 26169786 10.1038/srep12025 PMC4500947

[53] S. W. Hadley , D. S. Wilbur , M. A. Gray , and R. W. Atcher , “Astatine‐211 Labeling of an Antimelanoma Antibody and Its Fab Fragment Using N‐Succinimidyl p‐[211At]Astatobenzoate: Comparisons In Vivo With the p‐[125I]Iodobenzoyl Conjugate,” Bioconjugate Chemistry 2 (1991): 171–179. 1932216 10.1021/bc00009a006

[54] D. S. Wilbur , R. L. Vessella , J. E. Stray , D. K. Goffe , K. A. Blouke , and R. W. Atcher , “Preparation and Evaluation of Para‐[211At]Astatobenzoyl Labeled Anti‐Renal Cell Carcinoma Antibody A6H F(ab′)2. In Vivo Distribution Comparison With Para‐[125I]Iodobenzoyl Labeled A6H F(ab′)2,” Nuclear Medicine and Biology 20 (1993): 917–927. 8298571 10.1016/0969-8051(93)90092-9

[55] U. P. Schwarz , P. Plascjak , M. P. Beitzel , O. A. Gansow , W. C. Eckelman , and T. A. Waldmann , “Preparation of 211At‐Labeled Humanized Anti‐Tac Using 211At Produced in Disposable Internal and External Bismuth Targets,” Nuclear Medicine and Biology 25 (1998): 89–93. 9468021 10.1016/s0969-8051(97)00165-0

[56] J. Koziorowski , O. Lebeda , and R. Weinreich , “A Cryotrap as Flow Reactor for Synthesis of 211At Labelled Compounds,” Applied Radiation and Isotopes 50 (1999): 527–529.

[57] D. S. Wilbur , S. W. Hadley , J. J. Hines , and R. W. Archer , “Assessment of dry Distillation Methods for Improving Protein Labeling Yields With Astatine‐211,” Journal of Labelled Compounds and Radiopharmaceuticals 30 (1991): 214–215.

[58] M. B. Sevenois , H. J. Jensen , F. Haddad , et al., “Optimised Solid‐Phase Extraction of 211At: Activity Balance of 211At, 210At and 210Po After Wet Chemistry Target Dissolution,” Radiation Physics and Chemistry 225 (2024): 112146.

[59] J. D. Burns , E. E. Tereshatov , G. Avila , et al., “Rapid Recovery of At‐211 by Extraction Chromatography,” Separation and Purification Technology 256 (2021): 117794.

[60] E. Balkin , D. Hamlin , K. Gagnon , et al., “Evaluation of a Wet Chemistry Method for Isolation of Cyclotron Produced [211At]Astatine,” Applied Sciences 3 (2013): 636–655.

[61] C. Zona , M. L. Bonardi , F. Groppi , et al., “Wet‐Chemistry Method for the Separation of No‐Carrier‐Added 211At/211gPo From 209Bi Target Irradiated by Alpha‐Beam in Cyclotron,” Journal of Radioanalytical and Nuclear Chemistry 276 (2008): 819–824.

[62] E. E. Tereshatov , J. D. Burns , S. J. Schultz , et al., “Compact Automated Apparatus for Rapid Astatine Recovery From Nitric Acid Media: Design, Application, and Impurity Characterization,” Chemical Engineering Journal 442 (2022): 136176.

[63] L. A. McIntosh , J. D. Burns , E. E. Tereshatov , et al., “Production, Isolation, and Shipment of Clinically Relevant Quantities of Astatine‐211: A Simple and Efficient Approach to Increasing Supply,” Nuclear Medicine and Biology 126–127 (2023): 108387. 10.1016/j.nucmedbio.2023.108387 37837782

[64] Y. Feng and M. R. Zalutsky , “Production, Purification and Availability of 211At: Near Term Steps Towards Global Access,” Nuclear Medicine and Biology 100–101 (2021): 12–23. 10.1016/j.nucmedbio.2021.05.007 PMC8448941 34144505

[65] M. W. Brechbiel , “Targeted Α‐Therapy,” Cancer Biotherapy & Radiopharmaceuticals 35 (2020): 397. 32503377 10.1089/cbr.2020.29008.mbr

[66] S. Lindegren , P. Albertsson , T. Bäck , H. Jensen , S. Palm , and E. Aneheim , “Realizing Clinical Trials With Astatine‐211: The Chemistry Infrastructure,” Cancer Biotherapy & Radiopharmaceuticals 35 (2020): 425–436. 32077749 10.1089/cbr.2019.3055 PMC7465635

[67] E. S. Delpassand , I. Tworowska , R. Esfandiari , et al., “Targetedα‐Emitter Therapy with 212Pb‐DOTAMTATE for the Treatment of Metastatic SSTR‐Expressing Neuroendocrine Tumors: First‐in‐Humans Dose‐Escalation Clinical Trial,” Journal of Nuclear Medicine 63 (2022): 1326–1333. 34992153 10.2967/jnumed.121.263230 PMC9454455

[68] M. R. Zalutsky , D. A. Reardon , G. Akabani , et al., “Clinical Experience With α‐Particle–Emitting 211At: Treatment of Recurrent Brain Tumor Patients with 211At‐Labeled Chimeric Antitenascin Monoclonal Antibody 81C6,” Journal of Nuclear Medicine 49 (2008): 30–38. 18077533 10.2967/jnumed.107.046938 PMC2832604

[69] A. Hallqvist , K. Bergmark , T. Bäck , et al., “Intraperitoneal α‐Emitting Radioimmunotherapy with 211At in Relapsed Ovarian Cancer: Long‐Term Follow‐Up With Individual Absorbed Dose Estimations,” Journal of Nuclear Medicine 60 (2019): 1073–1079. 30683761 10.2967/jnumed.118.220384 PMC6681696

[70] A. K. H. Robertson , C. F. Ramogida , P. Schaffer , and V. Radchenko , “Development of225Ac Radiopharmaceuticals: TRIUMF Perspectives and Experiences,” Current Radiopharmaceuticals 11 (2018): 156–172. 29658444 10.2174/1874471011666180416161908 PMC6249690

[71] F. Kansteiner , Bristol Myers’ RayzeBio Halts Radiotherapy Trial Enrollment After Isotope Runs Scarce, accessed August 30, 2024.

[72] R. Zimmermann , “Is Actinium Really Happening?,” Journal of Nuclear Medicine 64, no. 10 (2023): 1615–1518. 10.2967/jnumed.123.265907 37591546

[73] S. Ballal , M. P. Yadav , E. S. Moon , et al., “First‐in‐Human Results on the Biodistribution, Pharmacokinetics, and Dosimetry of [177Lu]Lu‐DOTA.SA.FAPi and [177Lu]Lu‐DOTAGA.(SA.FAPi)2,” Pharmaceuticals 14 (2021): 1212. 34959613 10.3390/ph14121212 PMC8707268

[74] R. Zimmermann , *Oncidinium Foundation*.

[75] H. Ma , F. Li , G. Shen , et al., “In Vitro and In Vivo Evaluation of 211At‐Labeled Fibroblast Activation Protein Inhibitor for Glioma Treatment,” Bioorganic and Medicinal Chemistry 55 (2022): 116600. 34999526 10.1016/j.bmc.2021.116600

[76] W. P. Fendler , K. M. Pabst , L. Kessler , et al., “Safety and Efficacy of 90Y‐FAPI‐46 Radioligand Therapy in Patients With Advanced Sarcoma and Other Cancer Entities,” Clinical Cancer Research 28 (2022): 4346–4353. 35833949 10.1158/1078-0432.CCR-22-1432 PMC9527500

[77] A. Aso , H. Nabetani , Y. Matsuura , et al., “Evaluation of Astatine‐211‐Labeled Fibroblast Activation Protein Inhibitor (FAPI): Comparison of Different Linkers With Polyethylene Glycol and Piperazine,” International Journal of Molecular Sciences 24 (2023): 8701. 37240044 10.3390/ijms24108701 PMC10218645

[78] T. Jabbar , S. Bashir , and M. I. Babar , “Review of Current Status of Targeted Alpha Therapy in Cancer Treatment,” Nuclear Medicine Review 26 (2023): 54–67. 38966955 10.5603/NMR.2023.0003

[79] A. Morgenstern and F. Bruchertseifer , “Development of Targeted Alpha Therapy From Bench to Bedside,” Journal of Medical Imaging and Radiation Sciences 50 (2019): S18–S20. 31405818 10.1016/j.jmir.2019.06.046

[80] S. J. Dovedi , A. L. Adlard , G. Lipowska‐Bhalla , et al., “Acquired Resistance to Fractionated Radiotherapy Can be Overcome by Concurrent PD‐L1 Blockade,” Cancer Research 74 (2014): 5458–5468. 25274032 10.1158/0008-5472.CAN-14-1258

[81] M. Z. Dewan , A. E. Galloway , N. Kawashima , et al., “Fractionated but Not Single‐Dose Radiotherapy Induces an Immune‐Mediated Abscopal Effect When Combined With Anti–CTLA‐4 Antibody,” Clinical Cancer Research 15 (2009): 5379–5388. 19706802 10.1158/1078-0432.CCR-09-0265 PMC2746048

[82] L. A. Carvalho , R. Fleming , M. Sant'Anna , et al., “Neuroprotective Effects of Erythropoietin on Rat Retinas Subjected to Oligemia,” Clinics 73 (2018): e557s. 29694605 10.6061/clinics/2018/e161 PMC5890171

[83] G. Sgouros , G. Ulaner , T. Delie , et al., “225Ac‐DOTATATE Dosimetry Results From Part 1 of the ACTION‐1 Trial,” Journal of Nuclear Medicine 64 (2023): 129.

[84] B. Molina , J. R. Soto , and J. J. Castro , “Halogen‐Like Properties of the Al13cluster Mimicking Astatine,” Physical Chemistry Chemical Physics 20 (2018): 11549–11553. 29651478 10.1039/c8cp00494c

[85] S. W. Schwarz and C. Decristoforo , “US and EU Radiopharmaceutical Diagnostic and Therapeutic Nonclinical Study Requirements for Clinical Trials Authorizations and Marketing Authorizations,” EJNMMI Radiopharmacy and Chemistry 4 (2019): 10. 31659486 10.1186/s41181-019-0059-2 PMC6529498

[86] A. Korde , R. Mikolajczak , P. Kolenc , et al., “Practical Considerations for Navigating the Regulatory Landscape of Non‐Clinical Studies for Clinical Translation of Radiopharmaceuticals,” EJNMMI Radiopharmacy and Chemistry 7 (2022): 18. 35852679 10.1186/s41181-022-00168-x PMC9296747

[87] T. Watabe , K. Kaneda‐Nakashima , Y. Shirakami , et al., “Targeted α‐Therapy Using Astatine (211At)‐Labeled PSMA1, 5, and 6: A Preclinical Evaluation as a Novel Compound,” European Journal of Nuclear Medicine and Molecular Imaging 50 (2023): 849–858. 36344651 10.1007/s00259-022-06016-z PMC9852121

[88] P. Albertsson , T. Bäck , K. Bergmark , et al., “Astatine‐211 Based Radionuclide Therapy: Current Clinical Trial Landscape,” Frontiers in Medicine 9 (2023): 1076210. 36687417 10.3389/fmed.2022.1076210 PMC9859440

[89] T. Yssartier , L. Liu , S. Pardoue , et al., “In Vivostability of 211At‐Radiopharmaceuticals: On the Impact of Halogen Bond Formation,” RSC Medicinal Chemistry 15 (2024): 223–233. 38283213 10.1039/d3md00579h PMC10809332

[90] H. Suzuki , Y. Kaizuka , M. Tatsuta , et al., “Neopentyl Glycol as a Scaffold to Provide Radiohalogenated Theranostic Pairs of High In Vivo Stability,” Journal of Medicinal Chemistry 64 (2021): 15846–15857. 34708646 10.1021/acs.jmedchem.1c01147

[91] H. Suzuki , K. Kannaka , M. Hirayama , et al., “In Vivo Stable 211At‐Labeled Prostate‐Specific Membrane Antigen‐Targeted Tracer Using a Neopentyl Glycol Structure,” EJNMMI Radiopharm Chem 9 (2024): 48. 38884866 10.1186/s41181-024-00278-8 PMC11183015

[92] E. J. L. Stéen , P. E. Edem , K. Nørregaard , et al., “Pretargeting in Nuclear Imaging and Radionuclide Therapy: Improving Efficacy of Theranostics and Nanomedicines,” Biomaterials 179 (2018): 209–245. 30007471 10.1016/j.biomaterials.2018.06.021

[93] S. M. Kondengadan , S. Bansal , C. Yang , D. Liu , Z. Fultz , and B. Wang , “Click Chemistry and Drug Delivery: A Bird's‐Eye View,” Acta Pharmaceutica Sinica B 13 (2023): 1990–2016. 37250163 10.1016/j.apsb.2022.10.015 PMC10213991

[94] E. Aneheim , E. Hansson , C. Timperanza , H. Jensen , and S. Lindegren , “Behaviour, Use and Safety Aspects of Astatine‐211 Solvated in Chloroform After Dry Distillation Recovery,” Scientific Reports 14 (2024): 9698. 38678056 10.1038/s41598-024-60615-4 PMC11055885

[95] E. Aneheim , S. Palm , H. Jensen , C. Ekberg , P. Albertsson , and S. Lindegren , “Towards Elucidating the Radiochemistry of Astatine – Behavior in Chloroform,” Scientific Reports 9 (2019): 15900. 31685874 10.1038/s41598-019-52365-5 PMC6828679

[96] M. Ghalei , P. Mahdi Khoshouei , J. Vandenborre , et al., “Towards Elucidating the Radiochemistry of Sstatine ‐ Behavior in Chloroform,” Radiation Physics and Chemistry 198 (2022): 110224.

[97] Y. Shirakami , T. Watabe , H. Obata , et al., “Synthesis of [211At]4‐Astato‐L‐Phenylalanine by Dihydroxyboryl‐Astatine Substitution Reaction in Aqueous Solution,” Scientific Reports 11 (2021): 12982. 34155314 10.1038/s41598-021-92476-6 PMC8217504

[98] M. Vanermen , M. Ligeour , M. C. Oliveira , et al., “Astatine‐211 Radiolabelling Chemistry: From Basics to Advanced Biological Applications,” EJNMMI Radiopharmacy and Chemistry 9 (2024): 69. 39365487 10.1186/s41181-024-00298-4 PMC11452365

[99] M. M. Herth , S. Ametamey , D. Antuganov , et al., “On the Consensus Nomenclature Rules for Radiopharmaceutical Chemistry – Reconsideration of Radiochemical Conversion,” Nuclear Medicine and Biology 93 (2021): 19–21. 33232876 10.1016/j.nucmedbio.2020.11.003

[100] F. Guérard , L. Navarro , Y.‐S. Lee , et al., “Bifunctional Aryliodonium Salts for Highly Efficient Radioiodination and Astatination of Antibodies,” Bioorganic & Medicinal Chemistry 25 (2017): 5975–5980. 28964629 10.1016/j.bmc.2017.09.022 PMC5659727

[101] K. Matsuoka , H. Obata , K. Nagatsu , et al., “Transition‐Metal‐Free Nucleophilic 211At‐Astatination of Spirocyclic Aryliodonium Ylides,” Organic & Biomolecular Chemistry 19 (2021): 5525–5528. 34124736 10.1039/d1ob00789k

[102] C. Maingueneau , M. Berdal , R. Eychenne , et al., “211 At and 125 I‐Labeling of (Hetero)Aryliodonium Ylides: Astatine Wins Again,” Chemistry 28 (2022): e202104169. 34965315 10.1002/chem.202104169

[103] F. Guérard , Y.‐S. Lee , K. Baidoo , J.‐F. Gestin , and M. W. Brechbiel , “Unexpected Behavior of the Heaviest Halogen Astatine in the Nucleophilic Substitution of Aryliodonium Salts,” Chemistry – A European Journal 22 (2016): 12332–12339. 27305065 10.1002/chem.201600922 PMC5013196

[104] M. Tredwell , S. M. Preshlock , N. J. Taylor , et al., “A General Copper‐Mediated Nucleophilic18F Fluorination of Arenes,” Angewandte Chemie International Edition 53 (2014): 7751–7755. 24916101 10.1002/anie.201404436

[105] A. V. Mossine , A. F. Brooks , K. J. Makaravage , et al., “Synthesis of [18F]Arenes via the Copper‐Mediated [18F]Fluorination of Boronic Acids,” Organic Letters 17 (2015): 5780–5783. 26568457 10.1021/acs.orglett.5b02875 PMC4672358

[106] K. J. Makaravage , X. Shao , A. F. Brooks , L. Yang , M. S. Sanford , and P. J. H. Scott , “Copper(II)‐Mediated [11C]Cyanation of Arylboronic Acids and Arylstannanes,” Organic Letters 20 (2018): 1530–1533. 29484880 10.1021/acs.orglett.8b00242 PMC5892454

[107] D. Zhou , W. Chu , T. Voller , and J. A. Katzenellenbogen , “Copper‐Mediated Nucleophilic Radiobromination of Aryl Boron Precursors: Convenient Preparation of a Radiobrominated PARP‐1 Inhibitor,” Tetrahedron Letters 59 (2018): 1963–1967. 30349147 10.1016/j.tetlet.2018.04.024 PMC6195330

[108] S. W. Reilly , M. Makvandi , K. Xu , and R. H. Mach , “Rapid Cu‐Catalyzed [211At]Astatination and [125I]Iodination of Boronic Esters at Room Temperature,” Organic Letters 20 (2018): 1752–1755. 29561158 10.1021/acs.orglett.8b00232 PMC5973503

[109] M. Berdal , S. Gouard , R. Eychenne , et al., “Investigation on the Reactivity of Nucleophilic Radiohalogens With Arylboronic Acids in Water: Access to an Efficient Single‐Step Method for the Radioiodination and Astatination of Antibodies,” Chemical Science 12 (2021): 1458–1468. 10.1039/d0sc05191h PMC8179031 34163909

[110] S. Watanabe , Y. Kondo , I. Sasaki , Y. Ohshima , H. Kimura , and N. S. Ishioka , “Copper‐Mediated Astatination of 211At‐Labelled Prostate‐Specific Membrane Antigen Probes in the Presence of Basic Salts,” Tetrahedron 156 (2024): 133920.

[111] G.‐J. Meyer , K. Roessler , and G. Stoecklin , “Reaction of Aromatic Diazonium Salts With Carrier‐Free Radioiodine and Astatine. Evidence for Complex Formation,” Journal of the American Chemical Society 101 (1979): 3121–3123.

[112] G. M. Visser and E. L. Diemer , “The Reaction of Astatine With Aromatic Diazonium Compounds,” Radiochem Radioanal Letters 51 (1982): 135.

[113] G. Wunderlich , S. Fischer , R. Dreyer , and W. G. Franke , “A Simple Method for Labelling Proteins With 211At via Diazotized Aromatic Diamine,” Journal of Radioanalytical and Nuclear Chemistry Letters 117 (1987): 197–203.

[114] G. W. M. Visser , E. L. Diemer , and F. M. Kaspersen , “The Preparation and Stability of Astatotyrosine and Astato‐Iodotyrosine,” International Journal of Applied Radiation and Isotopes 30 (1979): 749–752.

[115] L. Bo‐Li , J. Yu‐Tai , L. Zheng‐Hao , L. Cheng , K. Masaharu , and M. Minoru , “Halogen Exchanges Using Crown Ethers: Synthesis and Preliminary Biodistribution of,” International Journal of Applied Radiation and Isotopes 36 (1985): 561–563. 2933343 10.1016/0020-708x(85)90110-3

[116] L. Vasaros , Y. V. Norseyev , and V. A. Khalkin , 1980.

[117] L. Vasaros , Y. V. Norseyev , D. D. Nhan , and V. A. Khalkin , “About Possible Nature of Univalent Astatinium Cation Entering the Electrophilic Aromatic Substitution in Heterogeneous Medium in the Presence of Acids,” Radiochemical and Radioanalytical Letters 54 (1982): 239.

[118] Y. V. Norseyev , D. D. Nhan , V. A. Khalkin , N. Q. Huan , and L. Vasaros , “The Preparation of Astatine Labelled Tyrosine Using an Electrophilic Reaction,” Journal of Radioanalytical and Nuclear Chemistry Letters 94 (1985): 185–190.

[119] G. W. M. Visser , E. L. Diemer , and F. M. Kaspersen , “The Preparation and Stability of 211At‐Astato‐Imidazoles,” International Journal of Applied Radiation and Isotopes 31 (1980): 275–278.

[120] I. Brown , “6‐211At‐Astato‐2‐Methyl‐1,4‐Naphthoquinol Bis (Disodium Phosphate): A Novel α‐emitting Potential Anti‐Tumour Drug,” International Journal of Applied Radiation and Isotopes 33 (1982): 75–76. 7061164 10.1016/0020-708x(82)90209-5

[121] G. M. Visser , E. L. Diemer , and F. M. Kaspersen , “The Preparation of Aromatic Astatine Compounds Through Aromatic Mercury‐Compounds,” Journal of Labelled Compounds and Radiopharmaceuticals 17 (1979): 657.

[122] G. W. M. Visser and E. L. Diemer , “The Synthesis of Organic at‐Compounds Through Thallium Compounds,” International Journal of Applied Radiation and Isotopes 33 (1982): 389–390.

[123] R. A. Milius , W. H. McLaughlin , R. M. Lambrecht , et al., “Organoastatine Chemistry. Astatination via Electrophilic Destannylation,” International Journal of Radiation Applications and Instrumentation. Part A, Applied Radiation and Isotopes 37 (1986): 799–802. 3021681 10.1016/0883-2889(86)90274-1

[124] S. Palm , T. Bäck , E. Aneheim , et al., “Evaluation of Therapeutic Efficacy of 211At‐Labeled Farletuzumab in an Intraperitoneal Mouse Model of Disseminated Ovarian Cancer,” Translational Oncology 14 (2021): 100873. 32987283 10.1016/j.tranon.2020.100873 PMC7522120

[125] R. C. Mease , C. M. Kang , V. Kumar , et al., “An Improved 211At‐Labeled Agent for PSMA‐Targeted α‐Therapy,” Journal of Nuclear Medicine 63 (2022): 259–267. 34088772 10.2967/jnumed.121.262098 PMC8805774

[126] A. P. Kiess , I. Minn , G. Vaidyanathan , et al., “2S)‐2‐(3‐(1‐Carboxy‐5‐(4‐211At‐Astatobenzamido)Pentyl)Ureido)‐Pentanedioic Acid for PSMA‐Targeted α‐Particle Radiopharmaceutical Therapy,” Journal of Nuclear Medicine 57 (2016): 1569–1575. 27230930 10.2967/jnumed.116.174300 PMC5367442

[127] M. R. Zalutsky and A. S. Narula , “Astatination of Proteins Using an N‐Succinimidyl Tri‐N‐Butylstannyl Benzoate Intermediate,” International Journal of Radiation Applications and Instrumentation. Part A, Applied Radiation and Isotopes 39 (1988): 227–232. 2836342 10.1016/0883-2889(88)90176-1

[128] A. Orlova , A. Sjöstrom , O. Lebeda , H. Lundqvist , J. Carlsson , and V. Tolmachev , “Targeting Against Epidermal Growth Factor Receptors. Cellular Processing of Astatinated EGF After Binding to Cultured Carcinoma Cells,” Anticancer Research 24 (2004): 4035–4041. 15736449

[129] A. S. Narula and M. R. Zalutsky , “No‐Carrier‐Added Astatination of N‐succinimidyl‐3‐(tri‐n‐butylstannyl) Benzoate (ATE) via Electrophilic Destannylation,” Radiochimica Acta 47 (1989): 131.

[130] G. Vaidyanathan , D. J. Affleck , D. D. Bigner , and M. R. Zalutsky , “N‐Succinimidyl 3‐[211At]Astato‐4‐Guanidinomethylbenzoate: An Acylation Agent for Labeling Internalizing Antibodies With α‐particle Emitting 211At,” Nuclear Medicine and Biology 30 (2003): 351–359. 12767391 10.1016/s0969-8051(03)00005-2

[131] R. H. Larsen , S. P. Hassfjell , P. Hoff , et al., “211At‐Labelling of Polymer Particles for Radiotherapy: Synthesis, Purification and Stability,” Journal of Labelled Compounds and Radiopharmaceuticals 33 (1993): 977–986.

[132] R. H. Larsen , K. M. Murud , G. Akabani , P. Hoff , O. S. Bruland , and M. R. Zalutsky , “211At‐ and 131I‐Labeled Bisphosphonates With High In Vivo Stability and Bone Accumulation,” Journal of Nuclear Medicine: Official Publication, Society of Nuclear Medicine 40 (1999): 1197–1203. 10405142

[133] G. Vaidyanathan , D. Affleck , P. Welsh , A. Srinivasan , M. Schmidt , and M. R. Zalutsky , “Radioiodination and Astatination of Octreotide by Conjugation Labeling,” Nuclear Medicine and Biology 27 (2000): 329–337. 10938466 10.1016/s0969-8051(00)00098-6

[134] E. Aneheim , M. R. S. Foreman , H. Jensen , and S. Lindegren , “N‐[2‐(Maleimido)Ethyl]‐3‐(Trimethylstannyl)Benzamide, a Molecule for Radiohalogenation of Proteins and Peptides,” Applied Radiation and Isotopes 96 (2015): 1–5. 25474765 10.1016/j.apradiso.2014.11.007

[135] M. El Fakiri , N. Ayada , M. Müller , et al., “Development and Preclinical Evaluation of [211At]PSAt‐3‐Ga: An Inhibitor for Targeted α‐Therapy of Prostate Cancer,” Journal of Nuclear Medicine 65 (2024): 593–599. 38423784 10.2967/jnumed.123.267043

[136] M. Muller , U. M. Battisti , M. Zabrocki , et al., “Rapid Electrophilic 211At‐Astatination of Trimethylgermyl Arenes,” ChemPlusChem 89, no. 9 (2024): e202400254. 38877386 10.1002/cplu.202400254

[137] Y. Dekempeneer , T. Bäck , E. Aneheim , et al., “Labeling of Anti‐HER2 Nanobodies With Astatine‐211: Optimization and the Effect of Different Coupling Reagents on Their in Vivo Behavior,” Molecular Pharmaceutics 16 (2019): 3524–3533. 31268724 10.1021/acs.molpharmaceut.9b00354

[138] H. Azizian , C. Eaborn , and A. Pidcock , “Synthesis of Organotrialkylstannanes. The Reaction Between Organic Halides and Hexaalkyldistannanes in the Presence of Palladium Complexes,” Journal of Organometallic Chemistry 215 (1981): 49–58.

[139] H. Gilman and S. D. Rosenberg , “The Preparation of Some Trialkyltin‐Lithium Compounds,” Journal of the American Chemical Society 75 (1953): 2507–2509.

[140] P. S. Gribanov , Y. D. Golenko , M. A. Topchiy , L. I. Minaeva , A. F. Asachenko , and M. S. Nechaev , “Stannylation of Aryl Halides, Stille Cross‐Coupling, and One‐Pot, Two‐Step Stannylation/Stille Cross‐Coupling Reactions Under Solvent‐Free Conditions,” European Journal of Organic Chemistry 2018 (2018): 120–125.

[141] G. Vaidyanathan and M. R. Zalutsky , “1‐(Meta‐[211At]Astatobenzyl)Guanidine: Synthesis via Astato Demetalation and Preliminary In Vitro and In Vivo Evaluation,” Bioconjugate Chemistry 3 (1992): 499–503. 1463779 10.1021/bc00018a006

[142] S. Watanabe , M. A. U. Azim , I. Nishinaka , et al., “A Convenient and Reproducible Method for the Synthesis of Astatinated 4‐[211At]Astato‐l‐Phenylalanineviaelectrophilic Desilylation,” Organic & Biomolecular Chemistry 17 (2019): 165–171. 10.1039/c8ob02394h 30534678

[143] S. Lindegren , S. Frost , T. Bäck , E. Haglund , J. Elgqvist , and H. Jensen , “Direct Procedure for the Production of 211At‐Labeled Antibodies With an ε‐Lysyl‐3‐(Trimethylstannyl)Benzamide Immunoconjugate,” Journal of Nuclear Medicine 49 (2008): 1537–1545. 18703591 10.2967/jnumed.107.049833

[144] S. E. Denmark and A. Ambrosi , “Why You Really Should Consider Using Palladium‐Catalyzed Cross‐Coupling of Silanols and Silanolates,” Organic Process Research & Development 19 (2015): 982–994. 26478695 10.1021/acs.oprd.5b00201 PMC4608042

[145] M. E. Fakiri , N. Ayada , M. Müller , et al., “Development and Preclinical Evaluation of [211At]PSAt‐3‐Ga: An Inhibitor for Targeted a‐Therapy of Prostate Cancer,” Journal of Nuclear Medicine 65, no. 4 (2024): 267043. 10.2967/jnumed.123.267043 38423784

[146] S. M. Moerlein , C. A. Mathis , and Y. Yano , “Comparative Evaluation of Electrophilic Aromatic Iododemetallation Techniques for Labeling Radiopharmaceuticals With Iodine‐122,” International Journal of Radiation Applications and Instrumentation. Part A, Applied Radiation and Isotopes 38 (1987): 85–90. 3032864 10.1016/0883-2889(87)90001-3

[147] H. Bloux , A. Dahiya , A. Hébert , F. Fabis , F. Schoenebeck , and T. Cailly , “Base‐Mediated Radio‐Iodination of Arenes by Using Organosilane and Organogermane as Radiolabelling Precursors,” Chemistry – A European Journal 29 (2023): e202203366. 36607172 10.1002/chem.202203366

[148] D. Teze , D. C. Sergentu , V. Kalichuk , et al., “Targeted Radionuclide Therapy With Astatine‐211: Oxidative Dehalogenation of Astatobenzoate Conjugates,” Scientific Reports 7 (2017): 2579. 28566709 10.1038/s41598-017-02614-2 PMC5451414

[149] Y. Li , M. K. Chyan , D. K. Hamlin , H. Nguyen , E. Corey , and D. S. Wilbur , “Oxidation of p‐[125I]Iodobenzoic Acid and p‐[211At]Astatobenzoic Acid Derivatives and Evaluation in Vivo,” International Journal of Molecular Sciences 23 (2022): 10655. 36142567 10.3390/ijms231810655 PMC9506049

[150] T. Rogova , E. Ahrweiler , M. D. Schoetz , and F. Schoenebeck , “Recent Developments With Organogermanes: Their Preparation and Application in Synthesis and Catalysis,” Angewandte Chemie International Edition 63 (2024): e202314709. 37899306 10.1002/anie.202314709

[151] J. Gao , M. Li , J. Yin , et al., “The Different Strategies for the Radiolabeling of [211At]‐Astatinated Radiopharmaceuticals,” Pharmaceutics 16 (2024): 738. 38931860 10.3390/pharmaceutics16060738 PMC11206656

[152] T. Dong , Z. Zhang , W. Li , W. Zhuo , T. Cui , and Z. Li , “Synthesis Principle and Practice With Radioactive Iodines and Astatine: Advances Made so Far,” Journal of Organic Chemistry 89, no. 17 (2024): 11837–11863. 39173032 10.1021/acs.joc.4c00593

[153] D. Kersting , S. Morbelli , S. E. M. Veldhuijzen van Zanten , and H. J. Verberne , “Highlights of the 36th EANM Annual Congress 2023, From Hometown Vienna, Austria: “ ‘A Symphony Of Science’,” European Journal of Nuclear Medicine and Molecular Imaging 51 (2024): 1800–1808. 38570358 10.1007/s00259-024-06692-z

[154] U. M. Battisti , V. Shalgunov , C. B. M. Poulie , et al., “EANM’23 Abstract Book Congress Sep 9‐13, 2023,” European Journal of Nuclear Medicine and Molecular Imaging 50 (2023): 279. 10.1007/s00259-023-06333-x 37594496

[155] J. Spetz , N. Rudqvist , and E. Forssell‐Aronsson , “Biodistribution and Dosimetry of Free 211At, 125I‐ and 131I‐ in Rats,” Cancer Biotherapy & Radiopharmaceuticals 28 (2013): 657–664. 23789969 10.1089/cbr.2013.1483 PMC3793652

[156] A. Agrawal , V. Rangarajan , S. Shah , A. Puranik , and N. Purandare , “MIBG (Metaiodobenzylguanidine) Theranostics in Pediatric and Adult Malignancies,” British Journal of Radiology 91 (2018): 20180103. 30048149 10.1259/bjr.20180103 PMC6475939

[157] T. Watabe , K. Kaneda‐Nakashima , Y. Shirakami , et al., “Targeted Alpha Therapy Using Astatine (211At)‐Labeled Phenylalanine: A Preclinical Study in Glioma Bearing Mice,” Oncotarget 11 (2020): 1388–1398. 32341757 10.18632/oncotarget.27552 PMC7170498

[158] S. Carlin , G. Akabani , and M. R. Zalutsky , “In Vitro Cytotoxicity of (211)At‐Astatide and (131)I‐Iodide to Glioma Tumor Cells Expressing the Sodium/Iodide Symporter,” Journal of Nuclear Medicine: Official Publication, Society of Nuclear Medicine 44 (2003): 1827–1838. 14602867

[159] S. Carlin , R. J. Mairs , P. Welsh , and M. R. Zalutsky , “Sodium‐Iodide Symporter (NIS)‐Mediated Accumulation of [211At]Astatide in NIS‐Transfected Human Cancer Cells,” Nuclear Medicine and Biology 29 (2002): 729–739. 12381453 10.1016/s0969-8051(02)00332-3

[160] L. T. Burka , T. M. Plucinski , and T. L. Macdonald , “Mechanisms of Hydroxylation by Cytochrome P‐450: Metabolism of Monohalobenzenes by Phenobarbital‐Induced Microsomes,” Proceedings of the National Academy of Sciences 80 (1983): 6680–6684. 10.1073/pnas.80.21.6680 PMC391234 6579552

[161] J. Choi , G. Vaidyanathan , E. Koumarianou , C. M. Kang , and M. R. Zalutsky , “Astatine‐211 Labeled anti‐HER2 5F7 Single Domain Antibody Fragment Conjugates: Radiolabeling and Preliminary Evaluation,” Nuclear Medicine and Biology 56 (2018): 10–20. 29031230 10.1016/j.nucmedbio.2017.09.003 PMC5732883

[162] G. Vaidyanathan , R. C. Mease , I. Minn , et al., “Synthesis and Preliminary Evaluation of 211At‐Labeled Inhibitors of Prostate‐Specific Membrane Antigen for Targeted Alpha Particle Therapy of Prostate Cancer,” Nuclear Medicine and Biology 94 (2021): 67. 33601187 10.1016/j.nucmedbio.2021.01.002 PMC7987787

[163] D. S. Wilbur , M. K. Chyan , D. K. Hamlin , R. L. Vessella , T. J. Wedge , and M. F. Hawthorne , “Reagents for Astatination of Biomolecules. 2. Conjugation of Anionic Boron Cage Pendant Groups to a Protein Provides a Method for Direct Labeling That Is Stable to in Vivo Deastatination,” Bioconjugate Chemistry 18 (2007): 1226–1240. 17583925 10.1021/bc060345s

[164] G. Vaidyanathan , D. J. Affleck , K. L. Alston , et al., “A Kit Method for the High Level Synthesis of [211At]MABG,” Bioorganic & Medicinal Chemistry 15 (2007): 3430–3436. 17387017 10.1016/j.bmc.2007.03.016 PMC1885228

[165] G. Vaidyanathan and M. R. Zalutsky , “Synthesis of N‐Succinimidyl 4‐Guanidinomethyl‐3‐[*I]Iodobenzoate: A Radio‐Iodination Agent for Labeling Internalizing Proteins and Peptides,” Nature Protocols 2 (2007): 282–286. 17406587 10.1038/nprot.2007.20

[166] G. Vaidyanathan , D. J. Affleck , J. Li , P. Welsh , and M. R. Zalutsky , “A Polar Substituent‐Containing Acylation Agent for the Radioiodination of Internalizing Monoclonal Antibodies: N‐Succinimidyl 4‐Guanidinomethyl‐3‐[131I]Iodobenzoate ([131I]SGMIB,” Bioconjugate Chemistry 12 (2001): 428–438. 11353542 10.1021/bc0001490

[167] U. Schweizer and C. Steegborn , “New Insights Into the Structure and Mechanism of Iodothyronine Deiodinases,” Journal of Molecular Endocrinology 55 (2015): R37–R52. 26390881 10.1530/JME-15-0156

[168] G. Vaidyanathan , R. C. Mease , I. Minn , et al., “Synthesis and Preliminary Evaluation of 211At‐Labeled Inhibitors of Prostate‐Specific Membrane Antigen for Targeted Alpha Particle Therapy of Prostate Cancer,” Nuclear Medicine and Biology 94–95 (2021): 67–80. 10.1016/j.nucmedbio.2021.01.002 PMC7987787 33601187

[169] T. T. Huynh , Y. Feng , R. Meshaw , et al., “PSMA‐Reactive NB7 Single Domain Antibody Fragment: A Potential Scaffold for Developing Prostate Cancer Theranostics,” Nuclear Medicine and Biology 134–135 (2024): 108913. 10.1016/j.nucmedbio.2024.108913 38703588

[170] T. Tago , J. Toyohara , R. Fujimaki , et al., “Effects of 18F‐Fluorinated Neopentyl Glycol Side‐Chain on the Biological Characteristics of Stilbene Amyloid‐β Pet Ligands,” Nuclear Medicine and Biology 94–95 (2021): 38–45. 10.1016/j.nucmedbio.2020.12.008 33493787

[171] M. Tada , Y. Kaizuka , K. Kannaka , et al., “Development of a Neopentyl 211At‐Labeled Activated Ester Providing In Vivo Stable 211At‐Labeled Antibodies for Targeted Alpha Therapy,” ChemMedChem 19, no. 18 (2024): e202400369. 38847493 10.1002/cmdc.202400369

[172] Y. Kaizuka , H. Suzuki , T. Watabe , et al., “Neopentyl Glycol‐Based Radiohalogen‐Labeled Amino Acid Derivatives for Cancer Radiotheranostics,” EJNMMI Radiopharmacy and Chemistry 9 (2024): 17. 38407647 10.1186/s41181-024-00244-4 PMC10897087

[173] I. Sasaki , M. Tada , Z. Liu , et al., “1‐(N,N‐Dialkylcarbamoyl)‐1,1‐Difluoromethanesulfonyl Ester as a Stable and Effective Precursor for a Neopentyl Labeling Group With Astatine‐211,” Organic & Biomolecular Chemistry 21 (2023): 7467–7472. 37670575 10.1039/d3ob00944k

[174] S. F. Barrington and R. Kluge , “FDG PET for Therapy Monitoring in Hodgkin and Non‐Hodgkin Lymphomas,” European Journal of Nuclear Medicine and Molecular Imaging 44 (2017): 97–110. 28411336 10.1007/s00259-017-3690-8 PMC5541086

[175] K. Yaginuma , K. Takahashi , S. Hoshi , et al., “Novel Astatine (211At)‐Labelled Prostate‐Specific Membrane Antigen Ligand With a Neopentyl‐Glycol Structure: Evaluation of Stability, Efficacy, and Safety Using a Prostate Cancer Xenograft Model,” European Journal of Nuclear Medicine and Molecular Imaging 52 (2024): 469–481, 10.1007/s00259-024-06945-x. 39394527 PMC11732874

[176] H. Suzuki , S. Yamashita , S. Tanaka , et al., “An 211At‐Labeled Alpha‐Melanocyte Stimulating Hormone Peptide Analog for Targeted Alpha Therapy of Metastatic Melanoma,” European Journal of Nuclear Medicine and Molecular Imaging 52 (2025): 2107–2117, 10.1007/s00259-024-07056-3. 39828865 PMC12014842

[177] D. S. Wilbur , M. K. Chyan , D. K. Hamlin , H. Nguyen , and R. L. Vessella , “Reagents for Astatination of Biomolecules. 5. Evaluation of Hydrazone Linkers in 211At‐ and 125I‐Labeledcloso‐Decaborate(2‐) Conjugates of Fab′ as a Means of Decreasing Kidney Retention,” Bioconjugate Chemistry 22 (2011): 1089–1102. 21513347 10.1021/bc1005625 PMC3116028

[178] D. S. Wilbur , M. K. Chyan , D. K. Hamlin , and M. A. Perry , “Reagents for Astatination of Biomolecules. 3. Comparison ofcloso‐Decaborate(2‐) andcloso‐Dodecaborate(2‐) Moieties as Reactive Groups for Labeling With Astatine‐211,” Bioconjugate Chemistry 20 (2009): 591–602. 19236022 10.1021/bc800515d PMC2668518

[179] S. Milesz , M. Jovchev , D. Schumann , V. A. Khalkin , and M. Milanov , “The EDTA Complexes of Astatine,” Journal of Radioanalytical and Nuclear Chemistry Letters 127 (1988): 193–198.

[180] S. Milesz , Y. V. Norseev , Z. Szücs , and L. Vasáros , “Characterization of DTPA Complexes and Conjugated Antibodies of Astatine,” Journal of Radioanalytical and Nuclear Chemistry Letters 137 (1989): 365–372.

[181] L. Ning , J. Jiannan , M. Shangwu , C. Hengliu , and Y. Yanping , “Preparation and Premilinary Evaluation of Astatine‐211 Labeled IgG via DTPA Anhydride,” Journal of Radioanalytical and Nuclear Chemistry 227 (1998): 187–190.

[182] A. T. Yordanov , K. Deal , K. Garmestani , et al., “Synthesis and Biodistribution Study of a New 211At‐Calix[4]ARene Complex,” Journal of Labelled Compounds and Radiopharmaceuticals 43 (2000): 1219–1225.

[183] R. H. Larsen , S. Slade , and M. R. Zalutsky , “Blocking [211At]Astatide Accumulation in Normal Tissues: Preliminary Evaluation of Seven Potential Compounds,” Nuclear Medicine and Biology 25 (1998): 351–357. 9639296 10.1016/s0969-8051(97)00230-8

[184] D. Schumann , S. Milesz , M. Jovchev , B. C. So , and V. Khalkin , “Nitrilotriacetate Complex of Univalent Astatine,” Radiochimica Acta 56 (1992): 173.

[185] D. S. Wilbur , M.‐K. Chyan , and D. Hamlin , “An Initial Investigation of Radiolabeling With Higher Oxidation States of Astatine‐211: Evaluation of Chelation With DOTA and NOTA,” Journal of Nuclear Medicine 51 (2010): 1454.

[186] J. D. Burns , E. E. Tereshatov , B. Zhang , et al., “Complexation of Astatine(III) With Ketones: Roles of NO3–Counterion and Exploration of Possible Binding Modes,” Inorganic Chemistry 61 (2022): 12087–12096. 35876142 10.1021/acs.inorgchem.2c00085

[187] W. J. McBride , R. M. Sharkey , H. Karacay , et al., “A Novel Method of18F Radiolabeling for PET,” Journal of Nuclear Medicine 50 (2009): 991–998. 19443594 10.2967/jnumed.108.060418

[188] M. Pruszyński , A. Bilewicz , B. Wąs , and B. Petelenz , “Formation and Stability of Astatide‐Mercury Complexes,” Journal of Radioanalytical and Nuclear Chemistry 268 (2006): 91–94.

[189] M. Pruszyński , A. Bilewicz , and M. R. Zalutsky , “Preparation of Rh[16aneS4‐diol]211At and Ir[16aneS4‐diol]211At Complexes as Potential Precursors for Astatine Radiopharmaceuticals. Part I: Synthesis,” Bioconjugate Chemistry 19 (2008): 958–965. 18338858 10.1021/bc700413r PMC2830614

[190] M. Pruszyński , M. Łyczko , A. Bilewicz , and M. R. Zalutsky , “Stability and In Vivo Behavior of Rh[16aneS 4 ‐diol] 211At Complex: A Potential Precursor for Astatine Radiopharmaceuticals,” Nuclear Medicine and Biology 42 (2015): 439–445. 25687450 10.1016/j.nucmedbio.2014.12.011 PMC4387111

[191] M. Lyczko , M. Pruszynski , A. Majkowska‐Pilip , et al., “211At Labeled Substance P (5–11) as Potential Radiopharmaceutical for Glioma Treatment,” Nuclear Medicine and Biology 53 (2017): 1–8. 28683361 10.1016/j.nucmedbio.2017.05.008

[192] J. Piella , N. G. Bastús , and V. Puntes , “Size‐Controlled Synthesis of Sub‐10‐Nanometer Citrate‐Stabilized Gold Nanoparticles and Related Optical Properties,” Chemistry of Materials 28 (2016): 1066–1075.

[193] J. Tanudji , H. Kasai , M. Okada , T. Ogawa , S. M. Aspera , and H. Nakanishi , “211At on Gold Nanoparticles for Targeted Radionuclide Therapy Application,” Physical Chemistry Chemical Physics 26, no. 17 (2024): 12915–12927. 38629229 10.1039/d3cp05326a

[194] X. Huang , K. Kaneda‐Nakashima , Y. Kadonaga , et al., “Astatine‐211‐Labeled Gold Nanoparticles for Targeted Alpha‐Particle Therapy via Intravenous Injection,” Pharmaceutics 14 (2022): 2705. 36559199 10.3390/pharmaceutics14122705 PMC9782038

[195] E. Sporer , C. B. M. Poulie , S. Lindegren , et al., “Surface Adsorption of the Alpha‐Emitter Astatine‐211 to Gold Nanoparticles Is Stable In Vivo and Potentially Useful in Radionuclide Therapy,” Journal of Nanotheranostics 2 (2021): 196–207.

[196] L. Dziawer , P. Koźmiński , S. Męczyńska‐Wielgosz , et al., “Gold Nanoparticle Bioconjugates Labelled With 211At for Targeted Alpha Therapy,” RSC Advances 7 (2017): 41024–41032.

[197] T. Iwasaki , Y. Tokuda , A. Kotake , et al., “Cellular Uptake and In Vivo Distribution of Polyhistidine Peptides,” Journal of Controlled Release 210 (2015): 115–124. 25980622 10.1016/j.jconrel.2015.05.268

[198] Y. Liu , Z. Zhou , Y. Feng , et al., “Gold Nanostars: A Novel Platform for Developing 211At‐Labeled Agents for Targeted Alpha‐Particle Therapy,” International Journal of Nanomedicine 16 (2021): 7297. 34737567 10.2147/IJN.S327577 PMC8560129

[199] E. Sporer , C. B. M. Poulie , T. Bäck , et al., “Covalent Core‐Radiolabeling of Polymeric Micelles With 125I/211At for Theranostic Radiotherapy,” Nanotheranostics 6 (2022): 388–399. 35912139 10.7150/ntno.71906 PMC9330252

[200] J. Kucka , M. Hruby , C. Konak , J. Kozempel , and O. Lebeda . Applied Radiation and Isotopes 64 (2006): 201. 16154358 10.1016/j.apradiso.2005.07.021

[201] R. Hou , T. Ye , Y. Qin , et al., “Strong Affinity Between Astatine and Silver: An Available Approach to Anchoring 211At in Nanocarrier for Locoregional Oncotherapy,” Langmuir 40 (2024): 23624–23631. 39475623 10.1021/acs.langmuir.4c02150

[202] C. Denk , M. Wilkovitsch , E. Aneheim , et al., “Multifunctional Clickable Reagents for Rapid Bioorthogonal Astatination and Radio‐Crosslinking,” ChemPlusChem 84 (2019): 775–778. 31681526 10.1002/cplu.201900114 PMC6813637

[203] H. Echigo , K. Mishiro , M. Munekane , et al., “Development of Probes for Radiotheranostics With Albumin Binding Moiety to Increase the Therapeutic Effects of astatine‐211 (211At,” European Journal of Nuclear Medicine and Molecular Imaging 51 (2024): 412–421. 37819452 10.1007/s00259-023-06457-0

[204] S. Hirata , K. Mishiro , K. Washiyama , et al., “In Vivo Stability Improvement of Astatobenzene Derivatives by Introducing Neighboring Substituents,” Journal of Medicinal Chemistry 68 (2025): 1540–1552. 39757786 10.1021/acs.jmedchem.4c02188

[205] M. Munekane , T. Fuchigami , and K. Ogawa , “Recent Advances In the Development of 225Ac‐ and 211At‐Labeled Radioligands for Radiotheranostics,” Analytical Sciences 40 (2024): 803–826. 38564087 10.1007/s44211-024-00514-w PMC11035452

[206] J. G. Hamilton , P. W. Durbin , and M. W. Parrott , “Accumulation of Astatine211 by Thyroid Gland in Man,” Experimental Biology and Medicine 86 (1954): 366–369. 10.3181/00379727-86-21100 13177680

[207] X. Liu , X. Chen , Y. Rong , et al., “MET Exon 14 Skipping Mutation, Amplification and Overexpression in Pulmonary Sarcomatoid Carcinoma: A Multi‐Center Study,” Translational Oncology 13 (2020): 100757. 32920328 10.1016/j.tranon.2020.100868 PMC7492996

[208] T. Watabe , K. Kaneda‐Nakashima , K. Ooe , et al., “Extended Single‐Dose Toxicity Study of [211At]NaAt in Mice for the First‐in‐Human Clinical Trial of Targeted Alpha Therapy for Differentiated Thyroid Cancer,” Annals of Nuclear Medicine 35 (2021): 702–718. 33871803 10.1007/s12149-021-01612-9 PMC8134311

[209] T. Watabe , M. Hosono , S. Kinuya , et al., “Manual on the Proper Use of Sodium Astatide ([211At]NaAt) Injections in Clinical Trials for Targeted Alpha Therapy (1st Edition,” Annals of Nuclear Medicine 35 (2021): 753–766. 33978932 10.1007/s12149-021-01619-2 PMC8197710

[210] S. Naka , K. Ooe , Y. Shirakami , et al., “Production of [211At]NaAt Solution Under GMP Compliance for Investigator‐Initiated Clinical Trial,” EJNMMI Radiopharmacy and Chemistry 9 (2024): 29. 38619655 10.1186/s41181-024-00257-z PMC11018728

[211] M. Kobayakawa , T. Shiga , K. Takahashi , et al., “Evaluation of Pharmacokinetics, Safety, and Efficacy of [211At] Meta‐Astatobenzylguanidine ([211At] MABG) in Patients With Pheochromocytoma or Paraganglioma (PPGL): A Study Protocol,” PLoS One 19 (2024): e0303623. 38805424 10.1371/journal.pone.0303623 PMC11132457

[212] V. Batra , M. Samanta , M. Makvandi , et al., “Preclinical Development of [211At]Meta‐ Astatobenzylguanidine ([211At]MABG) as an Alpha Particle Radiopharmaceutical Therapy for Neuroblastoma,” Clinical Cancer Research 28 (2022): 4146–4157. 35861867 10.1158/1078-0432.CCR-22-0400 PMC9475242

[213] T. Watabe , M. Namba , S. Yanagida , et al., “Manual on the Proper Use of the 211At‐Labeled PSMA Ligand ([211At]PSMA‐5) for Clinical Trials of Targeted Alpha Therapy (1st Edition,” Annals of Nuclear Medicine 38 (2024): 329–336. 38548987 10.1007/s12149-024-01916-6 PMC11016504

[214] T. Watabe , K. Kaneda‐Nakashima , Y. Kadonaga , et al., “Preclinical Evaluation of Biodistribution and Toxicity of [211At]PSMA‐5 in Mice and Primates for the Targeted Alpha Therapy against Prostate Cancer,” International Journal of Molecular Sciences 25, no. 11 (2024): 5667. 38891856 10.3390/ijms25115667 PMC11172375

[215] T. Watabe , K. Hatano , S. Naka , et al., “First‐in‐Human SPECT/CT Imaging of [211At]PSMA‐5: Targeted Alpha Therapy in a Patient With Refractory Prostate Cancer,” European Journal of Nuclear Medicine and Molecular Imaging 52 (2024): 2253–2255, 10.1007/s00259-024-07017-w. 39688698 PMC12119378

[216] N. Guo , R. Maurice , D. Teze , et al., “Experimental and Computational Evidence of Halogen Bonds Involving Astatine,” Nature Chemistry 10 (2018): 428–434. 10.1038/s41557-018-0011-1 29556053

[217] K. T. Giju , F. De Proft , and P. Geerlings , “Comprehensive Study of Density Functional Theory Based Properties for Group 14 Atoms and Functional Groups, −XY3(X = C, Si, Ge, Sn, Pb, Element 114; Y = CH3, H, F, Cl, Br, I, At,” Journal of Physical Chemistry A 109 (2005): 2925–2936. 16833611 10.1021/jp050463x

[218] Y. Feng , R. Meshaw , X. G. Zhao , S. Jannetti , G. Vaidyanathan , and M. R. Zalutsky , “Effective Treatment of Human Breast Carcinoma Xenografts With Single‐Dose211At‐Labeled Anti‐HER2 Single‐Domain Antibody Fragment,” Journal of Nuclear Medicine 64 (2023): 124–130. 35618478 10.2967/jnumed.122.264071 PMC9841253

[219] J. Tanudji , H. Kasai , M. Okada , T. Ogawa , S. M. Aspera , and H. Nakanishi , “211At on Gold Nanoparticles for Targeted Radionuclide Therapy Application,” Physical Chemistry Chemical Physics 26 (2024): 12915–12927, 10.1039/d3cp05326a. 38629229

[220] Y. Liu , Z. Zhou , Y. Feng , et al., “Gold Nanostars: A Novel Platform for Developing 211At‐Labeled Agents for Targeted Alpha‐Particle Therapy,” International Journal of Nanomedicine 16 (2021): 7297–7305. 34737567 10.2147/IJN.S327577 PMC8560129

[221] P. Molecular , Pipeline, accessed August 30, 2024, https://www.precisionmol.com/pipeline.

